# Evidence for general size‐by‐habitat rules in actinopterygian fishes across nine scales of observation

**DOI:** 10.1111/ele.13768

**Published:** 2021-06-10

**Authors:** John T. Clarke

**Affiliations:** ^1^ Department of Ecology and Biogeography Faculty of Biological and Veterinary Sciences Nicolaus Copernicus University Toruń Poland; ^2^ Institute of Ecology and Earth Sciences Department of Zoology University of Tartu Tartu Estonia

**Keywords:** size evolution, actinopterygian fishes, marine, freshwater, phenotypic evolution

## Abstract

Identifying environmental predictors of phenotype is fundamentally important to many ecological questions, from revealing broadscale ecological processes to predicting extinction risk. However, establishing robust environment—phenotype relationships is challenging, as powerful case studies require diverse clades which repeatedly undergo environmental transitions at multiple taxonomic scales. Actinopterygian fishes, with 32,000+ species, fulfil these criteria for the fundamental habitat divisions in water. With four datasets of body size (ranging 10,905–27,226 species), I reveal highly consistent size‐by‐habitat‐use patterns across nine scales of observation. Taxa in marine, marine‐brackish, euryhaline and freshwater‐brackish habitats possess larger mean sizes than freshwater relatives, and the largest mean sizes consistently emerge within marine‐brackish and euryhaline taxa. These findings align with the predictions of seven mechanisms thought to drive larger size by promoting additional trophic levels. However, mismatches between size and trophic‐level patterns highlight a role for additional mechanisms, and support for viable candidates is examined in 3439 comparisons.

## Graphical abstract

Establishing environmental predictors of body size is of fundamental importance to ecology. With data from over 27,000 species of fishes, I establish general size rules between the major aquatic habitat types, revealing that various marine‐influenced environments contain larger taxa than those in freshwaters. The findings align with the predictions of seven mechanisms thought to drive larger size by promoting additional trophic levels, yet I demonstrate a need for additional mechanisms, and support for viable candidates is examined in 3439 comparisons.

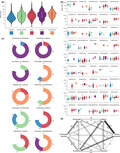

## INTRODUCTION

Considerable attention has been devoted to identifying drivers of biological diversity, commonly from a taxonomic perspective, examining species richness, diversification rates and phylogenetic diversity (Chen & Kishino, 2015; Rabosky et al., 2018). To complement this, biologists also recognise the importance of establishing distributions and correlates of phenotypic and functional diversity, as these offer a vast array of insights, ranging from mapping extinction risk (Cheung et al., 2005; Olden et al., 2007) and quantifying differences in ecosystem function (Flynn et al., 2011), to determining whether species richness or diversification interact with phenotype (Rabosky et al., 2013) and how all of these might be altered by climate change (Buisson et al., 2013).

Establishing phenotypic distributions and their correlates is a priority for body size, a trait known to heavily influence the biology, ecology and extinction risk of species (e.g. Olden et al., 2007). Yet, even for easily accessible traits like size, establishing general patterns is challenging. This is because it can be difficult to find large clades with a robust phylogenetic framework, whose species exhibit several orders of magnitude in size variation and repeatedly explore different habitat types at multiple taxonomic scales. Fortunately, actinopterygian fishes, with ~32,000 species and six orders of magnitude variation in body size, fulfil these criteria for the most fundamental aquatic habitat division; marine vs. freshwater. Distinct evolutionary pressures in these settings provide expectations for how size should vary across this divide (Table S1). For example, the dominance of phytoplankton at the marine food‐web base (relative to freshwater) is associated with longer trophic chains (Potapov et al., 2019) that are expected to sustain larger taxa. Alternatively, freshwater environments may act as refugia that encourage the evolution of large, geologically old taxa termed ‘living fossils’ (Darwin, 1859).

Beyond exclusive focus on marine and freshwater settings, fishes regularly cross this salinity divide, an ability that has given rise to a considerable diversity of euryhaline and brackish taxa, and a macroevolutionary pattern whereby many lineages have explored these varied habitats at several taxonomic scales. This last point is essential, as it allows me to address the duel challenges of (i) ascertainment bias, where results from a single target clade may not be representative of the more general pattern (Beaulieu & O'Meara, 2018); and (ii) the potential for different patterns to arise at different scales of observation (e.g. Hopkins & Smith, 2015), where, for example, important size differences between habitats *within* orders may be obscured by focus on a whole‐dataset‐scale analysis where even larger size differences occur *between* orders. My goal, using four datasets of body size and habitat use (ranging 10,905–27,226 species), is to test for consistent size‐by‐habitat patterns both *within* and *across* nine scales of observation in order to gain a holistic view of size variation in fishes.

## MATERIALS AND METHODS

See Supporting Information for expanded methods, results, and discussion sections.

### Data sources

**FIGURE 1:**
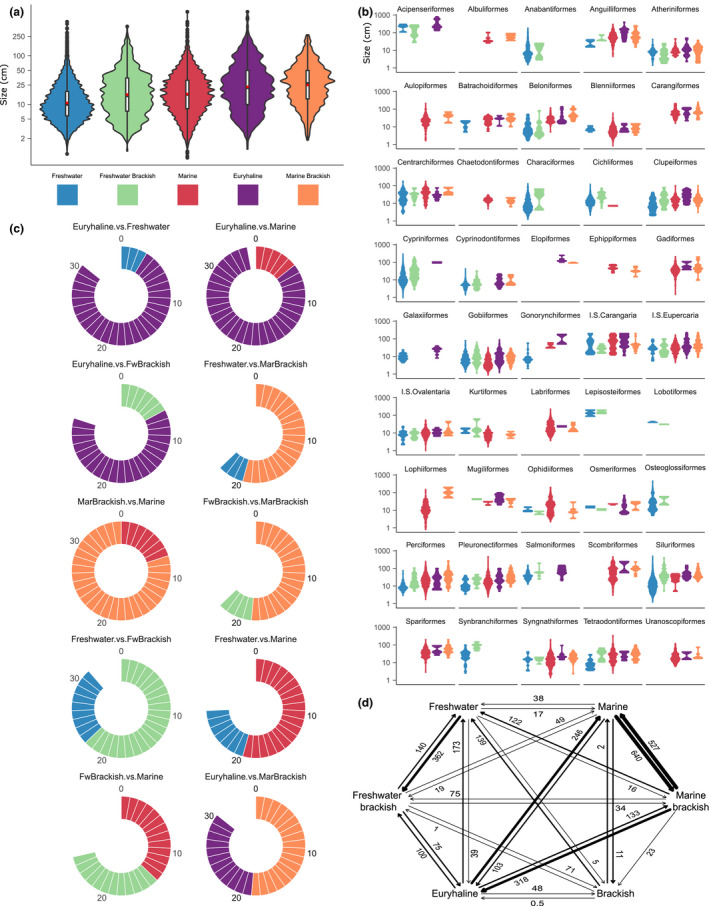
(a) Size distributions (log10 scale) for taxa in each habitat‐use type. (b) Size distributions (log10 scale) in each habitat type for those actinopterygian orders with taxa present in more than one habitat. (c) Illustration of which habitats possess the larger mean size from pairwise comparisons between each habitat type in every order of fishes. Circle completeness represents the total number of orders in which a particular habitat comparison can be made relative to the maximum number observed (maximum number from marine‐brackish vs. marine comparisons, *n* = 35). (d) Average transition counts between each habitat use derived from 100 stochastic character maps under the ARD model in R package phytools. Data from CoF 31k tree matched dataset

Habitat use (commonly shortened to habitat herein) was obtained and analysed from both FishBase (Froese & Pauly, 2019) and Catalogue of Fishes (Fricke et al., 2020) (referred to as FB and CoF herein); the latter is used for main text figures (e.g. Figure 1). Species were assigned to six salinity categories: (i) exclusively marine; (ii) exclusively freshwater; (iii) exclusively brackish; (iv) marine‐brackish, (v) freshwater‐brackish; (vi) euryhaline (Supporting Information text contains further details).

The 11,638‐tip molecular tree and one hundred 31,516‐tip supertrees (from Rabosky et al., 2018, referred to as the 11k and 31k trees herein) were analysed. Size and trophic level data is taken from FishBase (Froese & Pauly, 2019; rfishbase: Boettiger et al., 2012). Given varied overlap in taxon sampling between data sources, four datasets were constructed (Figure S1): two representing the ~10,000 species whose size data match taxa in the 11k tree (herein FB11k dataset [10,905 sp.] and CoF11k dataset [10,195 sp.]) and two representing the ~26,000 species whose size data match taxa in the 31k supertrees (herein FB31k dataset [27,226 sp.] and CoF31k dataset [25,104 sp.]). Analysis files are available on dryad.

A key motivation for performing analyses on both the 11k and 31k trees was to test whether size distributions differ between more representative taxon sampling (31k trees) and taxa selected for molecular sampling (11k tree). Taxa in every habitat were larger on average in the 11k datasets relative to the 31k datasets. Size differences between marine and freshwater habitats were slightly exaggerated in the 11k datasets (see Supporting Information text for further details, including how ‘maximum length’ data compare with a smaller dataset of ‘common length’).

### Scales of observation

Selecting representative taxa for analysis is a fundamental challenge in evolutionary biology (Beaulieu & O'Meara, 2018). As such, this study sought to determine whether size‐by‐habitat patterns were consistent across multiple scales of observation and between molecular trees and supertrees. Nine scales of observation are defined here. Scales 1–7 illustrate patterns at increasingly broad sections of the phylogeny, while scales 8 and 9 represent evolutionary hotspots (see Supporting Information for full details). In short, they represent: family; order; Tax3–Tax6 (where the phylogeny is divided into 13, 9, 5, and 3 sections, respectively); full dataset; evolutionary hotspots; expanded evolutionary hotspots.

### Assessing differences in size between habitats

**FIGURE 2:**
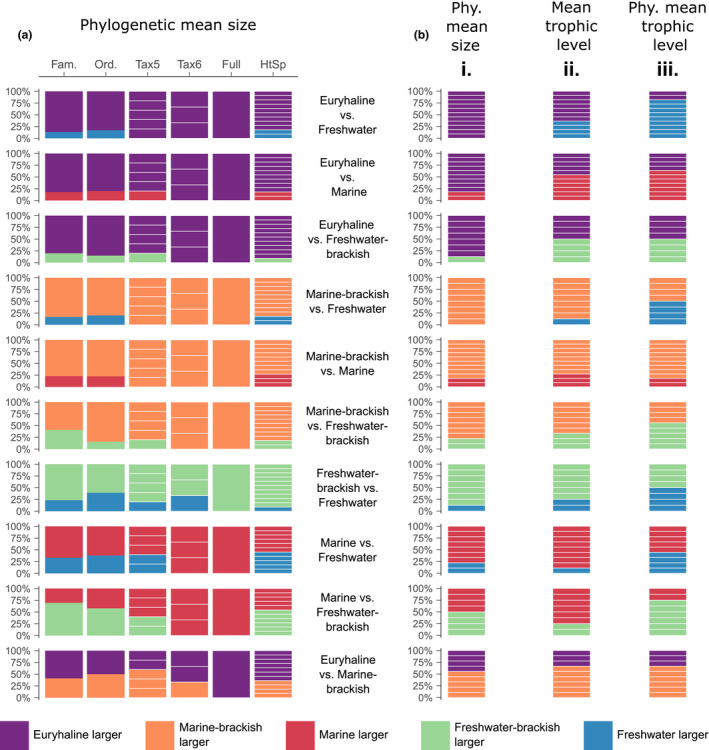
The percentage of clades where the analytical variable for taxa in one habitat is on average larger than for the other habitat under comparison. Comparisons are made between every possible habitat pair across nine scales of observation (five shown, Supporting Information figures show all nine scales [e.g. Figure S3]). (a) comparison of phylogenetic log10 mean size using the entire CoF31k‐tree dataset. (b) comparisons (at Tax3 scale) conducted with the ‘reduced and retained’ CoF31k‐tree dataset (see methods) designed to avoid artefacts from lower sample size, including comparisons of: (bi) phylogenetic log10 mean size (from Appendix 9); (bii) observed log10 mean trophic level; and (biii) phylogenetic log10 mean trophic level (both from Appendix 13). For definitions of taxonomic scales, see methods. Individual clade segments are removed for Fam. and Ord. scales as they contain too many clades to individually visualise; see corresponding appendices for exact clade counts. HtSp = evolutionary hotspots

Five metrics were used to assess differences in log10 body size between habitat‐use types: (i) log10 means; (ii) phylogenetic means (LS mean in R package RRPP); (iii) Wilcoxon tests; (iv) phylogenetic ANOVA (R package phytools [Revell, 2012]); (v) PGLS ANOVA (R package RRPP [Adams & Collyer, 2018]). Each were computed for all 5232 size comparisons in the study. My goal was to clearly visualise percentages of outcomes for a specific pairwise habitat comparison at a specific scale (e.g. in what percentage of clades were marine taxa larger than freshwater taxa at the order scale), so that they could be easily compared with any other habitat comparison and taxonomic scale (e.g. marine vs. euryhaline at family scale). This was achieved visually by scaling sets of comparisons at each taxonomic scale to the same vertical height in the figures regardless of the number of clade comparisons they contained (e.g. see Figure 2a). To combine these features alongside statistical probability information, I opted to colour comparisons with *p* values falling below 0.1 and 0.05 (in different shades) for the Wilcoxon, simulation ANOVA and PGLS ANOVA results (Appendices 2–17). Differences in size variance were also assessed (see Supporting Information text).

### Assessing differences in trophic level between habitats

**FIGURE 3:**
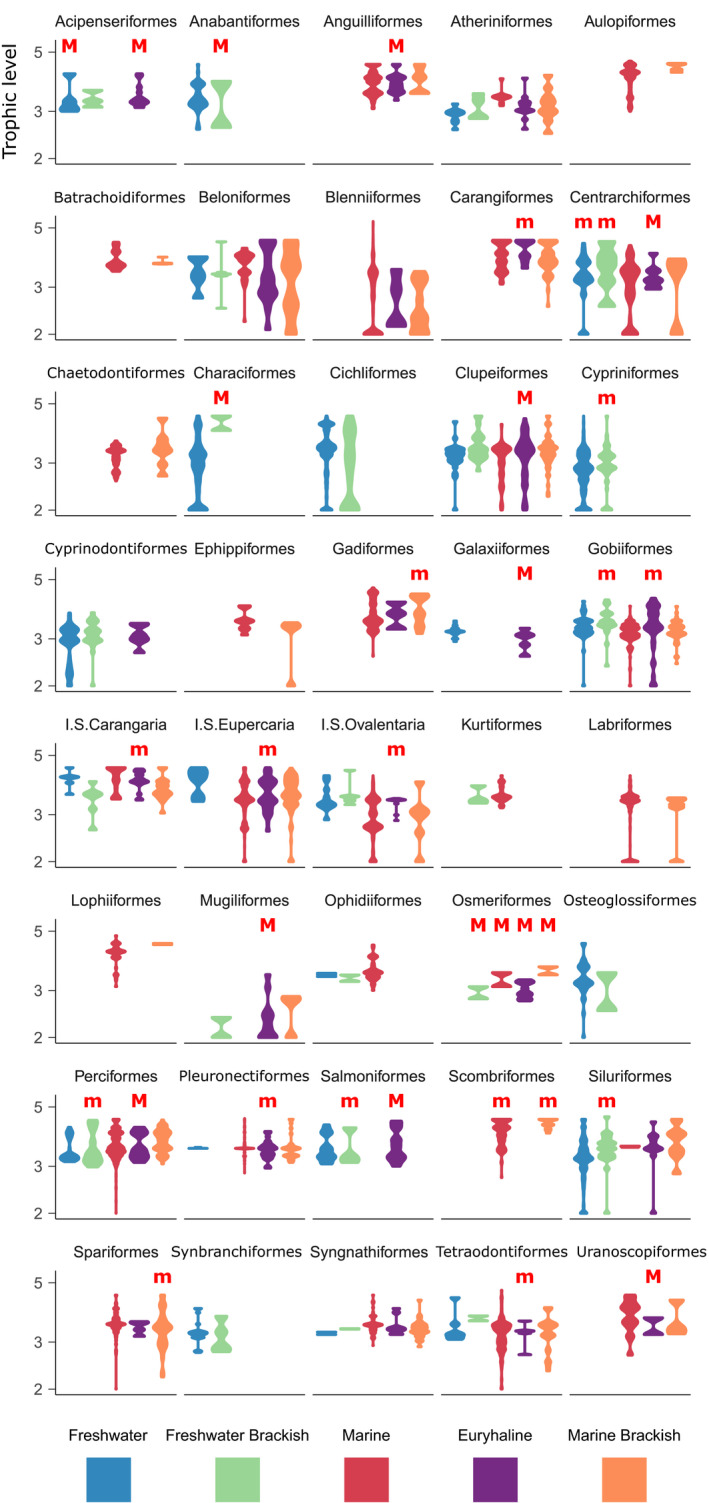
Trophic level distributions (log10 scale) for each habitat type for those actinopterygian orders with taxa present in more than one habitat type. Uppercase ‘M’ and lowercase ‘m’ denote habitats consisting of >30% migratory species, and 5%–30% migratory species, respectively. This displays all taxa shared between the trophic level dataset and CoF 31k‐tree dataset. Thus, some habitat comparisons present here may be absent from the ‘reduced and retained’ datasets that produced Figure 2bii,iii and Appendices 10–13; see methods)

Trophic level distributions were plotted for each habitat and order (Figure 3). Before statistical comparisons of trophic level were made, I reperformed the size analyses above upon smaller size datasets, where the FB11k, CoF11k, FB31k and CoF31k datasets were each pruned to the taxa they shared with the trophic dataset, and only those comparisons whose size outcomes matched those of the original size datasets were retained (Figure 2bi, Appendices 6–9; as illustrated by missing cells in these figures relative to their complete dataset equivalents in Appendices 2–5). This set of retained taxa and habitat comparisons (referred to as ‘reduced and retained’ data in the Results) are the same ones I then analysed for trophic differences (Figure 2bii,iii, Appendices 10–13). This approach provides the fairest way to compare size and trophic outcomes, as it removes any artefactual results introduced by low sample size.

### Assessing correspondence between size, trophic level and other variables

First, the percentage of clades in which a pair of metrics (e.g. size and species richness) were aligned (i.e. both larger or smaller in the same habitat) was calculated for each pairwise habitat comparison. These ‘percentage alignments’ were calculated for every possible comparison of nine metrics (i–iv: mean and phylogenetic size and trophic level; v–vi: observed vs. simulated size variance and trophic variance; vii: species richness; viii: mean tip branch duration; ix: migratory percentage). Results for the order, Tax3 and Tax4 scales are shown in Appendix 1. This revealed which pairs of metrics consistently showed high or low percentage alignments (Table S2). Migratory percentages are shown in Tables S3 and S4. Alignment information was used to deduce, for all 3439 ‘reduced and retained’ habitat comparisons, which suites of mechanisms are supported and how often (Table 1, Tables S5 and S6).

**TABLE 1:** Summary of support for four suites of mechanisms that could plausibly explain the size‐by‐habitat patterns observed here (data from Tax3 scale, CoF31k dataset; see Tables S5 and S6 for other scales and datasets).

Habitat comparison	Phylogenetic trophic level	Migration	Migration w/trophic	% Mig. w/troph.	Migration strong	% Mig. strong	Troph. and Mig. cumulative	Large ‘living fossil’ / Depéret's Rule	Ecological limits reduced	At least one of four mech. suites active
	A	Bi	Bii	Bii′	Biii	Biii′	A + Biii	C	D	A + B + C + D
All Tax3 comparisons (92 in total)	55/92 = 59.8%	21/92 = 22.8%	9/92 = 9.8%	9/21 = 42.9%	12/92 = 13%	12/21 = 57.1%	67/92 = 72.8%	50/92 = 54.3%	51/92 = 55.4%	77/92 = 83.7%
Euryhaline vs. Freshwater	2/11 = 18.2%	6/11 = 54.5%	2/11 = 18.2%	2/6 = 33.3%	4/11 = 36.4%	4/6 = 66.7%	6/11 = 54.5%	6/11 = 54.5%	9/11 = 81.8%	9/11 = 81.8%
Euryhaline vs. Marine	7/11 = 63.6%	5/11 = 45.5%	3/11 = 27.3%	3/5 = 60%	2/11 = 18.2%	2/5 = 40%	9/11 = 81.8%	7/11 = 63.6%	8/11 = 72.7%	10/11 = 90.9%
Euryhaline vs. FwBrackish	4/8 = 50%	2/8 = 25%	0/8 = 0%	0/2 = 0%	2/8 = 25%	2/2 = 100%	6/8 = 75%	4/8 = 50%	1/8 = 12.5%	7/8 = 87.5%
Freshwater vs. MarBrackish	4/8 = 50%	0/8 = 0%	0/8 = 0%	0/0	0/8 = 0%	0/0	4/8 = 50%	5/8 = 62.5%	6/8 = 75%	4/8 = 50%
MarBrackish vs. Marine	11/11 = 100%	1/11 = 9.1%	1/11 = 9.1%	1/1 = 100%	0/11 = 0%	0/1 = 0%	11/11 = 100%	9/11 = 81.8%	8/11 = 72.7%	11/11 = 100%
FwBrackish vs. MarBrackish	6/9 = 66.7%	0/9 = 0%	0/9 = 0%	0/0	0/9 = 0%	0/0	6/9 = 66.7%	4/9 = 44.4%	2/9 = 22.2%	7/9 = 77.8%
Freshwater vs. FwBrackish	3/8 = 37.5%	2/8 = 25%	0/8 = 0%	0/2 = 0%	2/8 = 25%	2/2 = 100%	5/8 = 62.5%	5/8 = 62.5%	7/8 = 87.5%	6/8 = 75%
Freshwater vs. Marine	5/9 = 55.6%	0/9 = 0%	0/9 = 0%	0/0	0/9 = 0%	0/0	5/9 = 55.6%	2/9 = 22.2%	2/9 = 22.2%	7/9 = 77.8%
FwBrackish vs. Marine	5/8 = 62.5%	2/8 = 25%	1/8 = 12.5%	1/2 = 50%	1/8 = 12.5%	1/2 = 50%	6/8 = 75%	4/8 = 50%	3/8 = 37.5%	7/8 = 87.5%
Euryhaline vs. MarBrackish	8/9 = 88.9%	3/9 = 33.3%	2/9 = 22.2%	2/3 = 66.7%	1/9 = 11.1%	1/3 = 33.3%	9/9 = 100%	4/9 = 44.4%	5/9 = 55.6%	9/9 = 100%
Above is an extract from Table S5. These percentages summarise all individual comparisons made at the Tax3 scale from Table S6. 3 example comparisons within the Atheriniformes + Beloniformes + Cyprinodontiformes clade (i.e. 3 of the 92 comparisons made at this scale in the CoF31k dataset) are provided below (1 indicates support for a particular mechanism suite):										
Euryhaline vs. FwBrackish	0	0	0	—	0	—	0	0	0	0
Euryhaline vs. Marine	1	0	0	—	0	—	0	0	0	1
Freshwater vs. Marine	1	0	0	—	0	—	0	0	1	1

Column meanings are as follows: (A) Support for mechanisms influencing trophic level to drive size patterns. Supported in any comparison of two habitats where the habitat whose taxa displayed the larger phylogenetic mean size also occupied a higher phylogenetic mean trophic level relative to the other habitat. (Bi) Support for mechanisms concerning migration to drive size patterns. Supported in any comparison of two habitats where the habitat whose taxa displayed the larger phylogenetic mean size also contained the higher percentage (by at least 20 percentage points) of migrators. (Bii) Support for mechanisms concerning migration and trophic level to drive size patterns simultaneously. Supported in any comparison of two habitats where conditions A and Bi are both met. (Bii') The percentage of Bi outcomes that also fall into the (Bii) category. (Biii) Strong support for mechanisms concerning migration to drive size patterns. Supported in any comparison of two habitats where condition Bi is met, yet trophic level mechanisms (A) are not supported. (Biii') The percentage of Bi ouctomes that also fall into the Biii category. (A+Biii) The cumulative number of comparisons where A and Biii are supported (i.e. combining those comparisons where either trophic level patterns did align with size patterns (A), or trophic patterns did not align, but the larger clade contained at least 20% more migratory species [Biii]). (C) Support for mechanisms where larger mean size is associated with longer geological time (e.g. Depéret's Rule). Supported in any comparison of two habitats where the habitat whose taxa displayed the larger phylogenetic mean size also displayed the larger mean tip branch duration. (D) Support for mechanisms where larger mean size is associated with lower species richness (e.g. far from ecological limits). Supported in any comparison of two habitats where the habitat whose taxa displayed the larger phylogenetic mean size also displayed the lower species richness. (A+B+C+D) The cumulative number of comparisons where A, Bi, C and D are supported (i.e. they need not be supported at the same time, but no comparison is counted more than once). See Tables S5 and S6 for additional columns.

**FIGURE 4:**
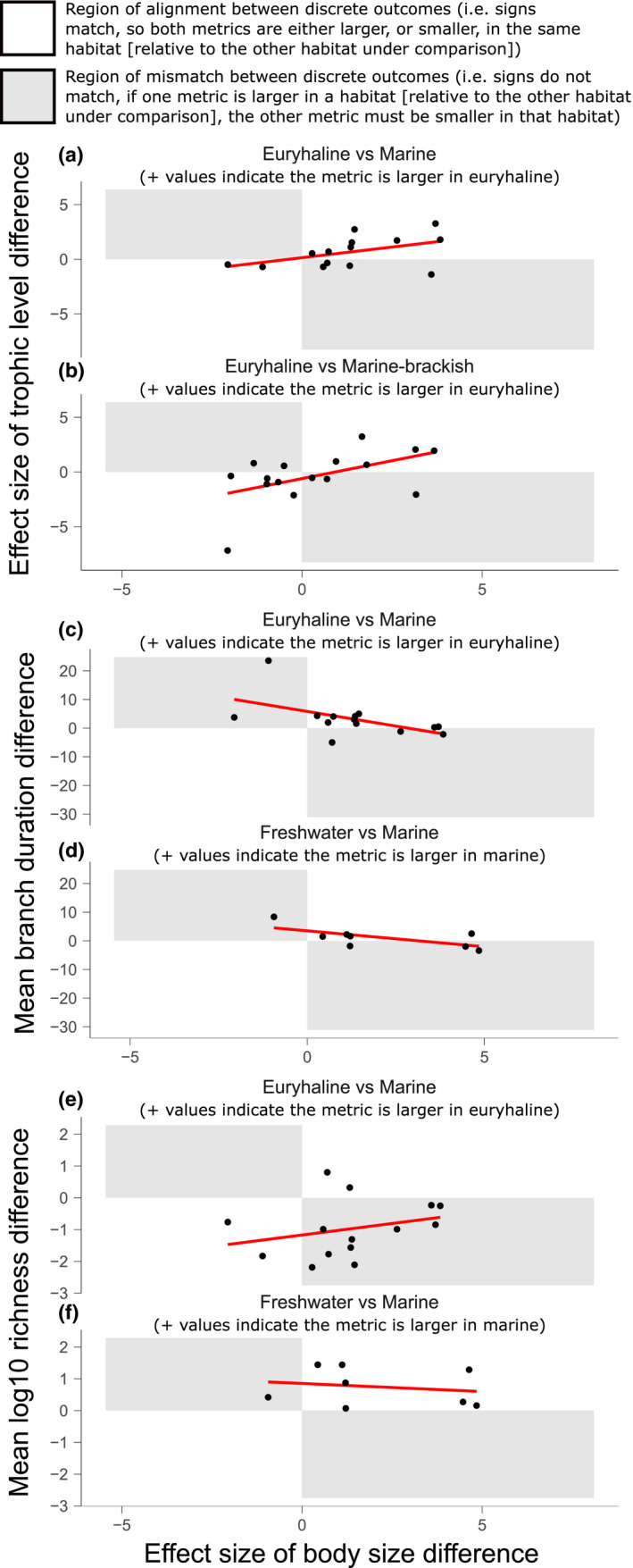
Selected scatterplots to examine whether differences in size between habitats show discrete alignment and proportional correspondence with differences in either trophic level (a, b), mean tip branch duration (c, d), and log10 species richness (e, f). Difference values are given a sign (+ or −) in order to indicate which habitat possessed the larger value. For example, the subheading of plot (a) states that positive (+) values indicate the metric is larger in euryhaline. This means that within plot (a), any clade with a positive value on the *x* axis possesses larger size in their euryhaline members relative to their marine members. The same logic applies to the *y* axis. Thus, for the two metrics compared in (a) (size and trophic level), their outcomes can be said to align/agree when the clade plots in a white quadrant—be it the top right quadrant (euryhaline taxa possess larger size and higher trophic level than their marine relatives) or the bottom left quadrant (euryhaline taxa possess smaller size and lower trophic level than their marine relatives). Conversely, a clade falling within a grey region indicates a mismatch, in a discrete sense, between the size and trophic outcome. For example, for a clade in the bottom right quadrant of (a), the euryhaline members possess larger size, but lower trophic level than their marine members. Thus, consult the subheading of each plot to deduce for which habitat a positive metric indicates larger size/trophic level/mean branch duration/log10 richness in that habitat. The two habitat comparisons selected for each pairwise metric comparison are those notable for either showing regression *p* values below 0.1, or a particularly high or low percentage of discrete alignments. Scatterplots for all habitat comparisons, with order names for every data point, are shown in Figures S6–S8

Second, I examined the continuous relationship of size differences between habitats with corresponding differences in (i) trophic level; (ii) mean tip branch duration; (iii) species richness (Figure 4). This sought to determine the degree to which differences in one metric were joined by corresponding differences in another metric.

## RESULTS

### Size differences between habitats

Full‐dataset size distributions show that freshwater taxa possess the smallest average sizes, and that mean/median size increases through freshwater‐brackish, marine, euryhaline then marine‐brackish habitats (Figure 1a). Distances between habitat means vary, giving a three‐tiered pattern of: (i) small freshwater taxa; (ii) medium sized freshwater‐brackish and marine taxa; (iii) large euryhaline and marine‐brackish taxa. This pattern holds in all datasets (Figure S1) despite the presence of a size bias in 11k‐tree datasets (11k tree contains taxa selected for molecular phylogenetics, which are biased towards larger size; see Supporting Information text). The same size‐by‐habitat pattern is observed within orders of fishes (Figure 1b). Donut charts show how often one habitat mean is larger than another for every order where a specific pairwise habitat comparison can be made (e.g. of 30 orders containing both euryhaline and freshwater taxa, euryhaline taxa have larger mean size in 27; Figure 1c). Ring completeness shows which habitats co‐occur the most (marine and marine‐brackish) and the least (freshwater‐brackish vs. marine‐brackish) across orders. These patterns match what we expect given the species richness of each habitat and the frequency of transitions between them (Figure 1d). Transition frequencies for euryhaline taxa (of which ~30% are migratory, Table S3) in Figure 1d agree with the main findings of Corush (2019), where more transitions out of a migratory lifestyle to marine and freshwater habitats occur than the reverse. Figure 1a–c equivalents for the FB31k dataset (Figure S2) replicate patterns regarding which habitats possessed the larger mean size in (i) the full dataset; and (ii) in a majority of order comparisons.

Size‐by‐habitat patterns are assessed in two ways. First, for each specific pairwise habitat comparison at a specific scale (e.g. euryhaline vs. freshwater, order scale), I examine the percentage of clades where mean taxon size in one habitat is larger than the other (e.g. in a large majority [~80%] of orders, euryhaline taxa possess larger mean size than freshwater taxa; Figure 2a). I therefore commonly refer to the ‘size of the majority’ for a particular outcome. Second, I evaluate the degree to which the direction of these majorities are repeated across multiple taxonomic scales (e.g. euryhaline taxa possess larger mean size than freshwater taxa in large majorities [typically 80% to 100%] across taxonomic scales; Figure 2a).

Considering phylogenetic means, one strong and consistent pattern is that euryhaline and marine‐brackish mean taxon size is larger than for taxa in other habitats (freshwater, marine, freshwater‐brackish); an outcome seen in a majority of comparisons within every taxonomic scale across the datasets (typically ~70%–100%, top six rows of Figure 2a, Figure S3, Appendices 2–5). Freshwater‐brackish mean taxon sizes are larger than freshwater mean sizes in a majority of comparisons within every taxonomic scale across the datasets (typically ~70%–100%, Figure 2a, Figure S3, Appendices 2–5). Furthermore, marine mean taxon sizes are larger than freshwater means in a majority of comparisons within every taxonomic scale across the datasets (~55%–100%, typically ~65%, Figure 2a, Figure S3, Appendices 2–5). Mean size differences between marine taxa and freshwater‐brackish taxa, as well as between marine‐brackish and euryhaline taxa, show no consistent pattern, with different results obtained across different taxonomic scales (Figure 2a, Figure S3, Appendices 2–5). The exact size‐by‐habitat‐use patterns described above are present (typically with larger majority percentages) when comparing non‐phylogenetic log10 means (Supporting Information text, Figure S4). For size variance comparisons (Figure S5), see Supporting Information text.

### Trophic level differences between habitats

Trophic level distributions for each habitat are presented for orders in Figure 3. Trophic‐level‐by‐habitat patterns were tested in an identical manner to the size‐by‐habitat patterns above (Figure 2b, Appendices 10–13), albeit the primary goal was to determine the extent to which trophic patterns align with size patterns (discussed below).

### Quantifying discrete and continuous associations between differences in size, trophic level, branch duration and species richness between habitats

Appendix 1 contains the percentage of clades in which each pair of metrics, from the nine metrics compared between habitats, were aligned (e.g. the percentage of orders where euryhaline taxa possessed both the larger mean size and larger species richness compared to freshwater relatives; an order where euryhaline taxa possessed the smaller mean size and lower species richness also represents an alignment). I commonly refer to these as ‘percentage alignments’ of discrete outcomes. Table S2 contains comparisons whose percentage alignments were either consistently high or consistently low across multiple datasets. Clades whose metrics are aligned for a given habitat comparison fall within the white quadrants of Figure 4, while mismatched outcomes fall within grey quadrants. Establishing these alignments is critical, as they are used to deduce which suites of mechanisms are supported across the 3439 comparisons analysed in this study (Table 1, Tables S5 and S6).

Regarding phylogenetic size and phylogenetic trophic level, outcomes are aligned in 59.8% of habitat comparisons, so that taxa in habitats showing larger mean size (compared to another habitat) occupy the higher mean trophic level (Figure 2bi vs. 2biii, Tax3 scale, Appendix 1, Table 1A, Table S5A; the percentage increases to 68.5% if comparing non‐phylogenetic log10 trophic level, Figure 2bi vs. 2bii, Tax3 scale, Table S5A′). Alignments are consistent with trophic mechanisms influencing size patterns in those comparisons (Table 1A, Tables S5A and S6A). Habitat comparisons displaying the highest percentage alignments (e.g. 71.4%, euryhaline vs. marine, order scale, Table S5A) also display the strongest positive trends in continuous data between the magnitude of size and trophic level differences (Figure 4a,b; Figure S6), such that, as the size difference between two habitats increases, trophic level differences also increase.

Regarding phylogenetic size and tip branch duration, in 54.3% of all habitat comparisons, outcomes are aligned, so that taxa in habitats with larger mean body sizes possess longer mean branch durations (Tax3 scale, Appendix 1, Table 1C, Table S5C). Alignments are consistent with Depéret's Rule in those comparisons (Tables 1C, S1, Tables S5C and S6C). There is however little evidence for positive trends in continuous data between magnitudes of size and branch duration differences between habitats (Figure 4c,d, Figure S7).

Regarding phylogenetic size and log10 species richness, Table S2 reports five habitat comparisons with consistently low percentage alignments between their discrete outcomes (Tax3; 12.5%–27.3%), and two habitat comparisons with consistently high percentage alignments (77.8%). Mismatched outcomes are consistent with predictions regarding ecological limits in those comparisons (Table 1D, Tables S5D and S6D). Figure 4e shows a low percentage alignment example (Appendix 1; 28.6% alignment; CoF31k, order scale), which means that, in the remaining 71.4% of order comparisons, euryhaline taxa possess larger size but lower richness than marine relatives (i.e. mismatched outcomes; Table S5D). Figure 4f shows a high percentage alignment example, illustrating how marine taxa from each clade tend to possess the larger size and higher richness than freshwater relatives (Figure 4f). Despite specific habitat comparisons being either highly mismatched or highly aligned, there are no statistically significant trends in continuous data regarding how magnitudes of size differences correspond to magnitudes of species richness differences (Figure S8).

## DISCUSSION

The diversity of actinopterygian fishes permits repeated tests of size differences between the fundamental aquatic habitat‐use types. The striking central finding is that, despite huge variation in the groups analysed (e.g. in species richness, age) two highly consistent patterns emerge at all scales of observation (Figure 2a): (i) taxa in marine‐influenced habitats (freshwater‐brackish, marine, euryhaline, marine‐brackish) are larger than taxa from freshwater habitats; and (ii) marine‐brackish and euryhaline fishes are larger on average than those utilising any other habitat. These patterns arise from an underlying trend where freshwater taxa possess the smallest mean sizes, then mean taxon size increases through freshwater‐brackish, marine, euryhaline then marine‐brackish settings (Figure 1a; also seen in Mesozoic neopterygians; Figure S9 [Clarke et al., 2016; Clarke & Friedman, 2018]). Some aspects of this pattern have emerged from studies of Belonidae (Kolmann et al., 2020), Clupeiformes (Bloom et al., 2018), subsets of a 5425 species dataset (Sanchez‐Hernandez & Amundsen, 2018) and Late Jurassic–Early Cretaceous fishes (Guinot & Cavin, 2018). The key insight of this study is that whatever mechanism or combination of mechanisms generate these phenomena, they are sufficiently widespread to produce consistent size‐by‐habitat differences at every scale across the actinopterygian Tree of Life. For all 3439 comparisons analysed, support for each of the four main suites of mechanisms discussed below are provided in Table S6 (with mechanisms further discussed in Table S1). Table S6 is summarised in Table S5, with elements of Table S5 most pertinent to the discussion presented in Table 1.

### Assessing the potential for mechanisms influencing trophic level to explain size‐by‐habitat differences

A broadly established correlate of size in fishes is trophic level (Romanuk et al., 2011). Thus, mechanisms where habitat is expected to influence trophic structure may best explain size‐by‐habitat differences. Mechanisms that can increase mean trophic level in one habitat relative to another have been most clearly described between marine and freshwater environments. Potentially the most important mechanism of this type is food‐web structure (Potapov et al., 2019). In marine habitats, phytoplankton commonly dominate the food‐web base. Largely inaccessible to fishes, this resource becomes accessible via primary consumers (e.g. zooplankton), encouraging the development of long trophic chains, with fishes acting as secondary consumers and above. Size increases accompany each level due to gape‐limited prey ingestion. In contrast, when plant material and detritus dominate the food‐web base (common in freshwater habitats), both small and large fishes act as primary consumers, resulting in fewer trophic levels and weaker size structure (Potapov et al., 2019).

I identify six further mechanisms expected to influence mean trophic level in a predictable way between freshwater and marine‐influenced habitats. First, freshwater fishes appear to be more sensitive to the higher energy demands of warm climates, restricting them to lower trophic levels relative to taxa in comparably warm marine‐influenced settings (Dantas et al., 2019). Second, larger ecosystems (e.g. marine‐influenced settings) increase access to available resources by permitting species to feed over a wider area (Pimm, 1982), allowing additional trophic levels to form (Griffiths, 2013; McHugh et al., 2015; Post, 2002; Post et al., 2000; Takimoto et al., 2012). This is expected even if the larger environment possesses equal per‐unit‐area productivity to a smaller environment (productive‐space hypothesis [Schoener, 1989]). Third, the instability of numerous freshwater habitats may hinder development of long trophic chains, due to factors such as habitat disappearance, habitat fragmentation, and undesirable fluctuations in nutrients and temperature from which freshwater species typically have limited capacity to escape (Hurst, 2007). Fourth, the greater age of marine‐influenced environments may foster greater food‐web complexity, facilitating additional trophic levels. Fifth, the greater topological variety of the shallow seafloor (where most marine fish diversity resides [Rabosky et al., 2018]) relative to freshwater environments (Grosberg et al., 2012), particularly regarding depth variation, should promote additional trophic levels (Kortsch et al., 2019). Sixth, the wider variety of trophic levels consumed (a measure of omnivory) in marine environments relative to freshwater (Sanchez‐Hernandez & Amundsen, 2018) may increase the ability for taxa to meet their energy needs from a variety of sources, permitting additional trophic levels. Importantly, all seven mechanisms outlined above act in the same direction: to promote larger mean trophic level (and by association size) in marine‐influenced settings over freshwater. This alignment could help explain the consistency of the size‐by‐habitat patterns.

Given strong expectations that these mechanisms drive size via trophic level, one should expect excellent alignment between both metrics, and this was tested in 3439 habitat comparisons. As would be expected from resemblances between size and trophic level distributions (Figures 1b and 3), size and trophic level outcomes match in 59.8% of comparisons, and this rises to 68.5% if non‐phylogenetic trophic level is compared (Tax3 scale; Appendix 1, Table 1A, Table S5A,A′). This suggests mechanisms influencing size via trophic level alone could theoretically underpin a majority of the size‐by‐habitat pattern. The elevated percentage seen when comparing non‐phylogenetic mean trophic level (at some scales) suggests that marine‐influenced habitats (including euryhaline) do commonly contain additional trophic levels, but that sometimes, the highest levels are occupied by either few or closely related taxa, rather than the whole distribution shifting towards higher values (as consistently occurs for size).

Further evidence for the importance of an association between trophic mechanisms and size patterns can be derived from repeated evidence of positive proportional relationships between the size difference observed between two habitats and the corresponding trophic level difference. These positive relationships are seen in five sets of pairwise habitat comparisons (e.g. euryhaline vs. marine clades represent one set, order scale, Figure 4a,b, Figure S6). Although negative relationships can also occur (Figure S6), they are statistically fragile, underpinned by the position of one or two clades. These decisive clades frequently represent extreme outliers with strongly decoupled size and trophic patterns (e.g. Centrarchiformes, Clupeiformes, Galaxiiformes, Gobiiformes), potentially related to their migratory lifestyles (see ‘Alternative drivers’ section). Overall, evidence for positive proportionality between size differences and trophic level differences is better supported statistically than trends seen between size differences and differences in either tip branch duration, or species richness (Figures S7 and S8). This is because (i) the positive relationship emerges repeatedly in several sets of habitat comparisons; (ii) these trends are not underpinned by one or two outlier clades; and (iii) *r* ^2^ and *p* values are notably high and low (respectively) relative to those seen comparing size differences with branch duration or species richness differences (Figures S7 and S8). This finding, combined with the high percentage of discrete size–trophic alignments, suggests that of the variables examined here (for the purpose of identifying mechanisms that underpin the size patterns) mechanisms associated with trophic level possess the greatest explanatory potential.

### Alternative drivers of observed size‐by‐habitat patterns

Given that discrete size and trophic level outcomes matched in 59.8% of comparisons (Table 1A, Tax3 scale), additional mechanisms must exist to help explain the size‐by‐habitat pattern in at least some of the remaining 40.2% of comparisons. Examining large instances of size–trophic mismatch may help to reveal them, and the largest mismatches mostly occur within euryhaline comparisons (e.g. extreme outliers Clupeiformes and Galaxiiformes, euryhaline vs. freshwater, Figure S6). Bloom et al. (2018) similarly identified a discrepancy in Clupeiformes, discovering that migratory taxa possessed larger size than freshwater and marine relatives, but did not occupy a higher trophic level in their model, which they argued was consistent with selection pressures for alternative benefits of large size in migrators. There is considerable evidence for these selection pressures, given (i) increasing size has been shown to exponentially decrease energetic cost per unit distance, thus improving the efficiency of migration (Bernatchez & Dodson, 1987); (ii) larger size allows for greater energy stores (Roff, 1988; Roff, 1991); and iii) widespread evidence of larger size in migratory taxa across actinopterygians (Burns & Bloom, 2020; Griffiths, 2010), even within individual species (Glebe & Leggett, 1981; Labeelund, 1991).

Given the relatively high proportion of euryhaline migrators (Table S3), selection for alternative advantages of large size could explain why euryhaline taxa are consistently larger than other taxa, even when their trophic level is lower. It is likely these selective forces play a decisive role in determining the size pattern in instances of size–trophic mismatch where the habitat with the larger taxa also contains a notably higher percentage of migrators. At the order scale, this situation occurs in 12%–33% of euryhaline comparisons, and only once in other comparisons (freshwater‐brackish Perciformes are larger than freshwater members but occupy a lower mean trophic level, Figures 1b and 3, Tables S5Biii and S6Biii; Table S1). The strongest example of the above situation (i.e. an instance of size‐trophic mismatch underpinned by the largest difference in migrator percentage) is seen in Galaxiiformes (euryhaline vs. freshwater) (Table S4, Figures 1b and 3).

It is common for taxa in habitats containing notable proportions of migrators to also occupy higher trophic levels than their comparator group (i.e. in 42.9% of Tax3 clades and 45.5% of orders where migratory influence is supported, Table 1Bii′, Table S5Bii′). Indeed, occupation of high trophic levels is commonly expected in migratory taxa, because their abilities to forage over a wider area, and to seek out areas of high productivity (Gross et al., 1988), should help them sustain higher trophic levels. Thus, whether mechanisms inflating trophic level, alternative selection pressures for large size, or both, act in any individual euryhaline clade (Table S6), this diversity of mechanisms promoting large size in migratory taxa likely explain why euryhaline species are consistently larger than species in other habitats.

Additional drivers are still necessary to explain the size‐by‐habitat pattern, given the 27.2% of comparisons where trophic level and migration associated mechanisms are not supported (Tax3 scale, Table 1A + Biii). Two additional suites of mechanisms are explored. First, I quantified how often longer mean tip branch duration of taxa in a habitat aligns with larger size, to jointly evaluate (i) the Darwinian idea that long‐lived lineages commonly manifest as large ‘living fossil’ taxa; and (ii) Depéret's rule (Depéret, 1909) that lineages trend towards larger size over evolutionary time. While alignment occurred in 58.8% and 54.3% of order and Tax3 scale comparisons respectively (Table 1C, Table S5C), increased differences in mean tip branch duration between two habitats did not correspond to proportionally increased size differences (Figure 4c,d, Figure S7). The absence of positive proportional relationships may suggest long‐term evolutionary trends are rarely responsible for the size differences observed between these habitats. This interpretation is consistent with findings that selection towards larger size in migrators is rapid (Burns & Bloom, 2020) and that large‐magnitude size changes occur in the transition lineage between habitats (Guinot & Cavin, 2018). It is therefore possible that the discrete alignments seen between longer duration and larger size occur as a by‐product of alternative mechanisms, such as those influencing trophic level, as elevated branch durations would be expected in older environments that develop more complex food webs containing additional trophic levels (Table S1).

Second, depending upon the two habitats under comparison, the more species rich habitat can either contain predictably larger taxa, or predictably smaller taxa (i.e. high and low percentage alignments respectively, red values in Table S2), suggesting there is no universal direction of effect between clade species richness and size. Despite this variability, the two habitats with the largest taxa are consistently species poor (euryhaline, marine‐brackish), consistent with the idea that their larger size could be facilitated by the reduction of food web constraints expected to limit the number of large species sustainable in species‐rich clades. Despite these habitat specific associations between large size and low species richness, it is incorrect to assume that the distinct size and richness profiles of each habitat map tightly onto a speciation gradient (from high speciation in smaller freshwater species, through to low speciation in larger euryhaline species). Instead, diadromous species (common in euryhaline) possess the highest diversification rates (Corush, 2019), and although freshwater speciation rates exceed those of larger marine taxa (e.g. Tedesco et al., 2017), this appears driven by specific clades, rather than a strong feature across the phylogeny (Rabosky, 2020).

Finally, even if I assume that every comparison where the size outcome is consistent with any of the four suites of mechanisms is fully explained by them (i.e. mechanisms related to trophic level, migration, branch duration and species richness could cumulatively explain 83.7% of comparisons, Table 1A + B + C + D), this would leave 16.3% of size outcomes unexplained. Thus, there is a role for further mechanisms to underpin some of the size‐by‐habitat pattern, and I highlight literature discussing candidates with broad potential. For instance, it has been demonstrated that habitat complexity in fishes is associated with anatomical features allowing greater manoeuvrability, such as smaller size (Larouche et al., 2020). Given the complexity of freshwater environments, due to their fragmentary nature, complex geometries, variable flow regimes and vegetation, it is reasonable to suppose this complexity encourages small manoeuvrable taxa. In addition, the prevalence of still waters across numerous freshwater settings may promote small size and miniaturisation, as larger sizes capable of counteracting currents are not required (Weitzman & Vari, 1988). Furthermore, the presence of larger taxa in marine‐influenced settings through mechanisms inflating trophic level should bring additional selection pressures for large size in order to outgrow, evade, or hunt other predatory species (Webb, 1984).

## CONCLUSIONS

I reveal clear size‐by‐habitat patterns that are striking in their consistency across clades at nine scales of observation. These size patterns also emerge across different datasets and phylogenies, despite the presence of a size bias where taxa sampled for molecular data (11k‐tree taxa) represent a larger subset of a more complete sample (31k supertrees). The consistency of these findings suggests that a variety of mechanisms act with a consistent direction of effect across the five habitat types examined here. It also highlights the benefit of examining patterns upon different phylogeny types and at multiple scales to help overcome varying forms of ascertainment bias (see Supporting Information text discussion).

My analyses, combined with the strongest evidence in the literature, suggest that, of the traits examined here, those indicative of mechanisms causing different numbers of trophic levels to form between habitat types, and selection pressures for benefits of large size in migratory taxa, have the greatest potential to explain the size‐by‐habitat pattern (cumulatively, they are supported in 72.8% of habitat comparisons, Table 1A + Biii). This does however highlight a need for additional mechanisms to minimally help explain the remaining 27.2% of comparisons, and after assessing support for drivers associated with species richness and lineage age, I list several additional mechanisms with broad potential that represent ideal targets for future research. Thus, this effort makes substantial gains, establishing clear size patterns at multiple scales, and evaluating nine variables in order to test support for four suites of mechanisms in 3439 habitat comparisons (Tables S5 and S6). Nevertheless, there remains scope to discover and quantify new selective pressures, and test the relative importance of these alongside those examined here in detailed studies of individual clades of fishes, through a combination of field studies, palaeontological data, and comparative analysis.

## AUTHORSHIP

JTC performed all design, analysis and writing of the manuscript.

### PEER REVIEW

The peer review history for this article is available at https://publons.com/publon/10.1111/ele.13768.

## Supporting information

Fig S1‐S15

Table S1‐S4

Table S5

Table S6

Supplementary Material

Appendix 1

Appendix 2

Appendix 3

Appendix 4

Appendix 5

Appendix 6

Appendix 7

Appendix 8

Appendix 9

Appendix 10

Appendix 11

Appendix 12

Appendix 13

Appendix 14

Appendix 15

Appendix 16

Appendix 17

## Data Availability

Data supporting the results are archived on Dryad https://doi.org/10.5061/dryad.tb2rbnzxs.
