# Supplementary material for: Evidence for general size‐by‐habitat rules in actinopterygian fishes across nine scales of observation
Source: Ecol Lett. 2021 Jun 10;24(8):1569–81. doi: 10.1111/ele.13768 (PMC8362132; doi:10.1111/ele.13768)
Supplement: Supplementary file 1 — Fig S1‐S15 [file ELE-24-1569-s019.pdf]

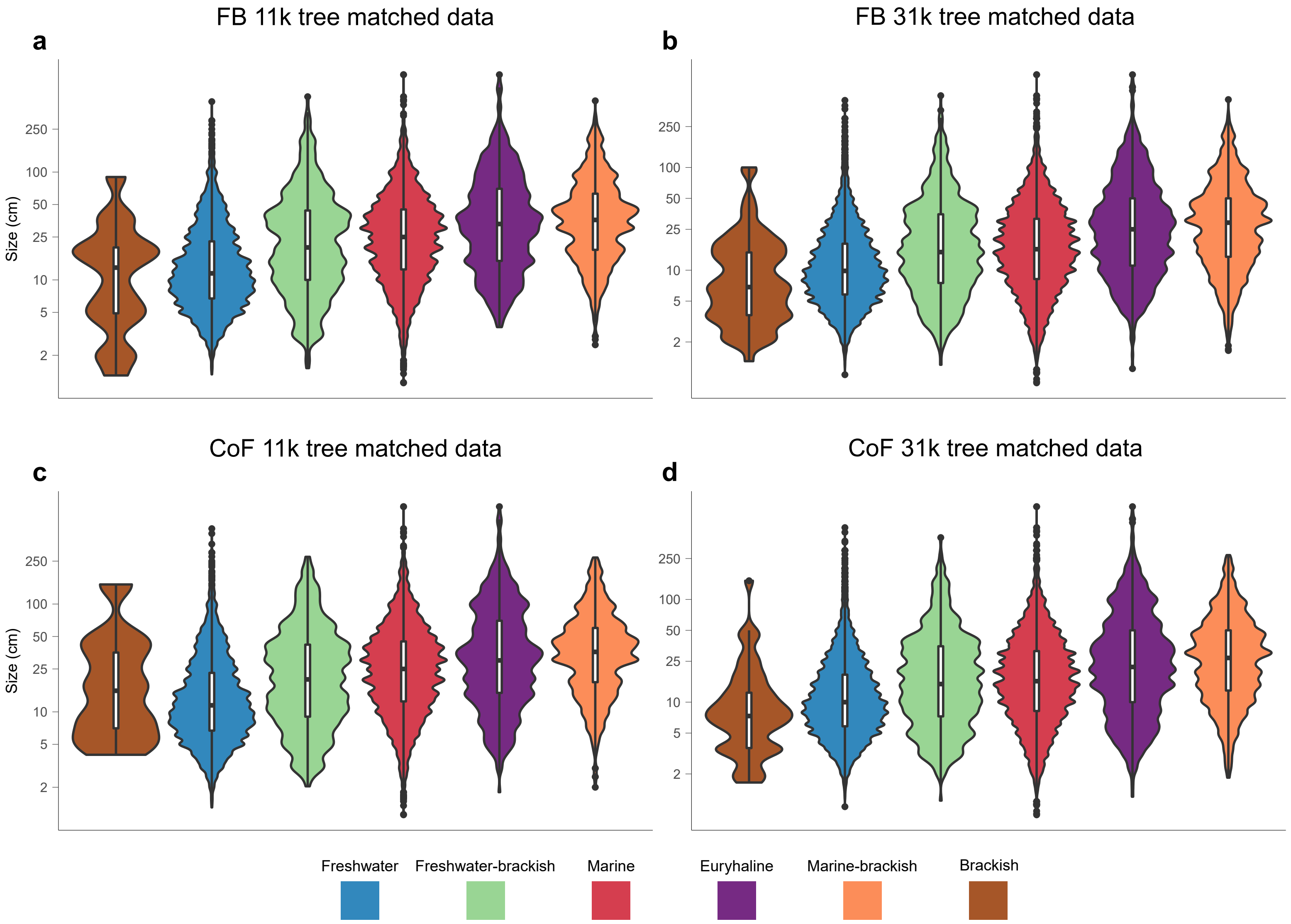


Fig. S1: Size distributions (log10 scale) for taxa in each habitat use across four datasets: (a) ‘FB11k dataset’ (FishBase habitat codings matched with 11k tree and size data: 10905 sp.). (b) ‘CoF11k dataset’ (Catalogue of Fishes habitat codings matched with 11k tree and size data: 10195 sp.). (c) ‘FB31k dataset’ (FishBase habitat codings matched with 31k tree and size data: 27226 sp.). (d) ‘CoF31k dataset’ (Catalogue of Fishes habitat codings matched with 31k tree and size data: 25104 sp.).

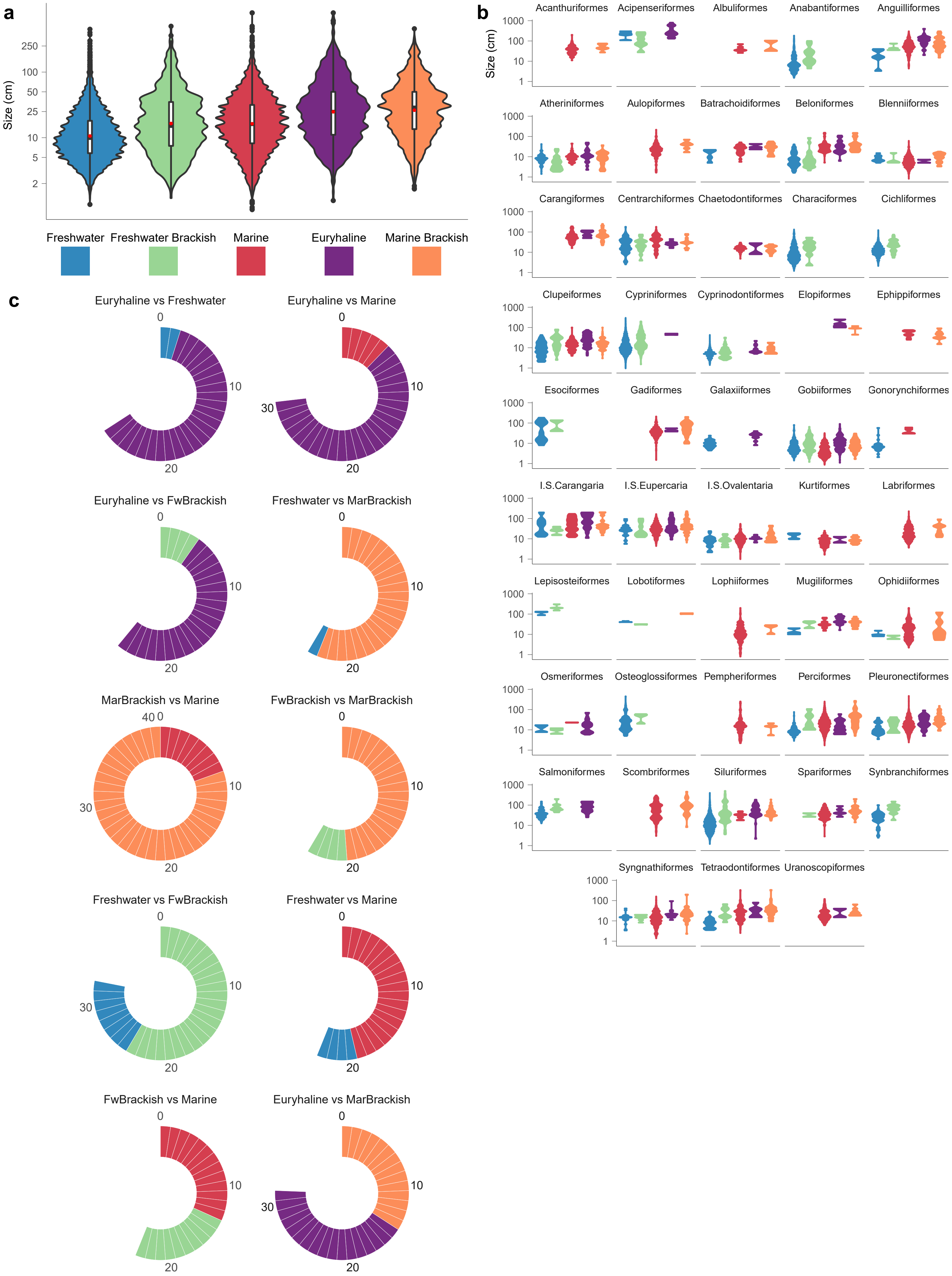


Fig. S2: Corresponding plot to Fig. 1 using FishBase 31k tree dataset. (a) Size distributions (log10 scale) for taxa in each habitat-use type. (b) Size distributions (log10 scale) for taxa in each habitat-use type in each actinopterygian order that has taxa present in more than one habitat. (c) Illustration of which habitats possess the larger mean size from pairwise comparisons between all habitat types in every order of fishes. Circle completeness represents the total number of orders in which a particular habitat comparison can be made relative to the maximum number seen (attained by marine-brackish vs. marine comparisons).

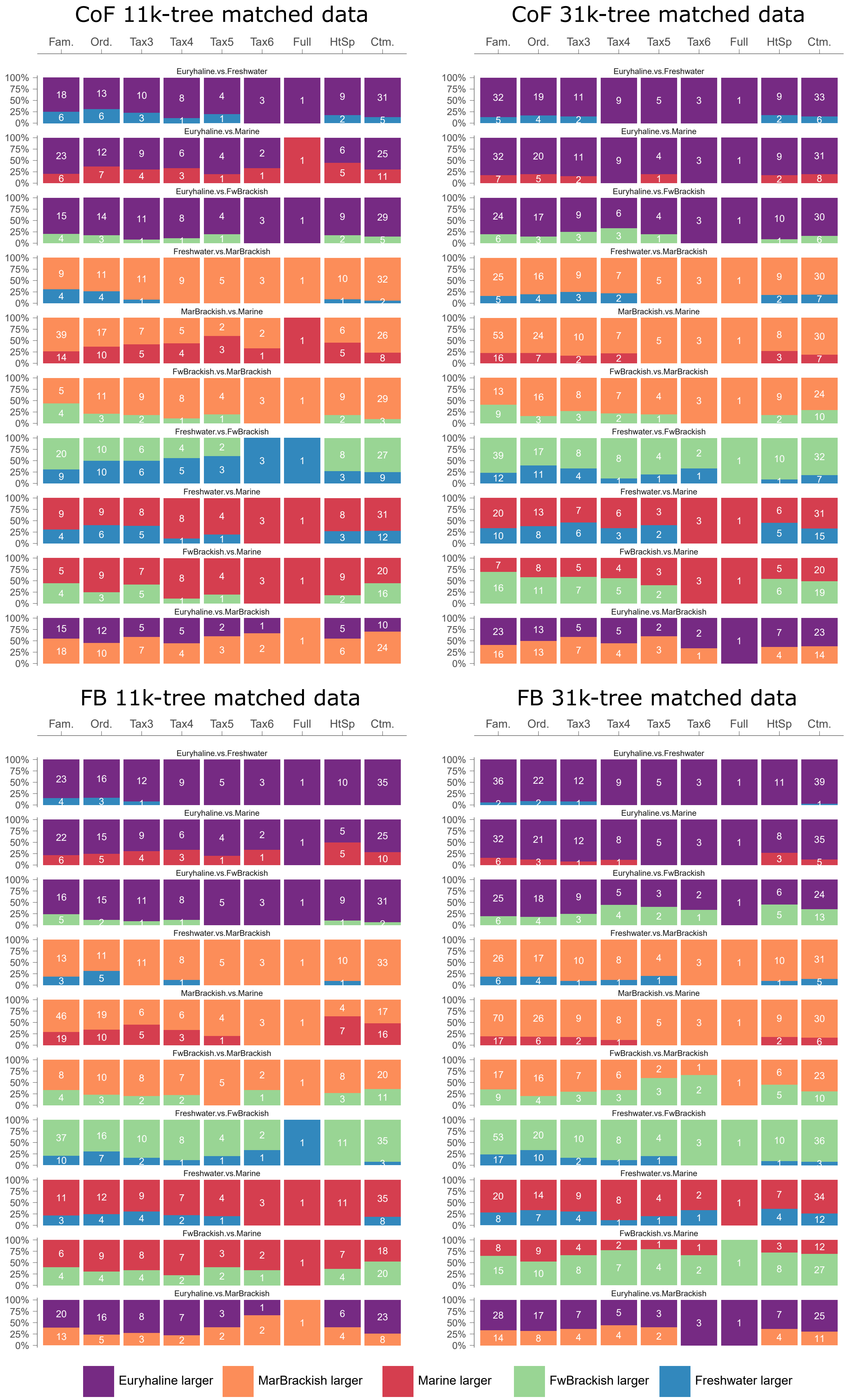


Fig. S3: The percentage of groups where the phylogenetic mean size of taxa for one habitat use is larger than the other, obtained for every pairwise habitat-use comparison across multiple taxonomic scales. These represent the equivalent figures to those in Fig. 2a, but for all four datasets (FB11k, CoF11k and FB31k and CoF31k tree datasets).

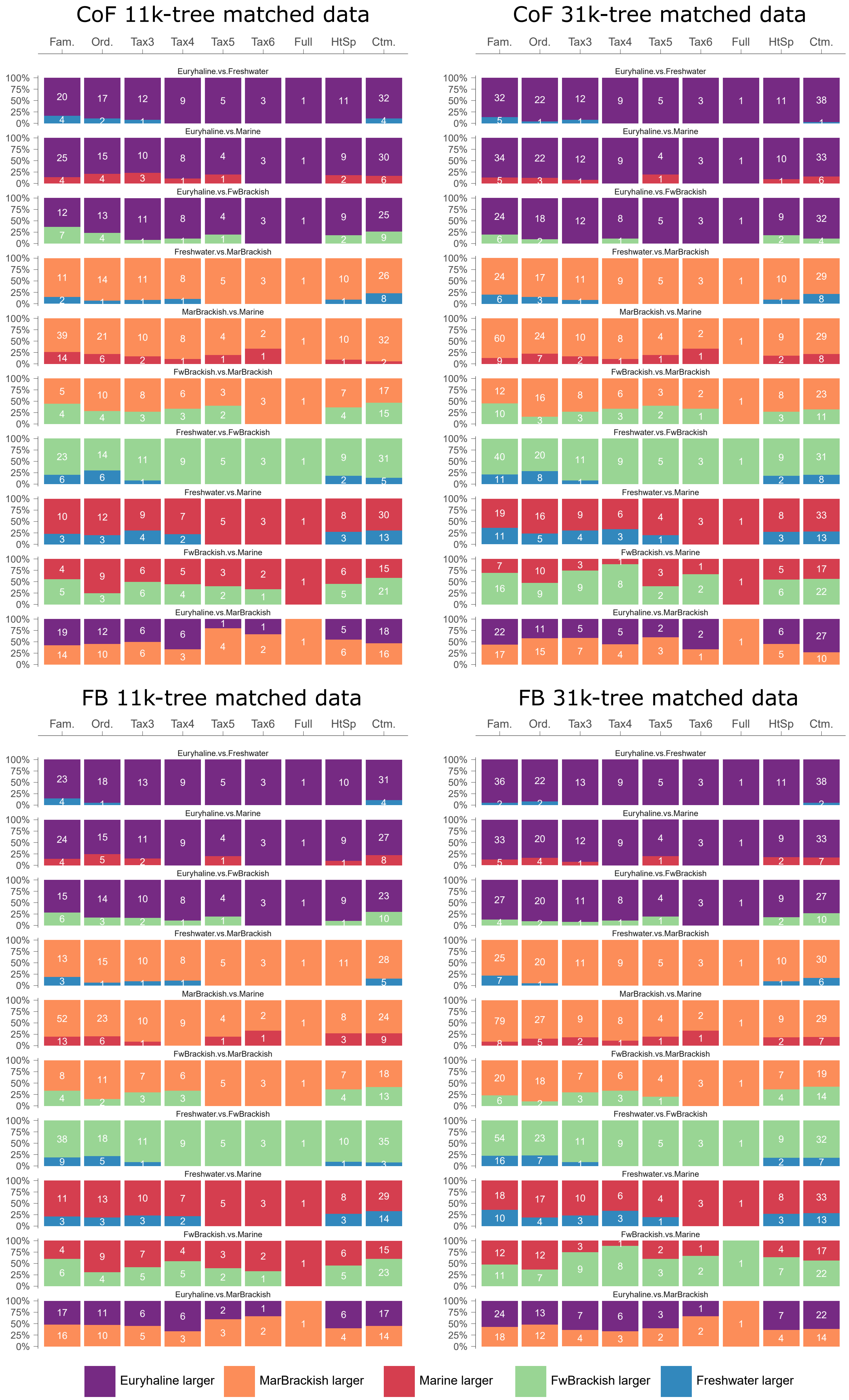


Fig. S4: The percentage of groups where the observed log10 mean size of taxa for one habitat use is larger than the other, obtained for every pairwise habitat-use comparison across multiple taxonomic scales. Results are displayed for all four datasets (FB11k, CoF11k and FB31k and CoF31k tree datasets).

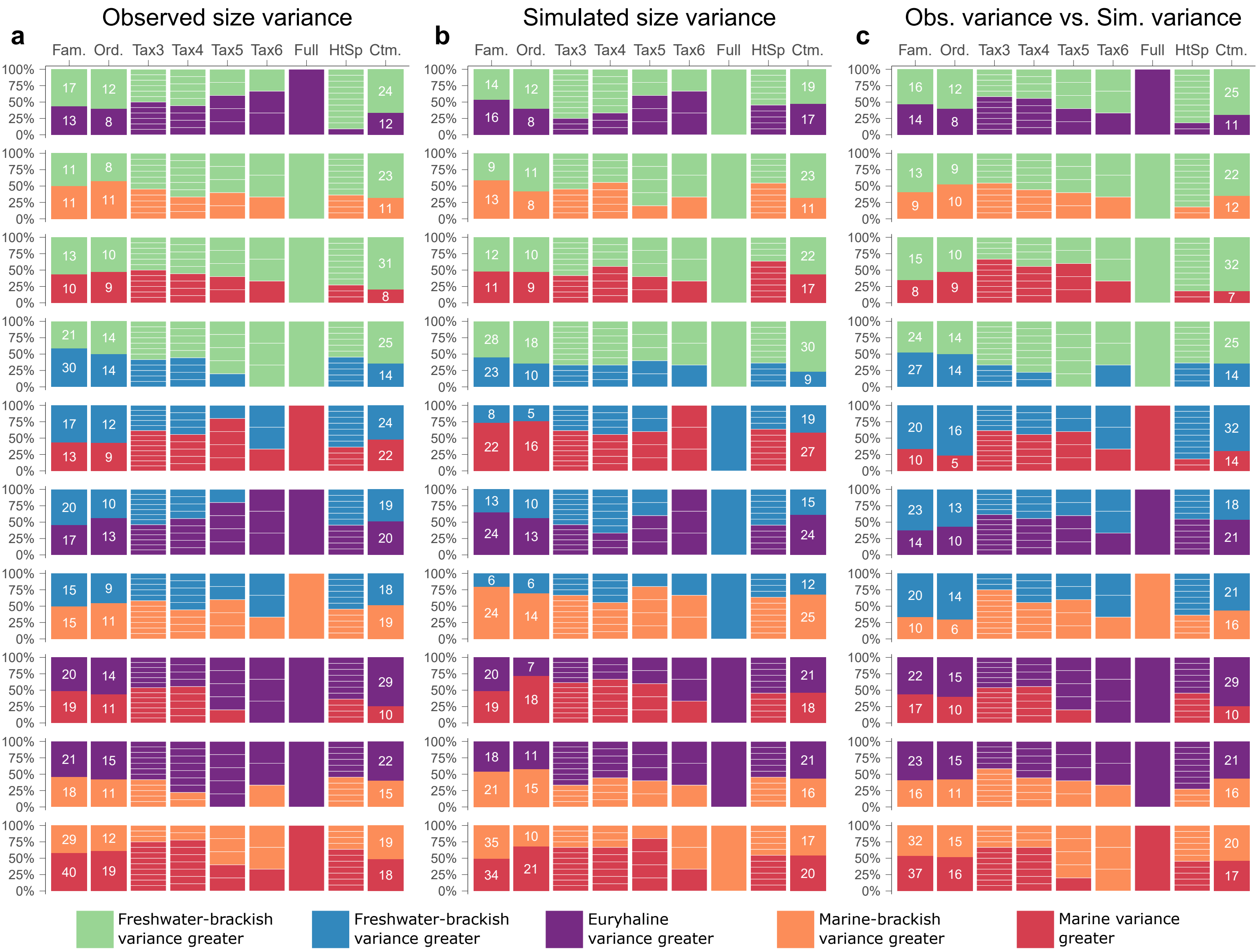


Fig. S5: The percentage of groups where the size variance of taxa within one habitat-use category is greater than the other, obtained for every pairwise habitat-use comparison across multiple taxonomic scales. (a) Size variance is that calculated from log10 size data from extant taxa without use of phylogeny. (b) Size variance represents the mean variance calculated from tip values in each habitat from 1000 simulations, thus capturing which habitat-use is expected to possess greatest size variance given the tree structure. (c) Reveals which habitat contains greater variance by comparing the ratio of observed variances to the 1000 ratios of simulated variances (see SI Methods).For definitions of taxonomic scales, see SI Methods. Individual clade segments are removed for Fam. Ord. and Ctm. taxonomic scales as they contain too many clades to individually visualise. HtSp=evolutionary hotspots. Ctm=customised expanded hotspots. Data from CoF31k tree dataset.

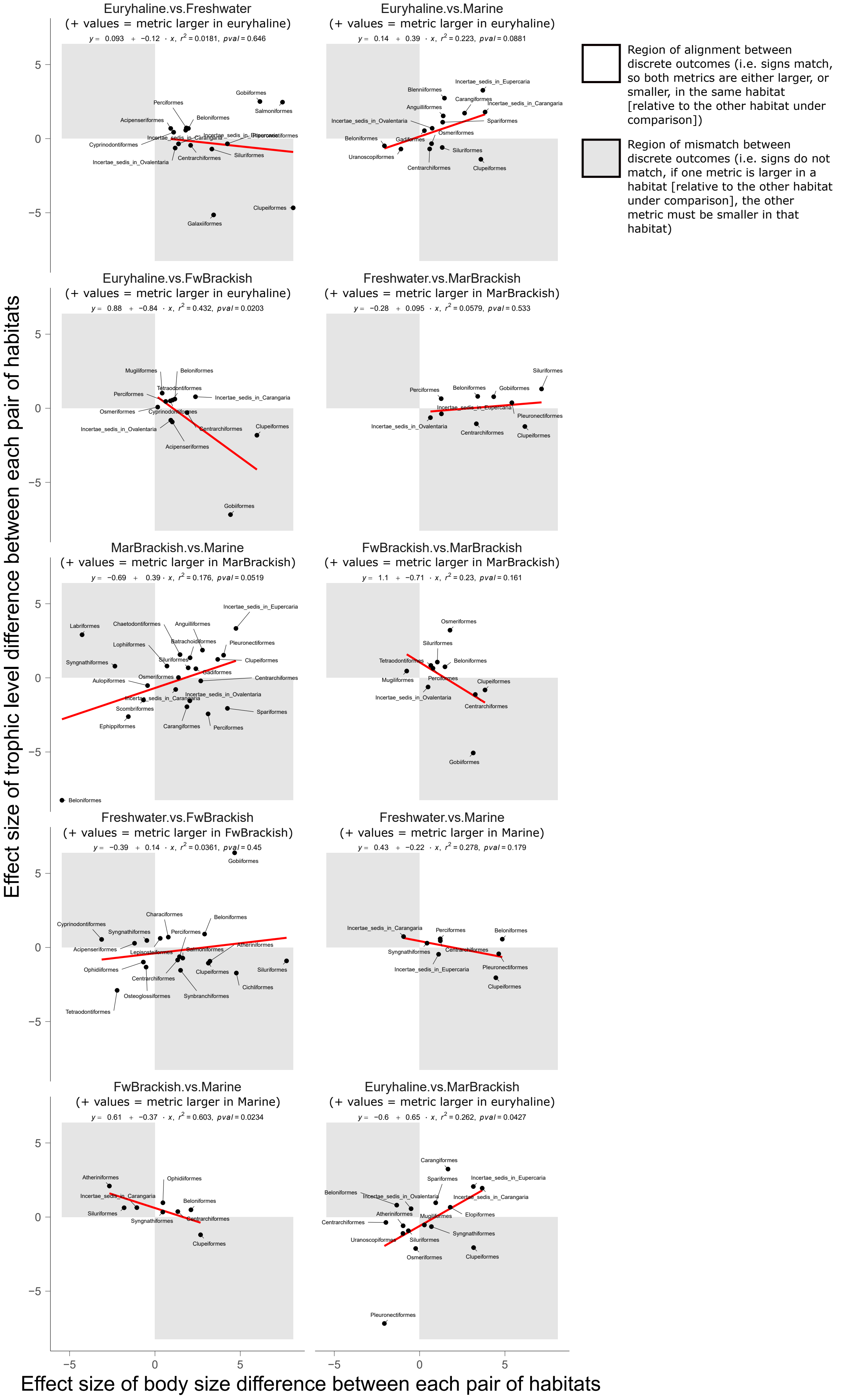


Fig. S6: The relationship between the magnitude of body size difference between two habitats (measured as phylogenetic effect size) and the magnitude of trophic level difference between two habitats (measured as phylogenetic effect size) for all ten pairwise habitat comparisons conducted in the study. Difference values are given a sign (+ or -) in order to indicate which habitat possessed the larger value. Consult the legend of Figure 4 for a detailed explanation. Where the x or y value is positive for a clade, it means the value for that axis is larger in the habitat stated in the plot's subheading as positive relative to the other habitat under comparison. Thus, for any two metrics, their outcomes can be said to align/agree when a clade plots in a white quadrant – be it the top right quadrant or the bottom left quadrant, because both metrics are larger, or smaller, in the same habitat (relative to the other habitat). Conversely, a clade falling within a grey region indicates a mismatch between metrics (e.g. one metric is larger, and the other metric is smaller, in the same habitat (relative to another habitat).

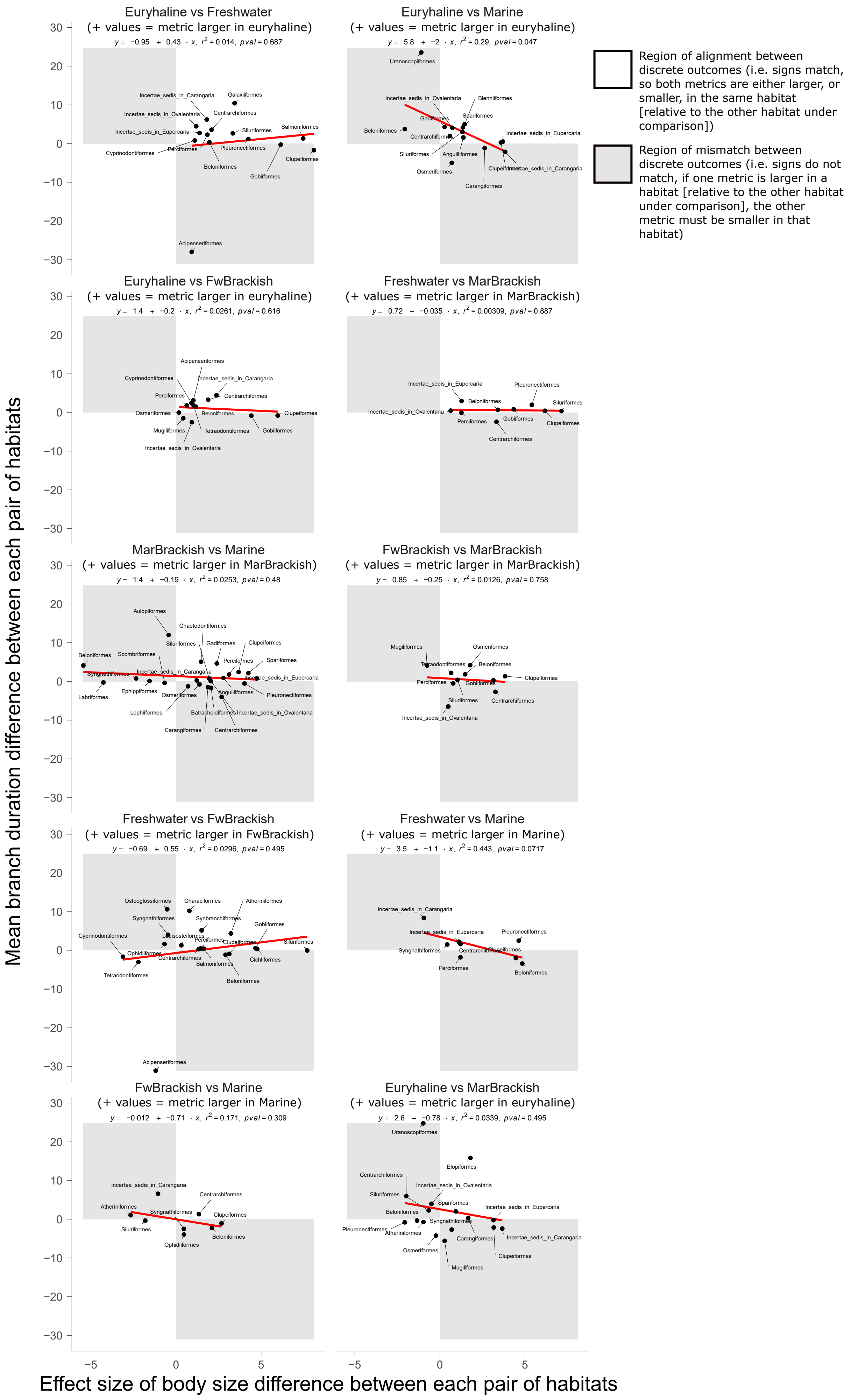


Fig. S7: The relationship between the magnitude of body size difference between two habitats (measured as phylogenetic effect size) and the magnitude of mean branch length duration difference between two habitats for all ten pairwise habitat comparisons conducted in the study. Difference values are given a sign (+ or -) in order to indicate which habitat possessed the larger value. Consult the legend of Figure 4 for a detailed explanation. Where the x or y value is positive for a clade, it means the value for that axis is larger in the habitat stated in the plot's subheading as positive relative to the other habitat under comparison. Thus, for any two metrics, their outcomes can be said to align/agree when a clade plots in a white quadrant – be it the top right quadrant or the bottom left quadrant, because both metrics are larger, or smaller, in the same habitat (relative to the other habitat). Conversely, a clade falling within a grey region indicates a mismatch between metrics (e.g. one metric is larger, and the other metric is smaller, in the same habitat (relative to another habitat).

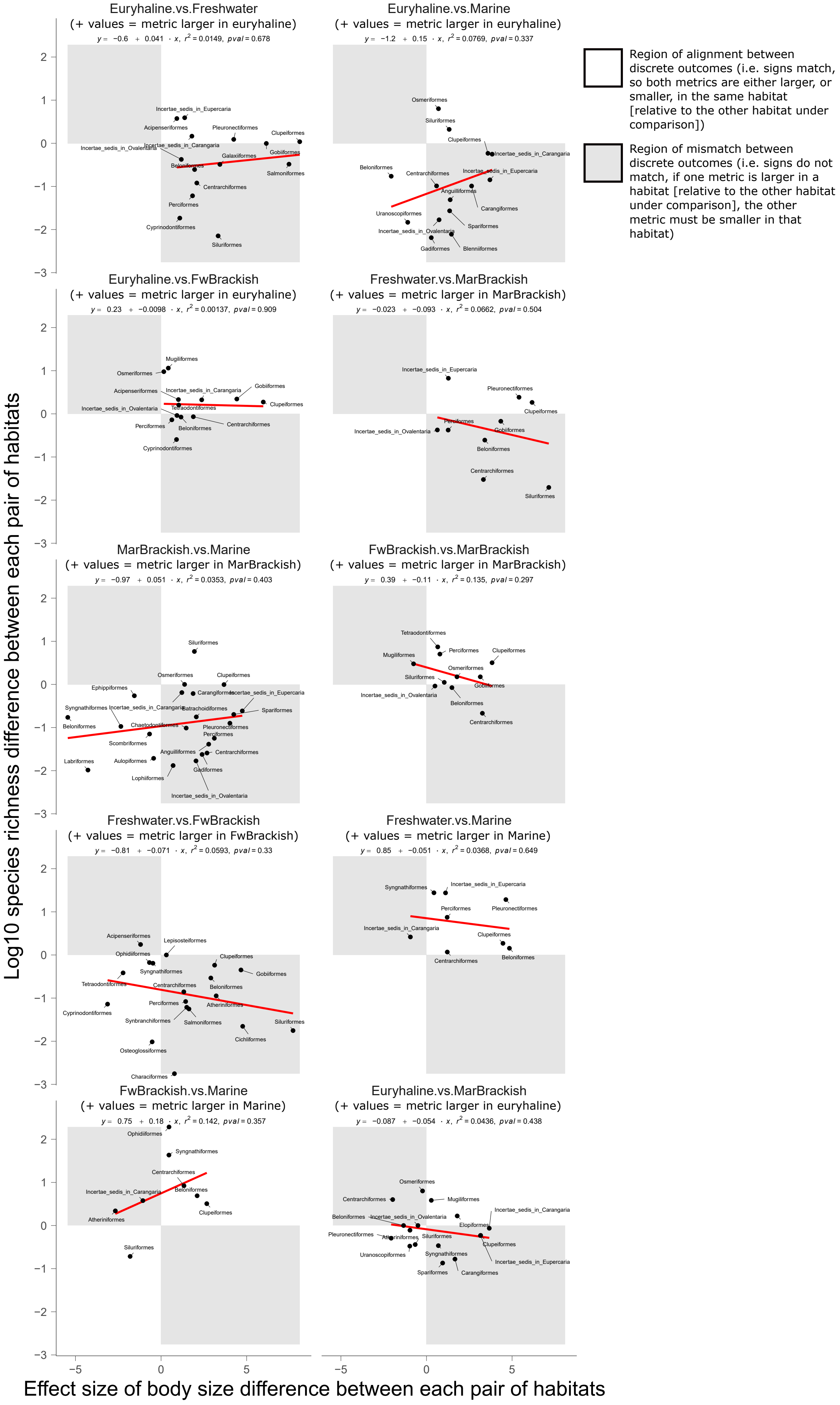


Fig. S8: The relationship between the magnitude of body size difference between two habitats (measured as phylogenetic effect size) and the magnitude of log10 richness difference between two habitats for all ten pairwise habitat comparisons conducted in the study. Difference values are given a sign (+ or -) in order to indicate which habitat possessed the larger value. Consult the legend of Figure 4 for a detailed explanation. Where the x or y value is positive for a clade, it means the value for that axis is larger in the habitat stated in the plot's subheading as positive relative to the other habitat under comparison. Thus, for any two metrics, their outcomes can be said to align/agree when a clade plots in a white quadrant – be it the top right quadrant or the bottom left quadrant, because both metrics are larger, or smaller, in the same habitat (relative to the other habitat). Conversely, a clade falling within a grey region indicates a mismatch between metrics (e.g. one metric is larger, and the other metric is smaller, in the same habitat (relative to another habitat).

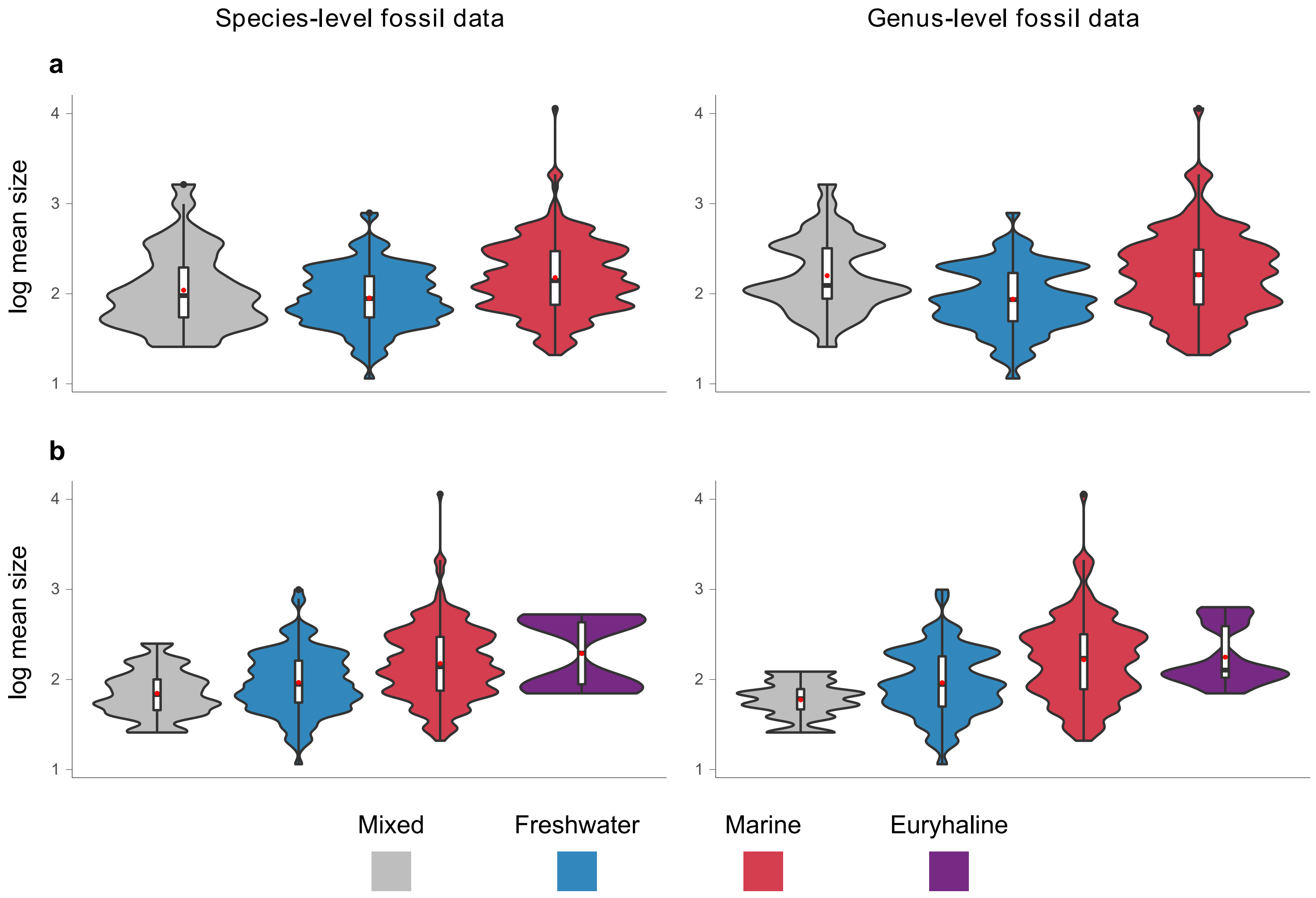


Fig. S9: Size distributions (log scale) for fossil taxa in each fossil habitat type, using mean species size data available in Clarke et al. 2016 and locality data in Clarke & Friedman 2018. Genus-level data uses the species with the largest mean size as the representative of each genus, so as to permit direct comparison with data presented in Guinot & Cavin 2018. (a) species and genera are assigned to fossil habitats in an identical manner to Guinot & Cavin 2018, where only taxa exclusive to either freshwater or marine settings are assigned to these strict categories, and all other combinations (e.g. a species occurring both in a freshwater deposit and a separate mixed deposit [mixed = a sedimentary deposit with marine and freshwater influence], or perhaps exclusively from mixed salinity deposits, are assigned to the mixed habitat type). (b) species and genera are assigned to fossil habitats in a different manner, where the assignment of a species/genus to marine or freshwater habitat type need not rely on the exclusion of that species/genus from mixed settings (e.g. its presence in freshwater and mixed deposits permits inclusion within the freshwater category, so long as it is absent from marine). This scheme acts to slightly reduce the number of species/genera coded as from mixed habitats. In addition, a species/genus found in both marine and freshwater deposits is coded as euryhaline, as is the case for extant taxa in the main text.

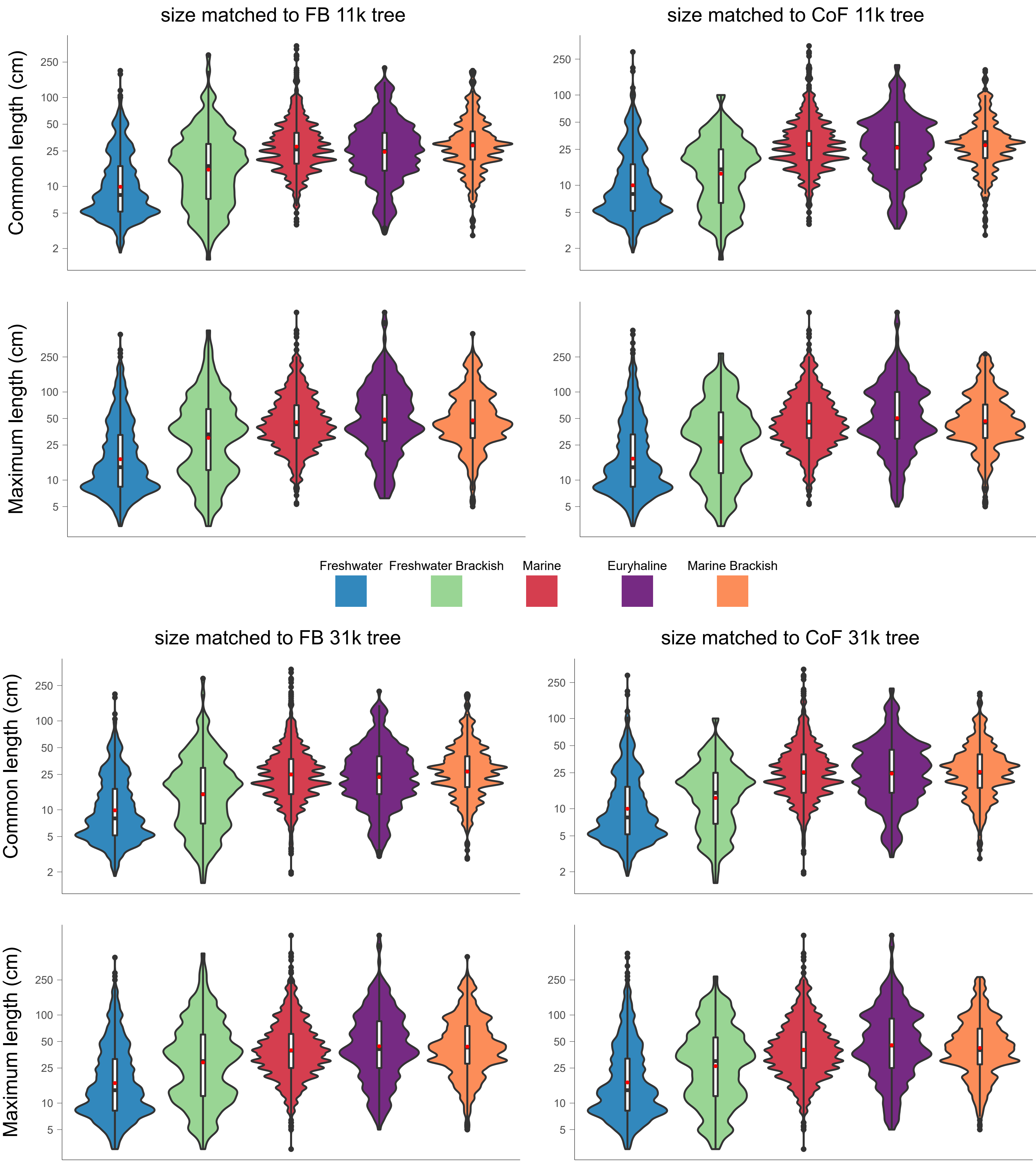


Fig. S10: Size distributions (log10 scale) of taxa with maximum length and common length measures in each habitat-use across eight datasets. See SI text for details on how these datasets were derived.

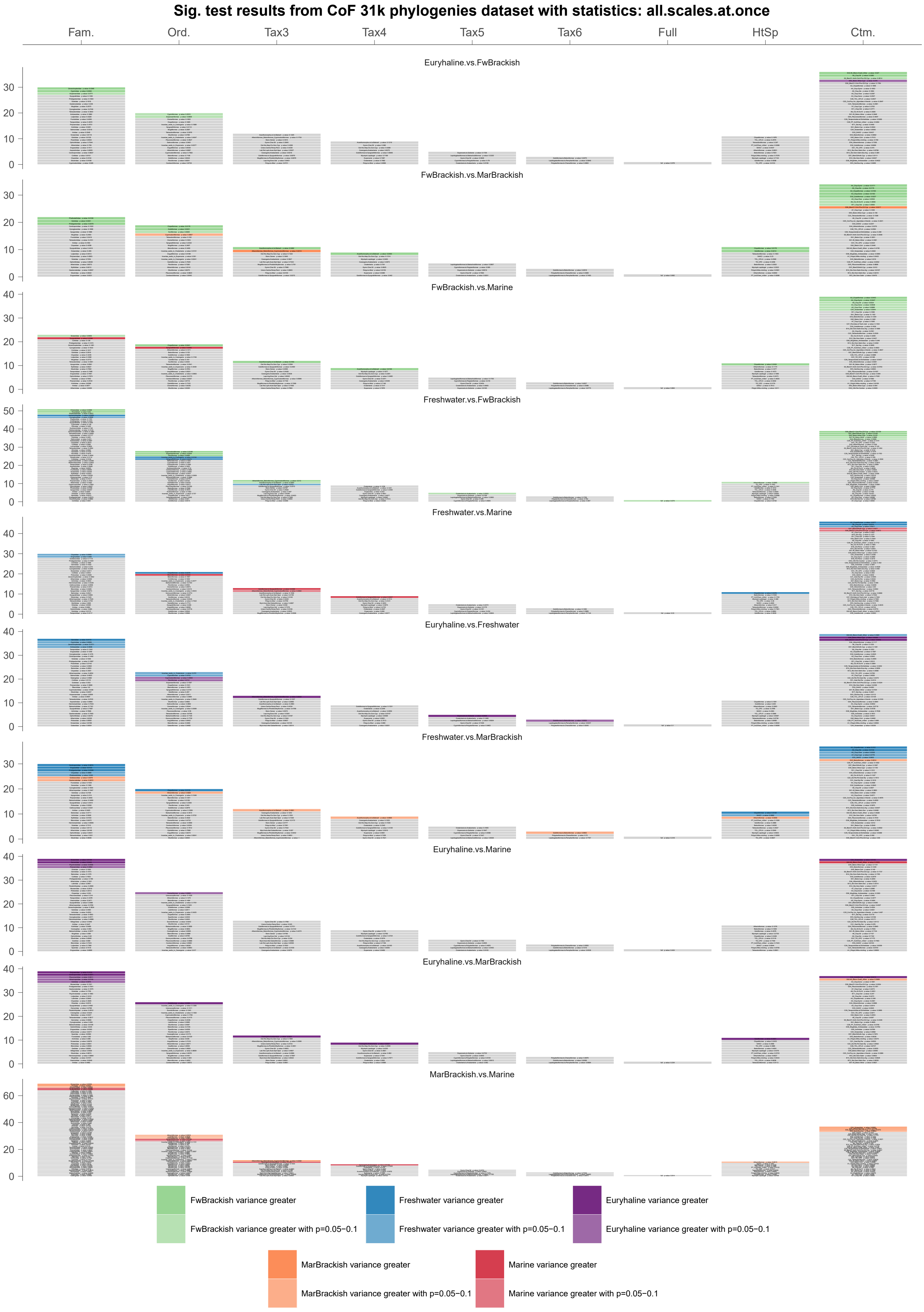


Fig. S11: The corresponding statistical values and clade information for Fig. S5c. For each pairwise habitat-use comparison across multiple taxonomic scales, this indicates the number of times each habitat-use possesses taxa with the largest size variance (relative to simulations) at probabilities of < 0.1 and < 0.05. Dark shades of each colour represent p < 0.05, lighter shades p = 0.1–0.05, and grey p > 0.1. Data from CoF31k tree dataset.

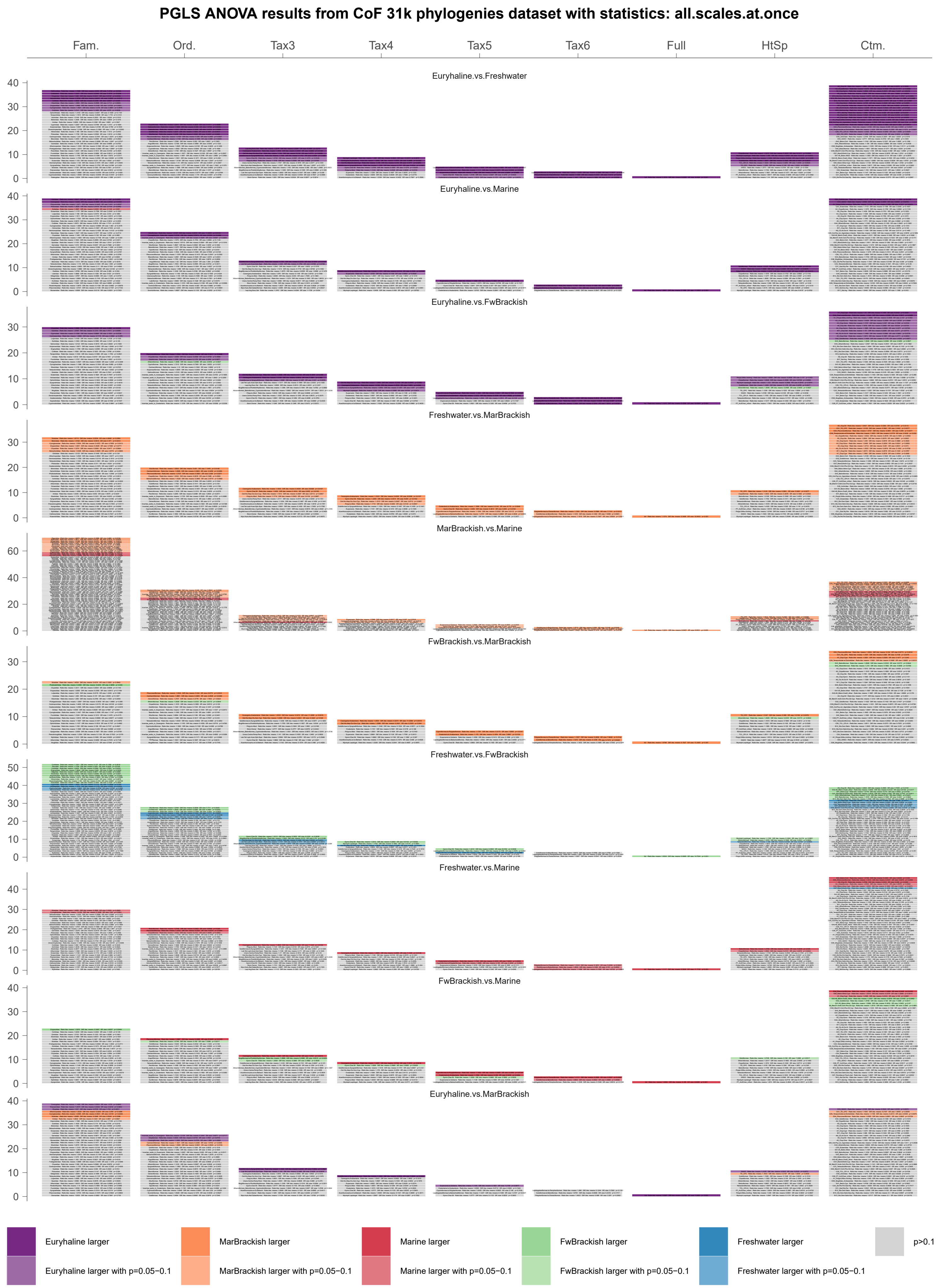


Fig. S12: The corresponding statistical values and clade information for Fig. S13b. For each pairwise habitat-use comparison across multiple taxonomic scales, this indicates the number of times each habitat use possesses taxa with the larger phylogenetic mean size at probabilities of < 0.1 and < 0.05 using PGLS ANOVA. Dark shades of each colour represent p < 0.05, lighter shades p = 0.1–0.05, and grey p > 0.1. Data from CoF31k tree dataset.

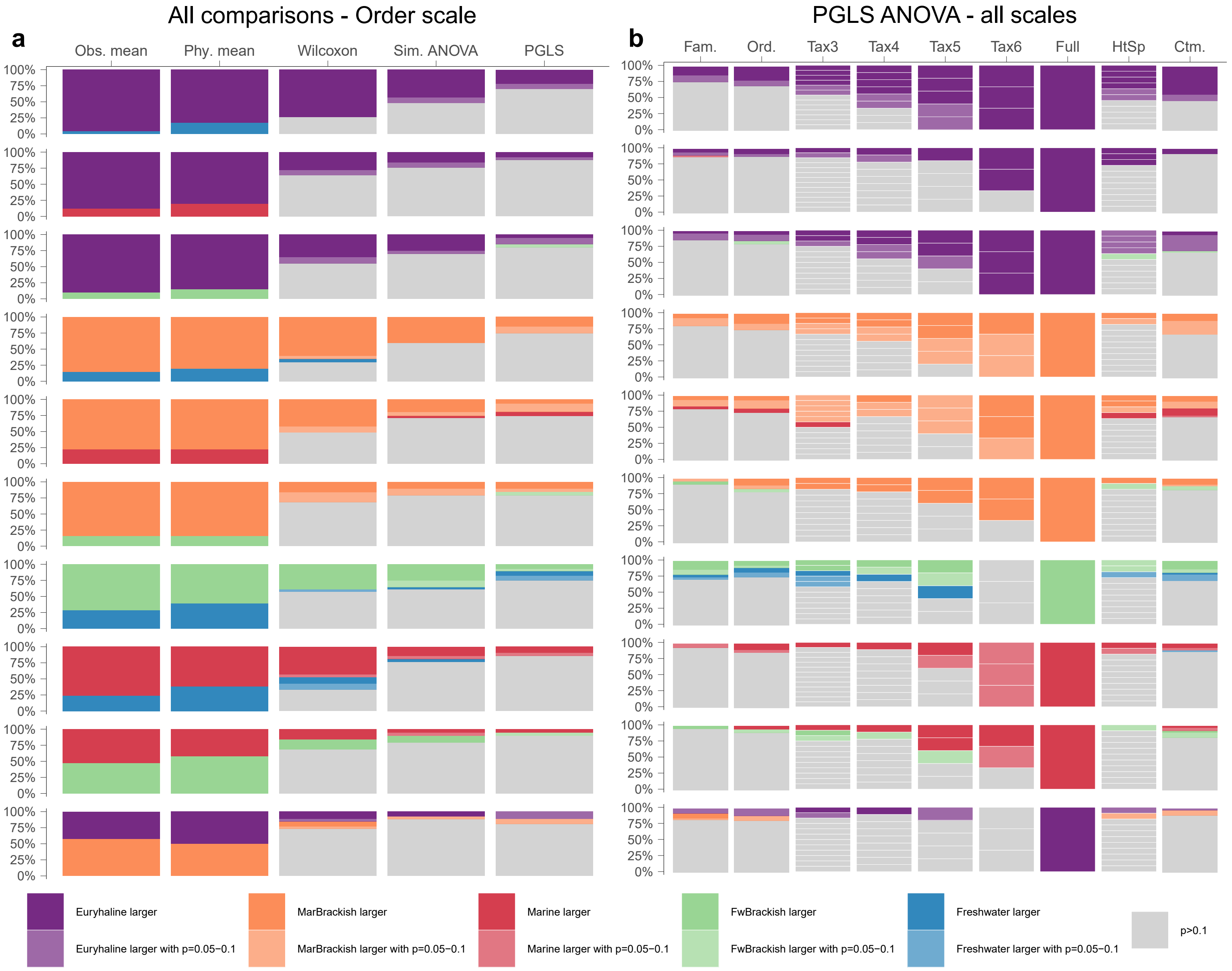


Fig. S13: (a) For every pairwise habitat-use comparison at the taxonomic scale of order, this indicates the percentage of orders in which raw and phylogenetic means are larger for one habitat-use than the other, and whether any of these size differences occurred with probabilities of < 0.1 or < 0.05 according to three statistical tests. (b) For each pairwise habitat-use comparison across multiple taxonomic scales, this indicates the number of times each habitat-use possesses taxa with the larger phylogenetic mean size at probabilities of < 0.1 and < 0.05 using PGLS ANOVA. Dark shades of each colour represent p < 0.05, lighter shades p = 0.1–0.05, and grey p > 0.1. For definitions of taxonomic scales, see methods. Individual clade segments are removed for Fam. Ord. and Ctm. taxonomic scales as they contain too many clades to individually visualise. HtSp=evolutionary hotspots. Ctm=customised expanded hotspots. Data from CoF31k tree dataset.

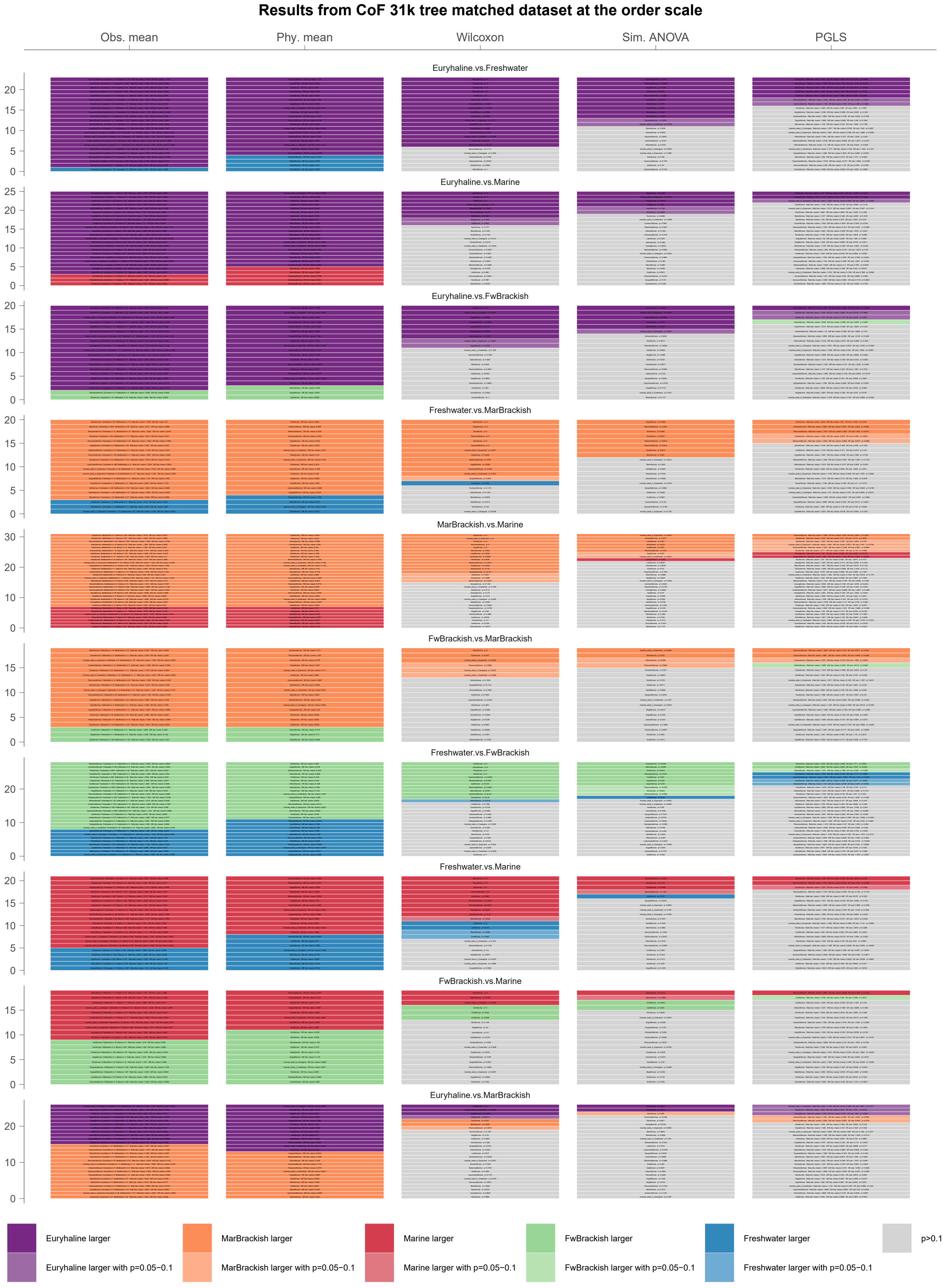


Fig. S14: The corresponding statistical values and clade information for Fig. S13a. For every pairwise habitat-use comparison at the taxonomic scale of order, this indicates the number of orders in which raw and phylogenetic means are larger for one habitat-use than the other, and whether any of these size differences occurred with probabilities of < 0.1 or < 0.05 according to three statistical tests. Dark shades of each colour represent p < 0.05, lighter shades p = 0.1–0.05, and grey p > 0.1. Data from CoF31k tree dataset.

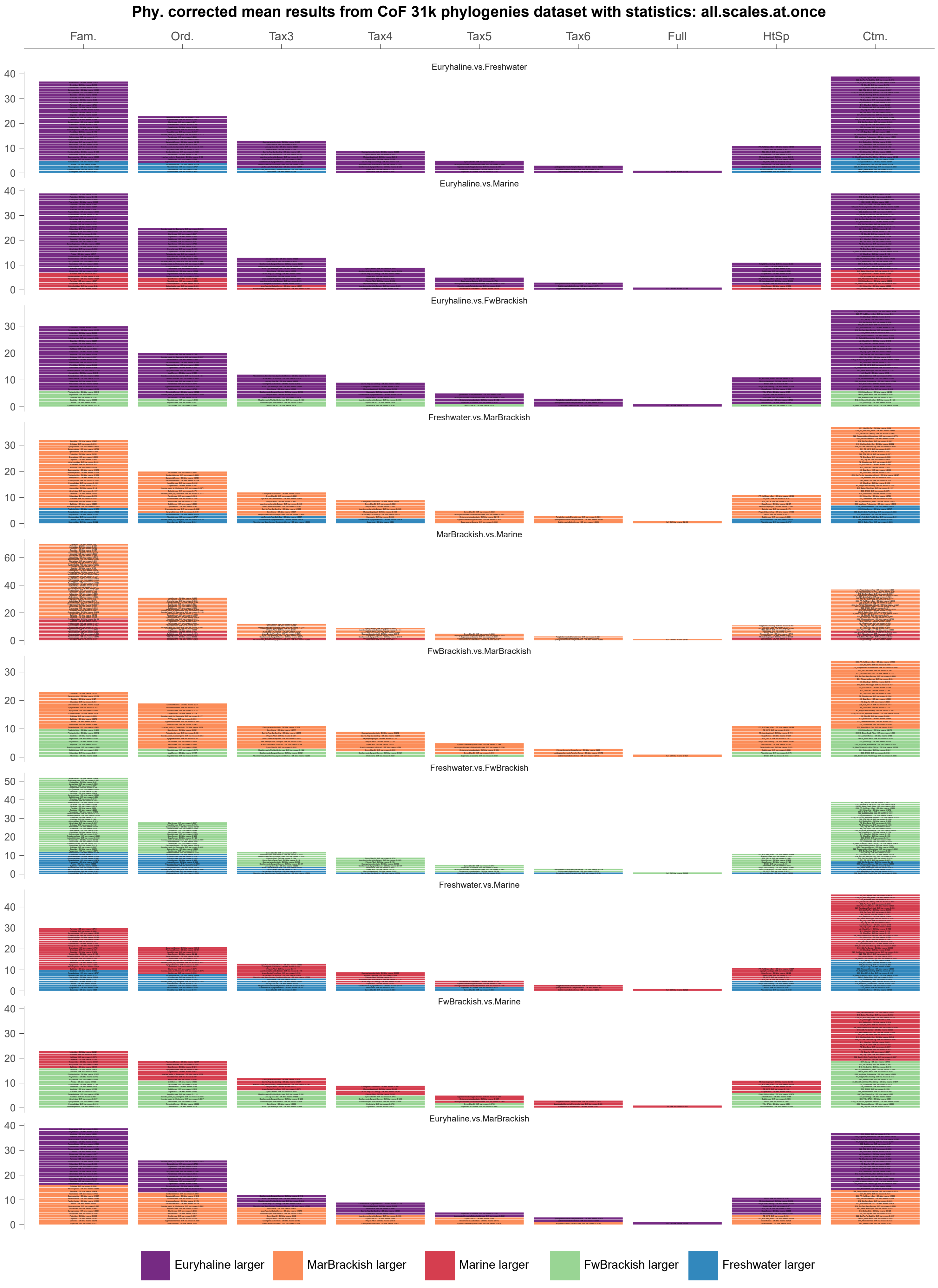


Fig. S15: The corresponding statistical values and clade information for Fig. 2a. The numbers of groups where the phylogenetic mean size (obtained from R package RRPP) of taxa for one habitat-use is larger than the other, obtained for every pairwise habitat-use comparison across multiple taxonomic scales. Data from CoF31k tree dataset.
