# Supplementary material for: Evidence for general size‐by‐habitat rules in actinopterygian fishes across nine scales of observation
Source: Ecol Lett. 2021 Jun 10;24(8):1569–81. doi: 10.1111/ele.13768 (PMC8362132; doi:10.1111/ele.13768)
Supplement: Supplementary file 2 — Table S1‐S4 [file ELE-24-1569-s018.pdf]

Table S1: List of mechanisms discussed in the main text that are proposed to explain the size-by-habitat patterns.

**MECHANISMS**<br>***MECHANISMS INFLUENCING TROPHIC LEVEL***	**BRIEF DESCRIPTOR**
	For some mechanisms, additional evidence and case studies are made beyond those examples provided in the main text. *This evidence is provided below the main description of each mechanism in grey and italicised.*
1. FOOD WEB STRUCTURE	Dominance of plankton at the food-web base in marine-influenced habitats encourages additional trophic levels to form<br><br>*e.g. a decrease in the number of herbivore and detritivore individuals in creeks and rivers compared to the ocean in the Tortuguero region, Costa Rica, is consistent with how changes in the food web base influence the trophic structure of fish assemblages between marine and freshwater (Winemiller & Leslie, 1992).*
2. SENSITIVITY TO HIGHER ENERGY DEMANDS IN WARM WATERS	Freshwater taxa appear more sensitive to higher energy demands of warm waters and feed at lower trophic levels relative to warm water marine taxa
3. ECOSYSTEM SIZE	Larger settings, typically more representative of marine habitats, encourage additional trophic levels to form
4. ECOSYSTEM STABILITY	Freshwater taxa are less able to respond to habitat fragmentation and temperature variability, reducing the number of trophic levels than can form
5. ECOSYSTEM AGE	Marine environments are typically older, fostering complexity that increases the number of trophic levels
6. TOPOLOGICAL VARIETY, PARTICULARLY DEPTH	Greater topological variation (particularly regarding depth) of marine-influenced settings encourages additional trophic levels
7. DEGREE OF OMNIVORY (DEFINED AS TROPHIC DIETARY VARIETY)	Higher degree of omnivory (when defined as the variety of trophic levels consumed) in marine-influenced settings sustains additional trophic levels<br><br>*Clades that act as case studies demonstrating this pattern are Scorpaeniformes, Siluriformes, and Ariidae (Sanchez-Hernandez & Amundsen 2018). The pattern also emerges within specific global regions and feeding strategies (Sanchez-Hernandez & Amundsen 2018).*
In addition to evidence for individual mechanisms, there are literature case studies demonstrating higher trophic levels in marine settings relative to freshwater settings. This has been demonstrated within Osmeriformes, Scorpaeniformes, Siluriformes and Ariidae. The pattern also emerges within specific global regions and feeding strategies (Sanchez-Hernandez & Amundsen 2018).	
The seven mechanisms above are predicted to frequently promote larger mean trophic level (and by association size) in various marine-influenced settings over freshwater. This alignment may help to explain the consistency of the size patterns, because even if mechanisms do not interact, the existence of several mechanisms means that, for a given clade, at least one mechanism should be active.<br><br>In other clades, several mechanisms may be active simultaneously, which may act to outnumber any selective forces that drive larger sizes in freshwater taxa. Nevertheless, it should be expected that a variety of habitat types could strongly exploit the above mechanisms under specific circumstances (i.e. not only that marine settings should always contain longer trophic chains than a given freshwater setting). For instance, a geologically old, deep lake may be expected to have developed more trophic levels than a young marine lagoon. Therefore, it is important to record all instances of agreement between size and trophic patterns across every individual habitat comparison when considering the role of trophic mechanisms.<br><br>*Table S6 lists all comparisons where the trophic outcome is consistent with the size outcome. This information is summarised in Tables 1 and S5. Figure S6 shows how the phylogenetic effect size of size differences between two habitats corresponds to the phylogenetic effect size of trophic-level differences between those two habitats.*	

ALTERNATIVE MECHANISMS	
8. MIGRATORY LIFESTYLES	Numerous migrators in the euryhaline category. Benefits of large size for migration<br><br>*Likely these selective forces are decisive in instances of size–trophic mismatch where the habitat with the larger taxa also contains a notably higher percentage of migrators than the other habitat under comparison (indicated as "Migration strong" in Tables 1, S5, S6). Orders demonstrating this clearly include Galaxiiformes and Clupeiformes in euryhaline vs. freshwater comparisons; Centrarchiformes, Clupeiformes, Gobiiformes, I.S. Ovalentaria in euryhaline vs. freshwater-brackish comparisons; Clupeiformes and Mugiliformes in euryhaline vs. marine-brackish comparisons; Clupeiformes, Centrarchiformes and Osmeriformes in euryhaline vs. marine comparisons; Perciformes in freshwater vs. freshwater-brackish comparisons (Table S6, Figure S6).*
9. LINEAGE AGE	Taxa may be larger due to the presence of evolutionary trends towards large size (e.g. Depéret's / Cope's Rule) or from the effects of ecosystem age on trophic level, noted above. It has also been suggested that in some instances, freshwater environments act as refugia that encourage the development of geologically old and large taxa known as 'living fossils'<br><br>*Figure 1b illustrates this 'large living fossil' idea well, showing how freshwater taxa for classic 'living fossil' clades such as Acipenseriformes and Lepisosteiformes are atypically large, even in comparison to many marine or euryhaline clades*<br><br>*Table S6 lists all comparisons where the greater lineage age corresponds with larger size. This information is summarised in Tables 1 and S5.*
10. CLADE SPECIES RICHNESS	Ecological limits on the numbers of very large species (occupying high trophic levels) that can be sustained (i.e. food webs contain few very high trophic level species relative to small and medium sized species). Thus, there should generally be more diversification opportunities at lower trophic levels than at high trophic levels, meaning it should be easier for a species poor clade to display a larger mean size relative to a species rich clade.<br>(e.g. Tables 1, S5, S6).<br><br>*Table S6 lists all comparisons where lower species richness corresponds with larger size. This information is summarised in Tables 1 and S5.*
11. HABITAT COMPLEXITY	Freshwater settings may demand greater manoeuvrability, and encourage smaller size as a result
12. STILL WATERS	Still waters, common in freshwater settings, may permit / encourage small size<br><br>*Weitzman and Vari 1988 highlight case studies in the form of South American Characiformes (Lebiasinidae, Characidae), Siluriformes (Trichomycteridae, Loricariidae, Callichthyiidae, Aspredinidae, Scoloplacidae, Pimelodidae), Cyprinodontiformes (Poeciliidae, Poeciliinae), Clupeiformes (Engraulidae) and Perciformes (Eleotrididae).*
13. ANTI-PREDATION STRATEGIES	Larger size to evade or escape predators. Selection pressures potentially more prevalent in marine-influenced habitats where predatory taxa are more common for trophic reasons (e.g. plankton dominated food-web base).
14. PREDATION STRATEGIES	Benefits of large size to hunting. Expected to be more prevalent where higher trophic levels are encouraged, and where piscivorous lifestyles are more common due to a plankton dominated food-web base, as seen in numerous marine-influenced settings

Table S2: The % of clades (Tax3 scale) in which each pair of metrics, from the nine metrics compared between habitats, were aligned. For example, if comparing size and richness outcomes for euryhaline vs. freshwater comparisons (top row, red highlight), the % of alignments will equal the % of clades in which, relative to the total number of Tax3 clades in which the two habitat types could be compared, euryhaline taxa possessed either i) the smaller mean size and lower species richness, or ii) the larger mean size and higher species richness. Cumulatively, these two outcomes occurred in 18.2% of comparisons. I commonly refer to these as 'percentage alignments' of discrete outcomes. Clades whose metrics are aligned for a given habitat comparison fall within the white quadrants of Figure 4, while mismatched outcomes fall within grey quadrants. **Only those 'percentage alignments' that were either consistently high or consistently low across multiple datasets are shown here; Appendix 1 contains percentages for all pairwise metric comparisons.** Colour coding is applied to pairs of metrics that achieve high or low % alignments in more than one set of pairwise habitat comparisons. Table S6 takes these alignments and converts them into support for or against particular mechanisms. For example, depending on the mechanism, an alignment may deliver support (e.g. where trophic level and size outcomes agree), or a mismatch may deliver support (e.g. the clade with larger taxa should possess the lower species richness if ecological limits leave a strong signature on clades). Note: migratory %'s are often <u>identical</u> (i.e. in the many comparisons where no migratory species are present). This inflates the number of mismatches when comparing migratory %'s to other traits (hence commonly low alignment %'s in Appendix 1 in comparisons involving migratory %) because unless the other trait under comparison is also identical, it will be recorded as a mismatch. As such, these trait alignment %'s are omitted from this summary Table S2. Instead, Tables S4, S5 and S6 more clearly highlight the comparisons where migrators are present in sufficient numbers, and the significance of these outcomes regarding support for theories underpinning size patterns.

	**highly positively associated outcomes**					**low percentage of aligned outcomes**
**Euryhaline vs. Freshwater**			Troph var rat. vs.<br>$\bar{X}$ Size<br>63.6	$\bar{X}$ Phy Troph vs.<br>$\bar{X}$ Bdur<br>27.3	**Troph var rat. vs.<br>richness<br>18.2**	**$\bar{X}$ Phy size vs.<br>richness<br>18.2**
**Euryhaline vs. Marine**					**$\bar{X}$ Phy size vs.<br>richness<br>27.3**	
**Euryhaline vs. FwBrackish**					**Troph var rat. vs.<br>richness<br>25**	
**Freshwater vs. MarBrackish**	**$\bar{X}$ Troph vs.<br>$\bar{X}$ Size<br>100**				**$\bar{X}$ Phy size vs.<br>richness<br>25**	$\bar{X}$ Bdur vs.<br>richness<br>12.5
**MarBrackish vs. Marine**	**$\bar{X}$ Phy Troph vs.<br>$\bar{X}$ Phy size<br>100**		**$\bar{X}$ Troph vs.<br>$\bar{X}$ Size<br>72.7**		**$\bar{X}$ Phy size vs.<br>richness<br>27.3**	
**FwBrackish vs. MarBrackish**			**$\bar{X}$ Phy size vs.<br>richness<br>77.8**			
**Freshwater vs. FwBrackish**	**$\bar{X}$ Troph vs.<br>$\bar{X}$ Size<br>75**	$\bar{X}$ Phy size vs.<br>Size var rat.<br>75	Size var rat. vs.<br>$\bar{X}$ Bdur<br>62.5	$\bar{X}$ Troph vs.<br>richness<br>25	Troph var rat. vs.<br>$\bar{X}$ Bdur<br>25	**$\bar{X}$ Phy size vs.<br>richness<br>12.5**
**Freshwater vs. Marine**	**Troph var rat. vs.<br>richness<br>77.8**	**$\bar{X}$ Phy size vs.<br>richness<br>77.8**			Size var rat. vs.<br>$\bar{X}$ Bdur<br>33.3	$\bar{X}$ Troph vs.<br>$\bar{X}$ Bdur<br>11.1
**FwBrackish vs. Marine**	$\bar{X}$ Phy Troph vs.<br>Troph var rat.<br>87.5	Troph var rat. vs.<br>$\bar{X}$ Size<br>87.5	$\bar{X}$ Phy Troph vs.<br>Size var rat.<br>75		**$\bar{X}$ Troph vs.<br>Troph var rat.<br>37.5**	**Troph var rat. vs.<br>richness<br>25**
**Euryhaline vs. MarBrackish**	**$\bar{X}$ Phy Troph vs.<br>$\bar{X}$ Phy size<br>88.9**		$\bar{X}$ Troph vs.<br>Size var rat.<br>77.8	Troph var rat. vs.<br>$\bar{X}$ Bdur<br>44.4	Size var rat. vs.<br>richness<br>33.3	**$\bar{X}$ Troph vs.<br>Troph var rat.<br>22.2**

$\bar{X}$ Troph = Mean log10 trophic level

$\bar{X}$ Phy Troph = Mean log10 phylogenetic trophic level

$\bar{X}$ Size = Mean log10 body size

$\bar{X}$ Phy size = Mean log10 phylogenetic body size

$\bar{X}$ Bdur = Mean branch duration

Size var rat. = Observed size variance ratio relative to 1000 simulated variance ratios

Troph var rat. = Observed trophic level variance ratio relative to 1000 simulated variance ratios

richness = species richness

Table S3: Numbers and percentages of migratory taxa, from the list of migratory species recently defined and compiled in Burns & Bloom (2020), within each habitat-use type across the four datasets. Illustrates relatively high percentages of migratory taxa within the euryhaline category.

FishBase 11k tree matched dataset

	Freshwater	Freshwater brackish	Marine	Marine brackish	Euryhaline
Sp. in habitat	4938	418	4335	741	456
No. of Migratory Sp.	187	57	70	35	230
% Migrators	3.8	13.6	1.6	4.7	50.4

Catalogue of Fishes 11k tree matched dataset

	Freshwater	Freshwater brackish	Marine	Marine brackish	Euryhaline
Sp. in habitat	4529	318	4236	611	490
No. of Migratory Sp.	182	42	82	26	210
% Migrators	4.02	13.21	1.94	4.26	42.86

FishBase 31k tree matched dataset

	Freshwater	Freshwater brackish	Marine	Marine brackish	Euryhaline
Sp. in habitat	12890	721	11597	1248	700
No. of Migratory Sp.	188	58	70	36	232
% Migrators	1.46	8.04	0.60	2.88	33.14

Catalogue of Fishes 31k tree matched dataset

	Freshwater	Freshwater brackish	Marine	Marine brackish	Euryhaline
Sp. in habitat	11392	566	11282	1052	760
No. of Migratory Sp.	182	43	82	26	211
% Migrators	1.60	7.60	0.73	2.47	27.76

Table S4: Numbers and percentages of migratory taxa in every order and habitat subdivision for the CoF 31k-tree dataset. List of migratory species defined and compiled in Burns & Bloom (2020).

Order and Habitat	No. species in habitat	No. of migratory species	% migrators
Acanthuriformes_Marine	83	0	0
Acipenseriformes_Euryhaline	15	14	93.33333333
Acipenseriformes_Freshwater	4	4	100
Acipenseriformes_Freshwater brackish	7	6	85.71428571
Albuliformes_Marine	6	1	16.66666667
Albuliformes_Marine brackish	4	0	0
Alepocephaliformes_Marine	118	0	0
Amiiformes_Freshwater	1	1	100
Anabantiformes_Freshwater	210	2	0.952380952
Anabantiformes_Freshwater brackish	5	0	0
Anguilliformes_Euryhaline	31	14	45.16129032
Anguilliformes_Freshwater	4	0	0
Anguilliformes_Freshwater brackish	3	0	0
Anguilliformes_Marine	632	3	0.474683544
Anguilliformes_Marine brackish	26	0	0
Argentiniformes_Marine	72	0	0
Ateleopodiformes_Marine	7	0	0
Atheriniformes_Brackish	3	0	0
Atheriniformes_Euryhaline	21	0	0
Atheriniformes_Freshwater	142	0	0
Atheriniformes_Freshwater brackish	16	0	0
Atheriniformes_Marine	35	0	0
Atheriniformes_Marine brackish	27	0	0
Aulopiformes_Marine	207	1	0.483091787
Aulopiformes_Marine brackish	4	0	0
Batrachoidiformes_Euryhaline	4	0	0
Batrachoidiformes_Freshwater	5	0	0
Batrachoidiformes_Marine	51	0	0
Batrachoidiformes_Marine brackish	9	0	0
Beloniformes_Euryhaline	16	0	0
Beloniformes_Freshwater	65	0	0
Beloniformes_Freshwater brackish	19	0	0
Beloniformes_Marine	93	0	0
Beloniformes_Marine brackish	16	0	0
Beryciformes_Marine	135	0	0
Blenniiformes_Brackish	1	0	0
Blenniiformes_Euryhaline	7	0	0
Blenniiformes_Freshwater	10	0	0
Blenniiformes_Freshwater brackish	1	0	0
Blenniiformes_Marine	900	0	0
Blenniiformes_Marine brackish	27	0	0
Carangiformes_Euryhaline	9	2	22.22222222
Carangiformes_Marine	88	2	2.272727273
Carangiformes_Marine brackish	54	0	0
Centrarchiformes_Brackish	1	0	0
Centrarchiformes_Euryhaline	12	4	33.33333333

Centrarchiformes_Freshwater	100	15	15
Centrarchiformes_Freshwater brackish	14	1	7.142857143
Centrarchiformes_Marine	117	1	0.854700855
Centrarchiformes_Marine brackish	3	0	0
Chaetodontiformes_Euryhaline	1	0	0
Chaetodontiformes_Marine	144	0	0
Chaetodontiformes_Marine brackish	14	0	0
Characiformes_Euryhaline	1	0	0
Characiformes_Freshwater	1697	37	2.180318209
Characiformes_Freshwater brackish	3	1	33.33333333
Characiformes_Marine	1	0	0
Cichliformes_Brackish	1	0	0
Cichliformes_Euryhaline	1	0	0
Cichliformes_Freshwater	1353	0	0
Cichliformes_Freshwater brackish	30	0	0
Cichliformes_Marine	2	0	0
Clupeiformes_Brackish	5	0	0
Clupeiformes_Euryhaline	62	23	37.09677419
Clupeiformes_Freshwater	57	2	3.50877193
Clupeiformes_Freshwater brackish	33	0	0
Clupeiformes_Marine	106	1	0.943396226
Clupeiformes_Marine brackish	105	4	0.038095238
Cypriniformes_Euryhaline	3	0	0
Cypriniformes_Freshwater	2592	67	2.584876543
Cypriniformes_Freshwater brackish	108	13	12.03703704
Cyprinodontiformes_Brackish	1	0	0
Cyprinodontiformes_Euryhaline	16	0	0
Cyprinodontiformes_Freshwater	869	0	0
Cyprinodontiformes_Freshwater brackish	63	0	0
Cyprinodontiformes_Marine	1	0	0
Cyprinodontiformes_Marine brackish	5	0	0
Elopiformes_Euryhaline	5	3	60
Elopiformes_Marine	1	0	0
Elopiformes_Marine brackish	3	1	33.33333333
Ephippiformes_Euryhaline	1	0	0
Ephippiformes_Marine	11	0	0
Ephippiformes_Marine brackish	6	0	0
Esociformes_Freshwater	9	1	11.11111111
Esociformes_Freshwater brackish	1	1	100
Gadiformes_Euryhaline	3	0	0
Gadiformes_Freshwater brackish	1	1	100
Gadiformes_Marine	463	12	2.591792657
Gadiformes_Marine brackish	11	2	18.18181818
Galaxiiformes_Euryhaline	11	9	81.81818182
Galaxiiformes_Freshwater	34	0	0
Gobiiformes_Brackish	26	0	0
Gobiiformes_Euryhaline	234	64	27.35042735
Gobiiformes_Freshwater	236	2	0.847457627
Gobiiformes_Freshwater brackish	106	6	5.660377358
Gobiiformes_Marine	757	1	0.132100396
Gobiiformes_Marine brackish	159	4	2.51572327
Gonorynchiformes_Euryhaline	2	1	50
Gonorynchiformes_Freshwater	29	0	0

Gonorynchiformes_Marine	4	0	0
Gymnotiformes_Freshwater	177	0	0
Hiodontiformes_Freshwater	2	1	50
Holocentriformes_Marine	79	0	0
Holocentriformes_Marine brackish	1	0	0
Incertae_sedis_in_Carangaria_Euryhaline	19	2	10.52631579
Incertae_sedis_in_Carangaria_Freshwater	13	0	0
Incertae_sedis_in_Carangaria_Freshwater brackish	9	0	0
Incertae_sedis_in_Carangaria_Marine	34	0	0
Incertae_sedis_in_Carangaria_Marine brackish	22	0	0
Incertae_sedis_in_Eupercaria_Brackish	2	0	0
Incertae_sedis_in_Eupercaria_Euryhaline	86	5	5.813953488
Incertae_sedis_in_Eupercaria_Freshwater	22	0	0
Incertae_sedis_in_Eupercaria_Freshwater brackish	8	0	0
Incertae_sedis_in_Eupercaria_Marine	605	3	0.495867769
Incertae_sedis_in_Eupercaria_Marine brackish	147	6	4.081632653
Incertae_sedis_in_Ovalentaria_Euryhaline	11	3	27.27272727
Incertae_sedis_in_Ovalentaria_Freshwater	26	2	7.692307692
Incertae_sedis_in_Ovalentaria_Freshwater brackish	12	0	0
Incertae_sedis_in_Ovalentaria_Marine	653	0	0
Incertae_sedis_in_Ovalentaria_Marine brackish	11	0	0
Istiophoriformes_Marine	10	10	100
Kurtiformes_Euryhaline	1	0	0
Kurtiformes_Freshwater	8	0	0
Kurtiformes_Freshwater brackish	4	0	0
Kurtiformes_Marine	220	0	0
Kurtiformes_Marine brackish	5	0	0
Labriformes_Euryhaline	2	0	0
Labriformes_Marine	581	0	0
Labriformes_Marine brackish	6	0	0
Lampridiformes_Marine	21	0	0
Lepidogalaxiiformes_Freshwater	1	0	0
Lepisosteiformes_Euryhaline	1	0	0
Lepisosteiformes_Freshwater	3	0	0
Lepisosteiformes_Freshwater brackish	3	1	33.33333333
Lobotiformes_Euryhaline	1	0	0
Lobotiformes_Freshwater	3	0	0
Lobotiformes_Freshwater brackish	2	0	0
Lobotiformes_Marine	1	0	0
Lophiiformes_Marine	228	0	0
Lophiiformes_Marine brackish	3	0	0
Mugiliformes_Euryhaline	23	9	39.13043478
Mugiliformes_Freshwater	1	0	0
Mugiliformes_Freshwater brackish	2	0	0
Mugiliformes_Marine	3	0	0
Mugiliformes_Marine brackish	6	0	0
Myctophiformes_Marine	186	0	0
Notacanthiformes_Marine	20	0	0
Ophidiiformes_Freshwater	3	0	0
Ophidiiformes_Freshwater brackish	2	0	0
Ophidiiformes_Marine	386	0	0
Ophidiiformes_Marine brackish	5	0	0

Osmeriformes_Euryhaline	19	17	89.47368421
Osmeriformes_Freshwater	2	0	0
Osmeriformes_Freshwater brackish	2	2	100
Osmeriformes_Marine	3	1	33.33333333
Osmeriformes_Marine brackish	3	1	33.33333333
Osteoglossiformes_Freshwater	207	0	0
Osteoglossiformes_Freshwater brackish	2	0	0
Pempheriformes_Euryhaline	1	0	0
Pempheriformes_Marine	141	0	0
Pempheriformes_Marine brackish	1	0	0
Perciformes_Brackish	1	0	0
Perciformes_Euryhaline	16	7	43.75
Perciformes_Freshwater	264	4	1.515151515
Perciformes_Freshwater brackish	22	6	27.27272727
Perciformes_Marine	1972	18	0.912778905
Perciformes_Marine brackish	111	2	1.801801802
Percopsiformes_Freshwater	8	1	12.5
Pholidichthyiformes_Marine	1	0	0
Pleuronectiformes_Brackish	4	0	0
Pleuronectiformes_Euryhaline	32	4	12.5
Pleuronectiformes_Freshwater	26	0	0
Pleuronectiformes_Freshwater brackish	8	0	0
Pleuronectiformes_Marine	500	4	0.8
Pleuronectiformes_Marine brackish	63	0	0
Polymixiiformes_Marine	10	0	0
Polypteriformes_Freshwater	11	0	0
Salmoniformes_Euryhaline	41	26	63.41463415
Salmoniformes_Freshwater	125	5	4
Salmoniformes_Freshwater brackish	7	1	14.28571429
Scombriformes_Euryhaline	2	0	0
Scombriformes_Marine	226	23	10.17699115
Scombriformes_Marine brackish	16	2	12.5
Siluriformes_Brackish	4	0	0
Siluriformes_Euryhaline	21	1	4.761904762
Siluriformes_Freshwater	2947	38	1.289446895
Siluriformes_Freshwater brackish	52	4	7.692307692
Siluriformes_Marine	10	0	0
Siluriformes_Marine brackish	58	0	0
Spariformes_Euryhaline	5	1	20
Spariformes_Freshwater brackish	1	0	0
Spariformes_Marine	184	1	0.543478261
Spariformes_Marine brackish	37	3	8.108108108
Stomiatiformes_Marine	337	0	0
Stylephoriformes_Marine	1	0	0
Synbranchiformes_Freshwater	98	0	0
Synbranchiformes_Freshwater brackish	6	0	0
Syngnathiformes_Brackish	1	0	0
Syngnathiformes_Euryhaline	14	0	0
Syngnathiformes_Freshwater	14	0	0
Syngnathiformes_Freshwater brackish	9	0	0
Syngnathiformes_Marine	387	0	0
Syngnathiformes_Marine brackish	41	1	2.43902439
Tetraodontiformes_Brackish	2	0	0

Tetraodontiformes_Euryhaline	8	1	12.5
Tetraodontiformes_Freshwater	13	0	0
Tetraodontiformes_Freshwater brackish	5	0	0
Tetraodontiformes_Marine	284	0	0
Tetraodontiformes_Marine brackish	37	0	0
Uranoscopiformes_Euryhaline	2	1	50
Uranoscopiformes_Freshwater	1	0	0
Uranoscopiformes_Marine	136	0	0
Uranoscopiformes_Marine brackish	6	0	0
Zeiformes_Marine	29	0	0
