# Supplementary material for: Evidence for general size‐by‐habitat rules in actinopterygian fishes across nine scales of observation
Source: Ecol Lett. 2021 Jun 10;24(8):1569–81. doi: 10.1111/ele.13768 (PMC8362132; doi:10.1111/ele.13768)
Supplement: Supplementary file 5 — Supplementary Material [file ELE-24-1569-s016.pdf]

Title: **Evidence for general size-by-habitat rules in actinopterygian fishes across nine scales of observation**

Expanded Materials and Methods

Data sources

Habitat-use definitions were separately obtained and analysed from both FishBase (Froese & Pauly 2019) and Catalogue of Fishes (Fricke *et al.* 2020) (referred to as FB and CoF throughout). The CoF dataset was used in main text figures as the data appears more closely vetted and updated by specialists, and therefore may represent a more reliable source of habitat codings. However, FishBase data have other advantages (e.g. better taxon overlap with the phylogenies employed in this study) and therefore both sources should be able to reveal general size-by-habitat-use patterns. Thus, I examined both to establish whether database choice meaningfully impacts results. Both coding sources label taxa as either absent or present in brackish, marine and freshwater environments. From this, I derive six habitat-use types that reflect each unique combination of states. This includes taxa known exclusively from either i) marine; ii) freshwater; and iii) brackish settings; those reported as freshwater in combination with brackish, iv) freshwater-brackish; those reported as marine in combination with brackish, v) marine-brackish; and taxa coded as both marine and freshwater, broadly defined here as vi) euryhaline. As extremely small numbers of taxa were exclusively brackish, these were not subject to detailed pairwise comparisons (*but see* Fig. S1).

Comparing an 11k molecular phylogeny with 31k supertrees

A key motivation for performing analyses on both the 11k and 31k trees was to test whether size distributions systematically differ between more representative taxon sampling (31k trees) and taxa selected for molecular sampling (11k tree). For example, if scientists, in an extreme hypothetical scenario, strictly sampled a specific size class when selecting taxa for molecular data (e.g. taxa between 75cm and 100cm), the size distributions of every habitat may look similar in the 11k-tree datasets, regardless of the true underlying differences between habitats. For the data analysed here, I show the size distributions of all habitats for both CoF and FB versions of the 11k- and 31k-tree datasets in Figure S1. This reveals that there is a size bias introduced by molecular sampling; the entire size distribution of every habitat (including the mean and median of each) are shifted towards larger sizes in the 11k-tree datasets relative to their 31k-tree dataset counterparts (be careful to note the subtly different scale bars between plots). However, importantly, the relative order of these size distributions, from smallest to largest, and the variance of sizes within these habitats, remain highly comparable between the 11k- and 31k-tree datasets.

Nevertheless, there are subtle adjustments in the relative position of habitats worthy of note, as these have the potential to alter some downstream comparisons. First, the median size of freshwater and freshwater-brackish taxa are further apart from the marine median size in the 11k-tree datasets relative to the 31k-tree datasets (Fig. S1). This may explain why the percentage of clades in which marine taxa possessed larger means (and larger phylogenetic means) than freshwater and freshwater-brackish taxa (within each taxonomic scale)

are often higher in 11k-tree datasets than in 31k-tree datasets (Fig. S3–4). Second, the marine median is closer to the euryhaline and marine-brackish medians in the 11k-tree datasets relative to the 31k-tree datasets (Fig. S1). This appears to reduce the percentage of clades (within each taxonomic scale) in which the marine-brackish and euryhaline taxa were larger than their marine relatives in the 11k-tree datasets relative to the 31k-tree datasets (Fig. S3–4). Thus, any reader interested in the result of a specific clade from this study, particularly if it falls into the two sets of comparisons outlined above, may benefit for checking whether that specific result was potentially influenced by the molecular size bias by examining Appendices 2–17. However, for the main goal of this study, although choice of a molecular tree can slightly alter size differences observed between these specific habitats, the main size-by-habitat patterns (regarding which habitat contained the larger taxa in a majority of clade comparisons relative to another habitat) nevertheless emerge consistently across datasets. It does however raise the prospect that this molecular size bias (and other traits that may experience bias through molecular sampling) is likely to have had at least some influence on the results of previous studies of body size, not only of fishes but more generally across the Tree of Life. It would be therefore beneficial to attempt to document this phenomenon, and where possible correct for it, in future studies of any traits potentially influenced by molecular sampling bias.

Another reason to run all analyses on both 11k- and 31k-tree datasets is to assess whether other differences between these phylogenies may influence the results. Both phylogenies have their potential advantages, with 11k trees being of higher resolution, but containing a far more limited fraction of total diversity relative to 31k trees. This higher resolution of the 11k phylogeny may also be associated with molecular taxon selection biases (as demonstrated for size distributions) that may alter our interpretation of evolutionary dynamics. 31k trees are more representative in the taxa they contain, but require rules to resolve polytomies and branch lengths of those inserted taxa, and choices regarding the taxonomy used will influence how conservatively missing taxa are placed (Siqueira *et al.* 2020). Given the goal of this study was to test whether broad trends regarding which habitat possessed the larger body size in a substantial majority of clades for each habitat comparison at each taxonomic scale, it is reassuring that these were consistent between the 11k and 31k datasets (Figs. S3–S4). However, for a researcher interested in a specific clade outcome, caution should be taken when interpreting results, particularly for smaller groupings such as family that may be more heavily influenced by the misplacement of taxa in proportion to their overall diversity. Therefore, in those instances, I recommend examining the result across the four datasets here using the appendices (for main size analyses, see appendices 2–5, for trophic analyses see appendices 10–13) and examining the precise membership of their clade of interest in each dataset.

Comparing 'maximum length' and common length'

I thought it would also be useful to consider how the 'maximum length' data used in the main text may compare to another possible measure of size ('common length' as provided in FishBase, available for a maximum of 3484 species with the methods employed here), because I reason that characterising

macroevolutionary patterns with a variety of size metrics will ultimately be necessary to more rigorously identify and understand the mechanisms underpinning various broad scale size patterns.

It was my intention to compare 'maximum length' and 'common length' for shared taxon sets, but also using different habitat coding sources and phylogenies as in the main text. Thus, for each of the four datasets used in the main text (FB11k, CoF11k, FB31k and CoF31k), a 'maximum length' and 'common length' version of each can be made. In total then, I derived eight new datasets, first by obtaining those species for which both 'maximum length' and 'common length' data are available – a 'shared size' taxon list. Second, I pruned each of the original four datasets down to the taxa they individually shared with the 'shared size' taxon list. This yielded an 11k-tree FB dataset for 2610 species, an 11k-tree CoF dataset for 2488 species, a 31k-tree FB dataset of 3484 species, and a 31k-tree CoF dataset for 3307 species. To one set of these four pruned datasets, 'common length' data were added, and to another set of these four pruned datasets, 'maximum length' data were added, yielding eight datasets in total (e.g. 'maximum length' FB11k dataset and 'common length' FB11k dataset, etc.)

As expected, the 'maximum length' datasets have larger sizes in every habitat-use category, yet the relative position of each habitat's size distribution, and the spread of sizes within habitats, are largely comparable to those for 'common length' (Fig. S10; yet note marginally higher variance in 'maximum length' data versions of freshwater-brackish and euryhaline, while also noting these habitats still display greater variance than other habitats in the 'common length' data). Thus, 'maximum length' data resemble 'common length' data when taxon sampling is identical. However, both of these datasets differ in a more substantial feature when compared with the complete 'maximum length' 11k- and 31k-tree datasets used in the main text. Specifically, while marine, euryhaline, and marine-brackish taxa possess highly comparable means in the 'maximum length' and 'common length' datasets, mean sizes are considerably more staggered across the freshwater-brackish through marine-brackish continuum in the complete 'maximum length' datasets (compare 'maximum length' datasets in Fig. S10 to complete 'maximum length' datasets in Fig. S1). This observation suggests this mismatch may result from a bias in the 'common length' data, where taxa from these specific habitats are selected from a more similar size range. It suggests this because, if the staggered habitat means were a persistent and unique feature of the 'maximum length' data, we should expect that same pattern to appear in the smaller version of that dataset in Figure S10. One possible alternative explanation for the 'staggered mean' pattern disappearing is a scenario where the particular subset of taxa for which 'common length' data exist happens to represent an unusual subset where the relationship between common length and maximum length are atypically well aligned. If true, this could mean that comparisons of these two datasets may not agree with different taxon sampling in future research.

Another observation that suggests a bias in the 'common size' data are the smaller differences seen between means for the same habitat-use between the 11k- tree and 31k-tree versions of the 'common length' datasets

(Fig. S10). This is because, as illustrated by the complete 'maximum length' dataset, we expect a clear inflation of means in 11k-tree datasets relative to 31k-tree datasets due to the molecular sampling size bias described above (Fig. S1). Thus, the absence of this expected size inflation on molecular trees likely suggests that the entire 'common length' taxon sample represents an atypical sample, that cannot be made more representative by also examining those not selected for molecular sampling.

In the absence of unambiguous data to the contrary, it is fair to read that 'maximum length' and 'common length' data reflect each other reasonably well, suggesting results from macroevolutionary studies using 'maximum length' may also be broadly reflective of 'common length' patterns, but these issues warrant further examination with new data. Either way, similarity between these metrics is not a prerequisite for their utility; both metrics theoretically represent useful phenotypic measures with which to reveal information on macroevolutionary patterns and processes.

Scales of observation

A key goal of the study was to examine whether the outcomes of the comparisons cascade through multiple scales of observation, or are restricted to specific scales. This is because the handpicking of clades, either by random choice or by design, commonly referred to as ascertainment bias, represents a fundamental and important challenge in evolutionary biology (Beaulieu & O'Meara 2018). It also allows me to address whether inferences from 11k and 31k phylogenies vary differently with scale, and whether evolutionary hotspot sections of these phylogenies, that an investigator might sensibly choose, deliver representative results.

The analytical groupings are therefore devised with several goals in mind. First, they are designed to provide results for taxonomic units that are relatable to researchers, namely family and order groupings. This makes the results more accessible, and enables comparison with past and future studies which tend to examine these groupings. However, because specific anatomical and biological traits underpin the definition of such groupings, their use as analytical units can bring additional benefits, as these biological factors may commonly help to explain observed patterns. For example, when Romanuk et al. 2011 documented a relationship between body size and trophic level in actinopterygians fishes and chondrichthyans, when exceptions to this general pattern emerged, specific biological innovations of the clades analysed often provided a plausible explanation (e.g. schooling behaviour in Atheriniformes [silversides]). This exact clade example may similarly explain some atypical size results for Atheriniformes in my study. For example, note how Atheriniformes commonly appear among the 'minority results' in Figure S15 (e.g. marine-brackish members of this clade are not larger than their marine taxa, as is seen in most other clades; Fig. 1b).

Second, above the order level, successive groupings are not tightly mapped onto named taxonomic ranks (doing so could have meant that several ranks could all have referred to the same set of species in some instances) but combine groupings to capture increasingly larger segments of actinopterygian diversity. This

approach can both provide: i) the changing perspective of successively broader scales of observation, examining ever larger sections of phylogeny where each segment contains more comparable species richness; and ii) details on specific sections of the phylogeny (e.g. highly nested sections of the phylogeny, or examination of early diverging lineages).

Third, I also examine patterns in evolutionary hotspots for the trait in question (i.e. here, clades that explore a wide variety of habitats). Such groups form a popular choice for individual studies in evolutionary biology, but whether we can successfully glean general patterns from clades that are in of themselves somewhat atypical (i.e. in their ability to widely explore the trait of interest) remains an open question (see **"Importance and utility of taxonomic scale / scale of observation"** SI discussion section below for details).

The scales of observation examined are defined as follows: i) family and ii) order (both as defined in Rabosky *et al.* 2018); iii) Tax3, a custom set of groupings comprising collections of orders, dividing the tree into 13 segments; iv) Tax4, can be thought of as 'Series plus', where Series definitions of Rabosky *et al.* 2018 were combined with comparably sized multi-order groupings for the rest of the tree, yielding 9 groups in total; v–vi) Tax5 and Tax6 represent two additional higher-level groupings that involved dividing the tree into 5 and 3 segments, respectively; vii) the full dataset; viii) evolutionary hotspots, perhaps analogous to studying Darwin's finches or Hawaiian honeycreepers at lower taxonomic levels, but here represent hand-picked sections of the phylogeny where the potential driver of interest (habitat use) varies greatly; ix) a scale of customised 'expanded hotspots', which contains two types of expanded hotspots. In the first type, all hotspot clades are expanded to also include closely related clades that show less habitat variation, yet still contained some diversity of marine and freshwater taxa. In the second type, the type one expanded hotspots were then broadened even further to include other adjacent sections of phylogeny that may display more homogenous habitat use. By this approach, some clades contribute to more than one comparison, yet it offers a way to examine broader dynamics in parts of the tree surrounding hotspot regions.

Assessing differences in size between habitats
Five metrics were used to assess differences in log10 body size between habitat-use type: i) log10 means; ii) phylogenetic means (LS mean in RRPP); iii) Wilcoxon tests; iv) phylogenetic ANOVA (phytools, Revell 2012); and v) PGLS ANOVA (RRPP, Adams & Collyer 2018; Collyer & Adams 2019).

First, I compared log10 habitat means to show the differences present between habitats before phylogeny is considered, capturing a snapshot of the raw size patterns that we observe in nature. Second, I compared phylogenetic means (LS mean output from R package RRPP) in an attempt to obtain more representative size averages for these habitats once evolutionary history is considered. Third, I compared size between habitats using Wilcoxon tests, principally employed to highlight which comparisons possess sufficient sample sizes and notable differences in log10 mean size in the original data. Use of Wilcoxon tests also permits direct

comparison of the results with a recent study comparing size between marine and freshwater fishes (Sanchez-Hernandez & Amundsen 2018). Fourth and fifth, I compared size with simulation based phylogenetic ANOVA in R package phytools (Revell 2012) and from PGLS ANOVA in R package RRPP (Adams & Collyer 2018; Collyer & Adams 2019), respectively.

The five metrics of comparison chosen here are well suited to address the goals of the study, and the phylogenetic approaches were selected for their desirable properties, including infrastructure for pairwise testing, the ability to extract phylogenetic means, and the calculation of effect sizes. They also enabled me to compare results from one of the most widely implemented methods (e.g. simulation ANOVA) to a novel implementation (RRPP). I consider RRPP to be more methodologically appropriate implementation because its parameter estimates are conditioned upon the phylogeny. This is not true of the simulation approach, which should primarily be considered as a tool to derive *p* values. Larouche *et al*. 2020 outlined a theoretical circumstance where simulation ANOVA may prove useful in this regard, a point that could benefit from further exploration. Both methods are efficient to implement over many clades and iterations of large trees; an essential requirement given my fundamental goal to report (and test for consistent alignments of) size differences from thousands of comparisons across nine scales of observation using four large datasets (in which, for 31k tree matched datasets, every comparison is run over 100 supertrees). Together, the analyses as implemented took ~2 months to run and some analyses appeared to require 18+ GB of RAM.

Each metric was computed for every pairwise habitat-use comparison in the study (consisting of every clade at all nine scales of observation where any of the ten pairwise habitat pairs could be compared). This was repeated across the four datasets that represent tree type and habitat coding source (FB11k; CoF11k; FB31k; CoF31k). Due to computational demands, the full-dataset PGLS ANOVA was run on one 31k-tip phylogeny. For all other comparisons at lower taxonomic scales, metrics requiring trees were obtained from all 100 trees and means of these values were then obtained for those display items (e.g. phylogenetic means; Fig. 2a). Where *p* values are the metric under examination, because the direction of interpretation for a *p* value may vary over a sample of trees (e.g. where the phylogenetic mean for habitat A is larger than habitat B in 90 trees, but habitat B is larger in 10 trees), the mean *p* value was obtained from the most common outcome (i.e. the mean *p* from the 90 trees). These averaged *p* values are then used in display items (e.g. Appendices 2–5).

Assessing differences in size variance between habitats

First, variance was calculated based upon the observed log10 size data for each habitat use to illustrate size variety present in nature (Fig. S5a). Second, because size variation in a habitat will depend greatly on the structure of the phylogeny (e.g. a habitat use that arises in distant parts of phylogeny within a clade would be expected to possess more size variety under a null model than a similarly species rich group limited to a single subclade), I estimated and compared variance between every habitat comparison based upon simulated size data. To achieve this, Brownian motion simulations of body size evolution were run upon the topology 1000

times, and for the comparison of interest, the mean variance of each habitat across those simulations was obtained and compared (Fig. S5b). Third, because neither of the above tells us which habitat (for a given comparison) possesses more variance than expected by simulation, I needed to ask where the observed variance ratio for a comparison (e.g. variance of marine Beloniformes divided by variance of freshwater Beloniformes) fell relative to the 1000 ratios obtained from simulated data (variances of marine Beloniformes for each simulation divided by variance of freshwater Beloniformes for each simulation). From this, I could both deduce which habitat possessed more variance than expected (Fig. S5c) and provide an associated *p* value (Fig. S11).

Expanded Results

Size-by-habitat-use patterns

Considering comparisons of observed log10 means for the CoF 31k-tree dataset (rather than comparisons of phylogenetic mean shown in the main text, Fig. 2a), euryhaline and marine-brackish taxa are larger on average than taxa in other habitats (freshwater, marine, freshwater-brackish); an outcome seen a high percentage of comparisons within every taxonomic scale in all datasets (~70–100%; top six rows of Fig. S4, Appendices 2–5; note the marine-brackish vs. freshwater-brackish family level exception). An equally strong finding is that freshwater-brackish means are larger than freshwater means, a pattern that occurs in a high percentage of clade comparisons within every taxonomic scale in all datasets; (~75–100%; Fig. S3, Appendices 2–5). Another strong finding is that marine means are larger than freshwater means, occurring in moderate to large percentages of clade comparisons within every taxonomic scale in all datasets (65%–100%; Fig. S4, Appendices 2–5). Mean size differences between marine taxa and freshwater-brackish taxa, as well as between marine-brackish and euryhaline taxa, show no consistent pattern, with different results obtained across different taxonomic scales (Fig. S4, Appendices 2–5).

Size-variance-by-habitat-use patterns

Relative to comparisons of size differences, there is weak evidence for clear and consistent size-variance-by-habitat patterns across taxonomic scales. When comparing observed log10 size variance (Fig. S5a, Appendices 14–17), the most consistent results are: i) freshwater-brackish taxa possess greater variance than marine, freshwater, marine-brackish and euryhaline taxa in a narrow majority of comparisons within most, but not all, taxonomic scales (Fig. S5); ii) euryhaline taxa possess greater variance than marine, freshwater and marine-brackish taxa in a large majority of comparisons at high taxonomic scales only (Tax5, Tax6, full dataset).

It is possible that the two variance patterns above are explained by tree structure, as euryhaline and freshwater-brackish taxa regularly arise from distantly related taxa. This is further supported by the observation that their taxa can derive from several habitats (Fig. 1d). To examine this, I plot simulated variance comparisons (Fig. S5b), and show they can replicate many aspects of patterns i) and ii) described above.

Beyond observed and simulated variance, the most reliable test is to ask, for every comparison, is which habitat possessed more variance in the observed data relative to simulations (Fig. S5c, see expanded method section above). These analyses clearly confirm findings i) and ii), demonstrating that they are not solely driven by expectations from simulations (Figs. 5c, S11, Appendices 14–17). Observed differences in variance are rarely large enough relative to simulations to deliver low *p* value results, particularly at high taxonomic levels (Fig. S11, Appendices 14–17).

Inferences drawn from several statistical approaches at multiple scales

I employed three statistical approaches to reveal the probability (*p*) of size difference for every comparison. The spread of low *p* results can then be evaluated across all habitat comparisons and taxonomic scales ($p < 0.1$ and $p < 0.05$ differentiated by colour shade, e.g. PGLS ANOVA results, Fig. S12) alongside information regarding absolute magnitudes and ratios of size differences and effect sizes (Appendices 2–5). To visualise differences between statistical approaches, I present all tests at the order scale in Figure S13a (exact *p* values in Fig. S14), and visualise all PGLS ANOVA results for one dataset to reveal how low *p* results are distributed across taxonomic scales (Fig. S13b, exact *p* values in Fig. S12; see Appendices 2–5 for all test information across all datasets). I provide comment on three general points regarding outcomes across the different methods below.

First, low *p* value results mostly occur in comparisons sharing the directionally of previously highlighted majority outcomes (e.g. when marine-brackish taxa possess larger means than marine taxa in 75% of order comparisons, a vast majority of low *p* results occur in orders where marine-brackish taxa were larger; Fig. S13a, b; Appendices 2–5).

Second, when the number of low *p* value results are compared between traditional and phylogenetic tests, a number of important deviations come to light. When comparing all orders for a given pairwise habitat comparison (e.g. marine-brackish vs. marine, fifth row of Fig. S13a), it is typical that the number of low *p* value results ($p<0.1$) from Wilcoxon test are approximately double the number of low *p* value results seen from either of the two phylogenetic tests (simulation ANOVA or PGLS). However, there are notable deviations from this general ‘double Wilcoxon’ pattern. For example, for marine vs. freshwater order comparisons, even the maximum number of low *p* value results from either phylogenetic test is very small, around four times smaller than recovered with Wilcoxon tests (Fig. S13a). At the other extreme, for freshwater vs. freshwater-brackish comparisons, the number of low *p* value results from a phylogenetic test can equal the number obtained by Wilcoxon tests (simulation ANOVA, freshwater vs. freshwater-brackish, Fig. S13a). These deviations likely highlight instances where phylogenetic structure itself increases (e.g. freshwater vs. freshwater-brackish, where transitions between habitats are common) or decreases (marine vs. freshwater, where transitions are rare and often occur via an intermediate habitat use; Fig. 1d) our ability to detect low *p* value results. This has important implications, as it may lead us to overlook important phenotypic differences in the latter instance,

even if by other measures, differences are no less substantial. It is also conceivable that specific evolutionary processes may repeatedly undermine our ability to detect differences they generate, such as if a lineage transitioning to freshwater quickly decreases in size, and then that small taxon rapidly diversifies (a scenario where size change may be substantial but statistically unexceptional, because a scenario where many young taxa share the size of their evolutionarily young small ancestor would be expected under a null model). This possibility underlines why I examined mean comparisons for all clades at many scales (e.g. Fig. 2a), as consistent differences in means may highlight important broader trends that sole focus on individual low *p* results may obscure.

Third, examination of *p* values and effect sizes (statistics corresponding to Fig. S13b are shown in Fig. S12; Appendices 2–5) show that comparisons with low *p* values are commonly associated with larger effect sizes. Effect sizes remain comparably large (or are larger) at high taxonomic scales relative to low taxonomic scales, illustrating that increases in sample size alone are not the primary driver of low *p* results at high taxonomic scales.

<u>Expanded Discussion</u>

Possible explanations of size-variance-by-habitat patterns

Few size-variance-by-habitat patterns are as consistent as absolute size patterns, but a small majority of clades show greater size variance in freshwater-brackish habitats relative to other habitat-use types at several taxonomic scales (Fig. 5c). Plausible explanations of greater freshwater-brackish size variance include that this habitat i) receives species from habitats at opposite ends of the size extreme (Fig. 1d; small freshwater, large euryhaline/marine-brackish); ii) may contain a high proportion of newly arrived species (including invasive species) from these size extremes, resulting in greater size variation, as selection would have less time to guide taxa towards local size optima; and iii) has a relatively high proportion of migratory taxa (Table S3) of large size, and so their contrast with small taxa of freshwater origin increases variance.

Importance and utility of taxonomic scale / scale of observation

Finally, this study adds to literature that highlights the utility of considering multiple comparisons, the scale of observation, and taxon sampling in efforts to overcome ascertainment bias and gain a more complete understanding of evolutionary process (Hopkins & Smith 2015; Beaulieu & O'Meara 2018). For example, the data revealed the ability of taxon sampling to inflate size estimates in all habitats (see SI methods above), and how ‘hotspot’ clades – those clades selected because the trait of interest (habitat use) varies considerably (including ‘expanded hotspots’) – are no more likely to give the representative outcome (e.g. euryhaline larger than freshwater-brackish) than a relatively low-level clade (e.g. order) selected at random (Fig. 2a). This highlights the importance of performing many comparisons, yet shows how the study of many

hotspots/extended hotspots may sometimes offer a less labour-intensive way to obtain a generalisable pattern.

It is worth noting that while evolutionary hotspot clades did reveal the typical size-by-habitat pattern when they were examined together, individually these clades often showed quite atypical dynamics in several ways. For instance, hotspots corresponding to entire orders include: Atheriniformes, Beloniformes, Clupeiformes, Gobiiformes and Tetraodontiformes. One of the strongest patterns from this study was the clear possession of larger size in euryhaline environments vs. i) freshwater, ii) marine, and iii) freshwater-brackish settings. However, most of the rare exceptions (i.e. outcomes seen in a minority of comparisons at each taxonomic scale) to these patterns derive from these hotspot clades, with Tetraodontiformes atypical with respect to finding i) above (i.e. freshwater Tetraodontiformes are larger than marine Tetraodontiformes); Beloniformes atypical relative to finding ii) above, and Atheriniformes atypical relative to findings i), ii) and iii) above (Fig. S15). In fact, Atheriniformes, Beloniformes, Clupeiformes and Gobiiformes commonly feature among the minority outcomes (i.e. opposite to the standard size-by-habitat pattern observed in a majority of clades for each pairwise habitat comparison) in most pairwise habitat comparisons (Fig. S15).

Even where size patterns were typical, these hotspot clades were commonly found among outliers in a separate context: they appeared more likely to show mismatches between discrete size and trophic outcomes. Most prominent in this regard were the Clupeiformes, whose size pattern outcomes were typical, but whose trophic patterns did not align in eight out of the ten pairwise habitat comparisons (Clupeiformes plot within the grey quadrant in 8/10 habitat comparisons; Fig. S6). Furthermore, notable size–trophic mismatches are seen in i) Gobiiformes in euryhaline vs. freshwater-brackish comparisons, and freshwater-brackish vs. marine-brackish comparisons; ii) Beloniformes in euryhaline vs. marine-brackish comparisons; and iii) Atheriniformes in freshwater vs freshwater-brackish, and freshwater-brackish vs. marine comparisons (Fig. S6).

The above observations may suggest that clades which are unusually good at exploring the trait of interest (in this case, salinity environment), may achieve this precisely because they are extraordinary in some respect, and therefore may not be as likely to show evolutionary dynamics that are typical for the trait of interest. In some instances, it could also be that the particularly complex evolutionary history of these clades (precisely because they frequently move between environments) makes it far more difficult to calculate representative phylogenetic means for some habitats, increasing the likelihood of delivering an atypical result in some instances. Potential evidence for this interpretation is that these hotspot clades delivered an atypical result less frequently in comparisons of observed (i.e. non-phylogenetic) log10 size means (Fig. S1). However, this is not conclusive evidence, given it is perfectly possible that phylogenetic means are still valid, even when the observed log10 pattern disagrees. Indeed, disagreement between observed and phylogenetic patterns could be expected, if a habitat use frequently permits only a few taxa to occupy high tropic levels and possess large size. However, the prevalence with which observed and phylogenetic mean comparison outcomes differ in these

hotspots clades is at least permissive to the idea that complex character histories present challenges for phylogenetic mean estimation. This should be further explored in future research by developing new and more clearly resolved phylogenies for these clades, and combining these with high-resolution habitat data to provide detailed documentations of their habitat histories prior to reanalysis, potentially with a variety of models of morphological evolution.

An alternative approach to discover representative evolutionary phenomena might be to study large clades (e.g. Tax5, Tax6 and above), given in this study, they often captured the general pattern, giving unanimous results where large majorities for an outcome existed at low scales (e.g. marine-brackish vs. freshwater; Fig. 2a), or comparably sized (or larger) majorities where majority sizes at lower scales (e.g. order) were moderate (e.g. freshwater-brackish vs. freshwater; Fig. 2a). This raises the possibility that low, but representative sampling at large scales may reveal general patterns. However, it should be noted that the striking agreement in size patterns across scales observed here may prove to be atypical. This is because compelling arguments have been made for why this may not be expected for size in many terrestrial animals (Slater & Friscia 2019; Potapov *et al.* 2019) or in any group of organisms where differences in size are more evolutionarily important at local scales between species than they are between higher-level clades. This reinforces the need to examine patterns at multiple scales. Size in fishes may therefore represent a key trait in exploring specific macroevolutionary phenomena that are more difficult to assess for terrestrial lineages.

SI References

Adams, D.C. & Collyer, M.L. (2018). Phylogenetic ANOVA: Group-clade aggregation, biological challenges, and a refined permutation procedure. *Evolution*, 72(6), 1204–1215.

Beaulieu, J.M. & O'Meara, B.C. (2018). Can we build it? Yes we can, but should we use it? Assessing the quality and value of a very large phylogeny of campanulid angiosperms. *Am. J. Bot.*, 105(3), 417–432.

Collyer, M.L. & Adams, D.C. (2019). RRPP: Linear Model Evaluation with Randomized Residuals in a Permutation Procedure. https://CRAN.R-project.org/package=RRPP

Fricke, R., Eschmeyer, W.N. & van der Laan, R. (2020). Eschmeyer's catalog of fishes: genera, species, references. Available at: http://researcharchive.calacademy.org/research/ichthyology/catalog/fishcatmain.asp.

Froese, R. & D. Pauly. Editors. 2019. *FishBase*. World Wide Web electronic publication, www.fishbase.org, (12/2019).

Hopkins, M.J. & Smith, A.B. (2015). Dynamic evolutionary change in post-Paleozoic echinoids and the importance of scale when interpreting changes in rates of evolution. *Proc. Natl Acad. Sci.*, 112(12), 3758–3763.

Larouche, O., Benton, B., Corn, K.A., Friedman, S.T., Gross, D., Iwan, M., Kessler, B., Martinez, C.M., Rodriguez, S., Whelpley, H., Wainwright, P.C. & Price, S.A. (2020). Reef-associated fishes have more maneuverable body shapes at a macroevolutionary scale. *Coral Reefs*, 39(5), 1427–1439.

Potapov, A.M., Brose, U., Scheu, S. & Tiunov, A.V. (2019). Trophic Position of Consumers and Size Structure of Food Webs across Aquatic and Terrestrial Ecosystems. *Am. Nat.*, 194(6), 823–839.

Rabosky, D.L., Chang, J., Title, P.O., Cowman, P.F., Sallan, L., Friedman, M., Kaschner, K., Garilao, C., Near, T.J., Coll, M. & Alfaro, M.E. (2018). An inverse latitudinal gradient in speciation rate for marine fishes. *Nature*, 559(7714), 392–395.

Revell, L.J. (2012). phytools: an R package for phylogenetic comparative biology (and other things). *Methods Ecol. Evol.*, 3(2), 217–223.

Romanuk, T.N., Hayward, A. & Hutchings, J.A. (2011). Trophic level scales positively with body size in fishes. *Glob. Ecol. Biogeogr.*, 20(2), 231–240.

Sanchez-Hernandez, J. & Amundsen, P.A. (2018). Ecosystem type shapes trophic position and omnivory in fishes. *Fish Fish.*, 19(6), 1003–1015.
