# Supplementary material for: Evidence for general size‐by‐habitat rules in actinopterygian fishes across nine scales of observation
Source: Ecol Lett. 2021 Jun 10;24(8):1569–81. doi: 10.1111/ele.13768 (PMC8362132; doi:10.1111/ele.13768)

Size var results from fb 11k phylogeny dataset: all.scales.at.once

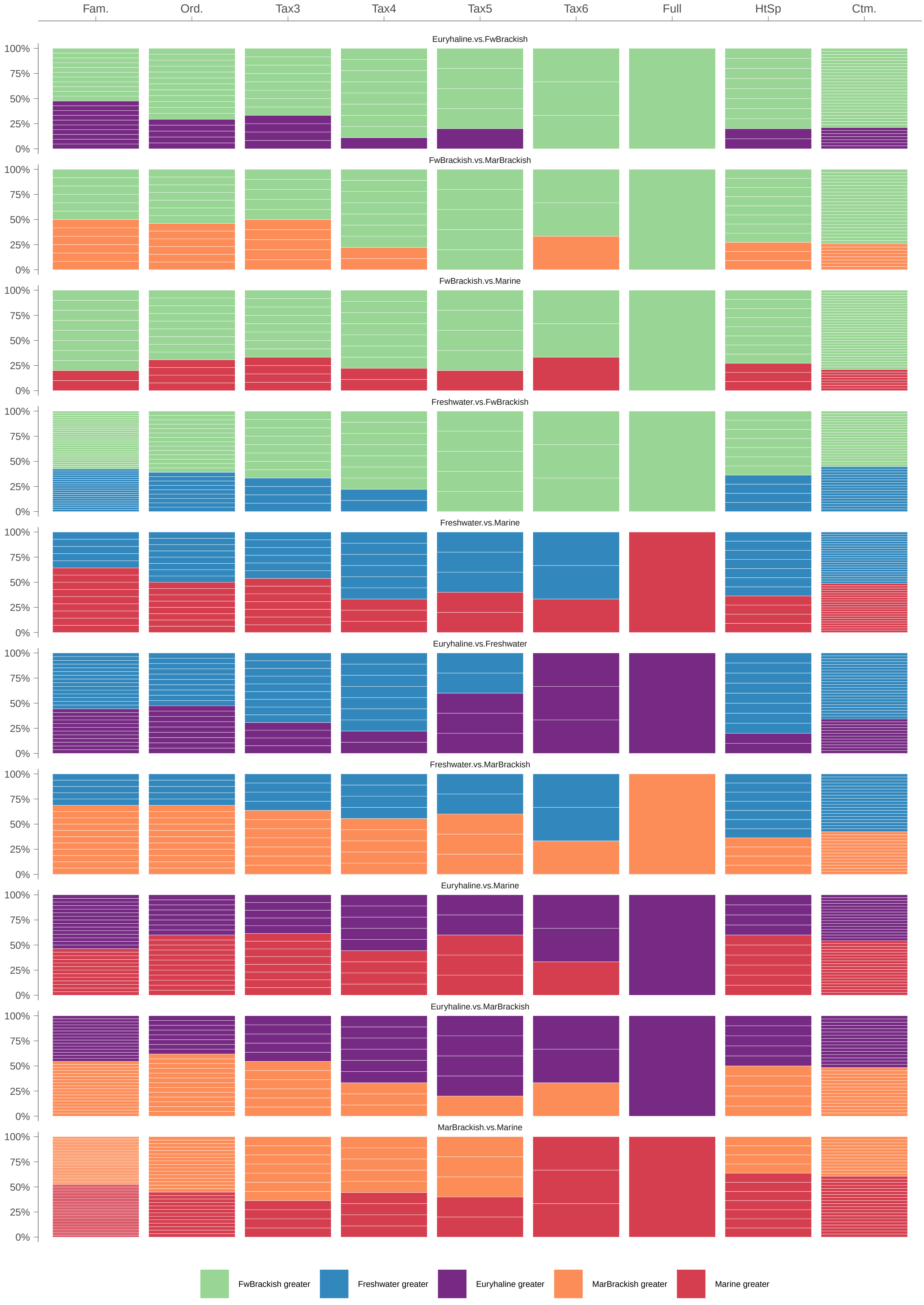

Size var results from fb 11k phylogeny dataset with statistics: all.scales.at.once

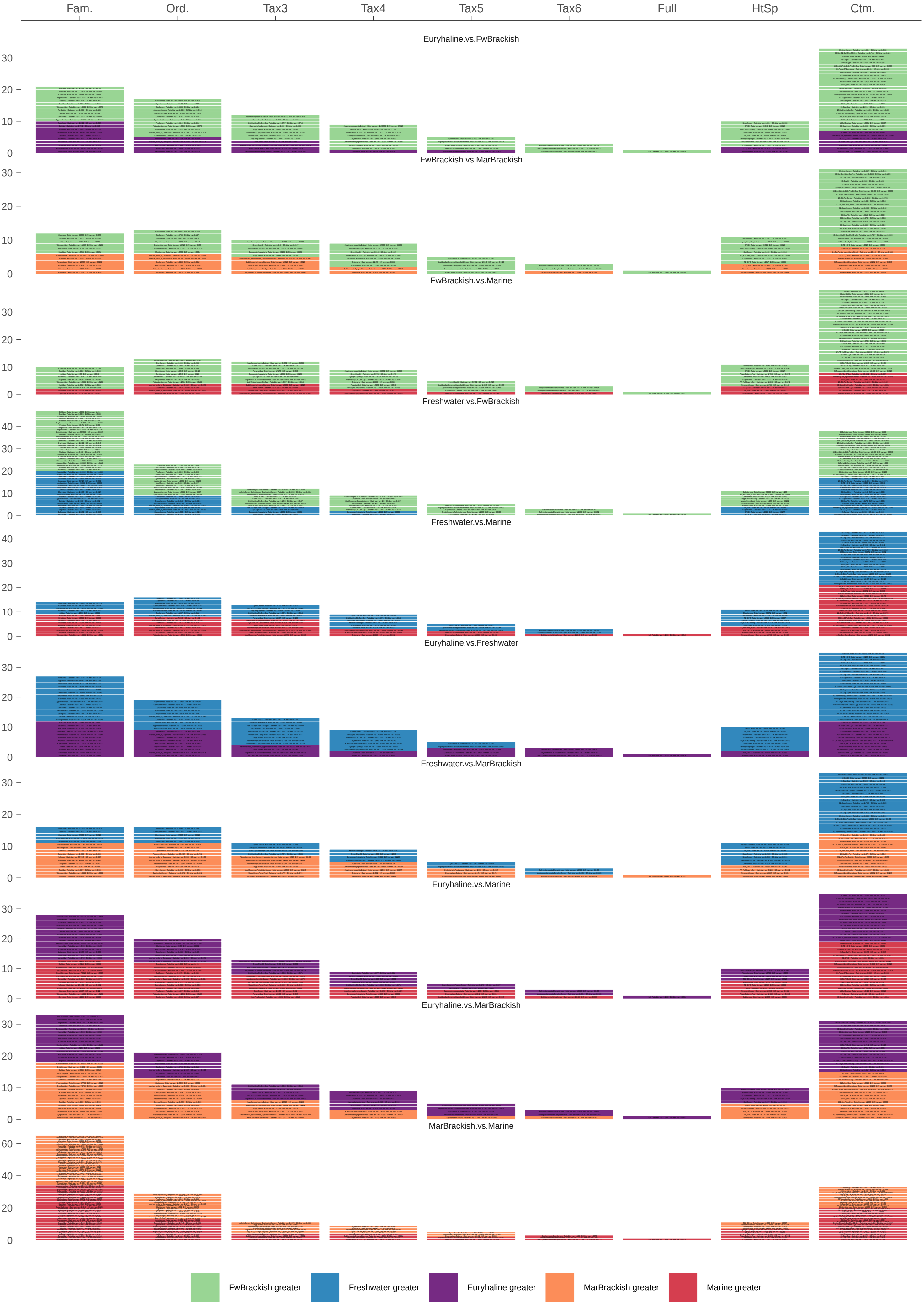

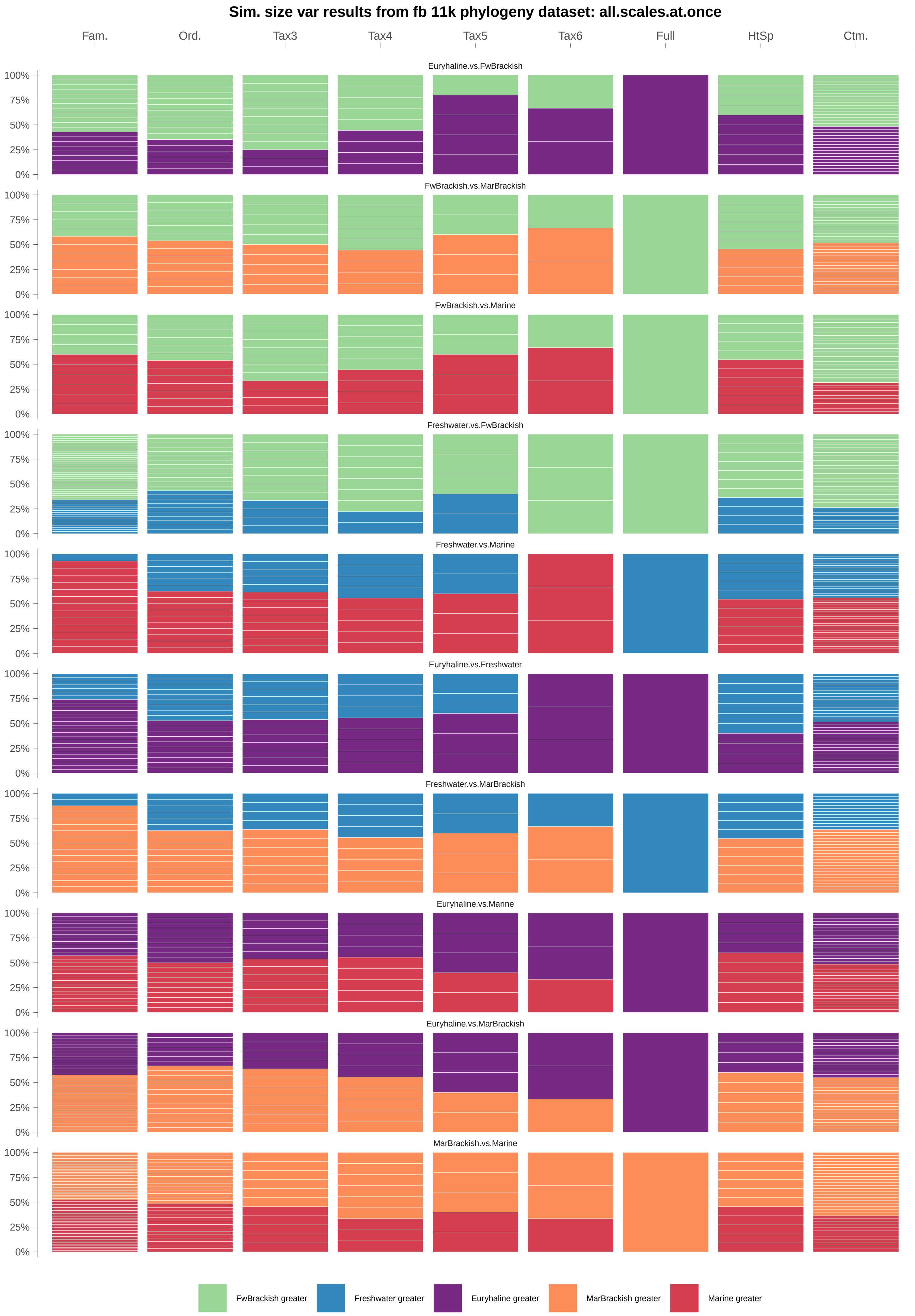
Sim. size var results from fb 11k phylogeny dataset: all.scales.at.once
Fam.
Ord.
Tax3
Tax4
Tax5
Tax6
Full
HtSp
Ctm.
Euryhaline.vs.FwBrackish
FwBrackish.vs.MarBrackish
FwBrackish.vs.Marine
Freshwater.vs.FwBrackish
Freshwater.vs.Marine
Euryhaline.vs.Freshwater
Freshwater.vs.MarBrackish
Euryhaline.vs.Marine
Euryhaline.vs.MarBrackish
MarBrackish.vs.Marine
100%
75%
50%
25%
0%
FwBrackish greater
Freshwater greater
Euryhaline greater
MarBrackish greater
Marine greater

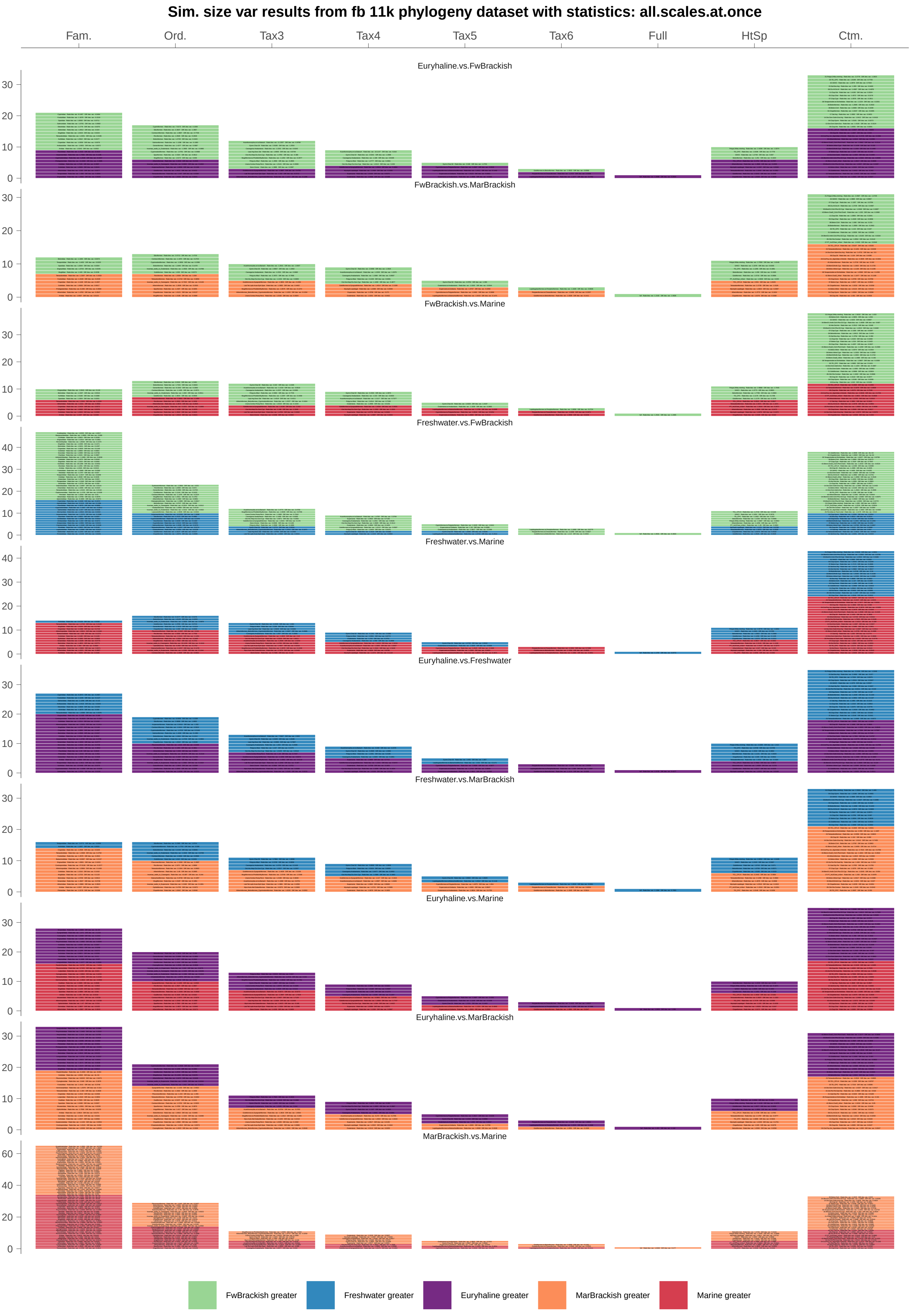

Sim. size var results from fb 11k phylogeny dataset with statistics: all.scales.at.once
Fam.
Ord.
Tax3
Tax4
Tax5
Tax6
Full
HtSp
Ctm.
Euryhaline.vs.FwBrackish
FwBrackish.vs.MarBrackish
FwBrackish.vs.Marine
Freshwater.vs.FwBrackish
Freshwater.vs.Marine
Euryhaline.vs.Freshwater
Freshwater.vs.MarBrackish
Euryhaline.vs.Marine
Euryhaline.vs.MarBrackish
MarBrackish.vs.Marine
FwBrackish greater
Freshwater greater
Euryhaline greater
MarBrackish greater
Marine greater

Size var rat. results from fb 11k phylogeny dataset: all.scales.at.once

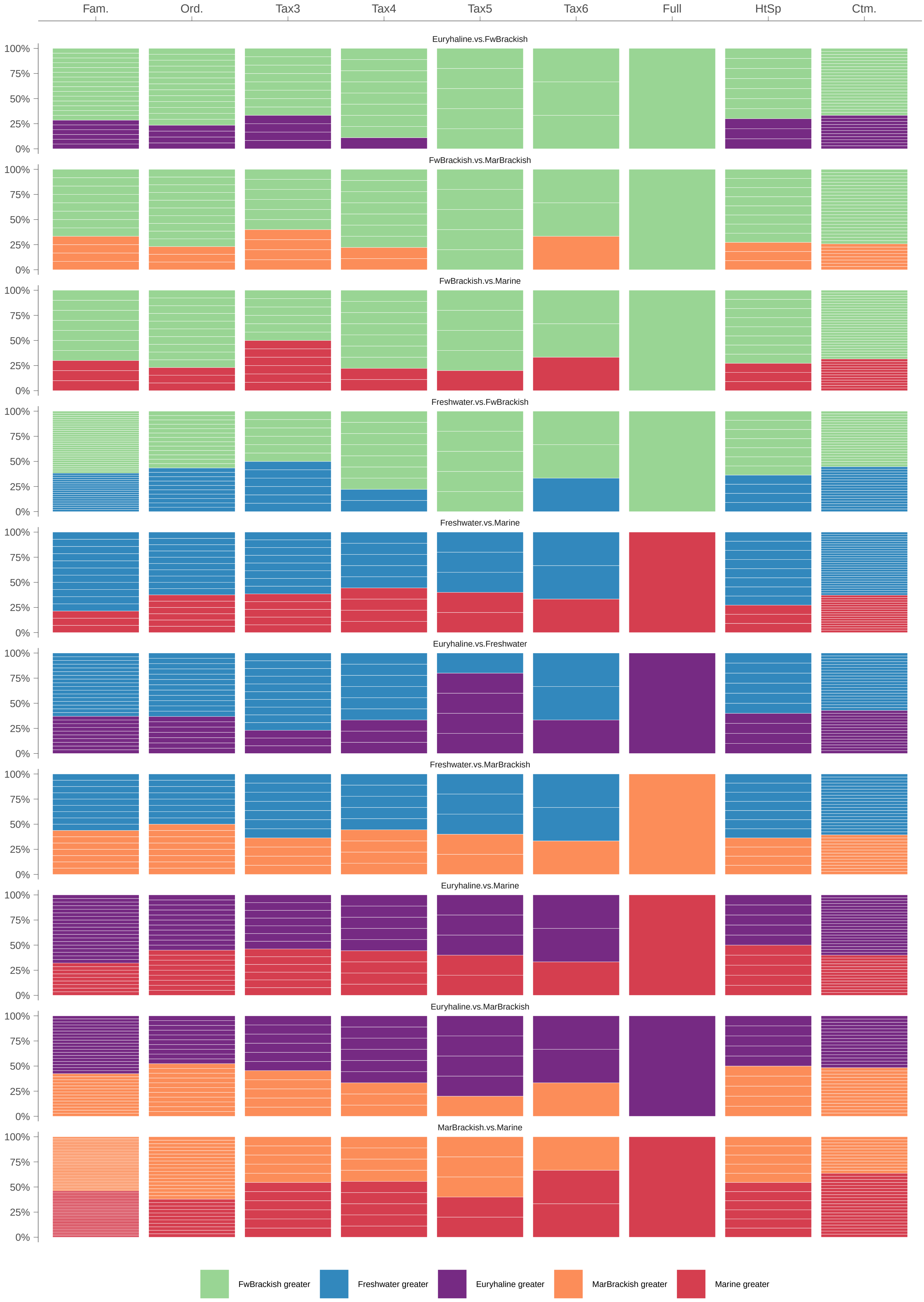

Size var rat. results from fb 11k phylogeny dataset with statistics: all.scales.at.once

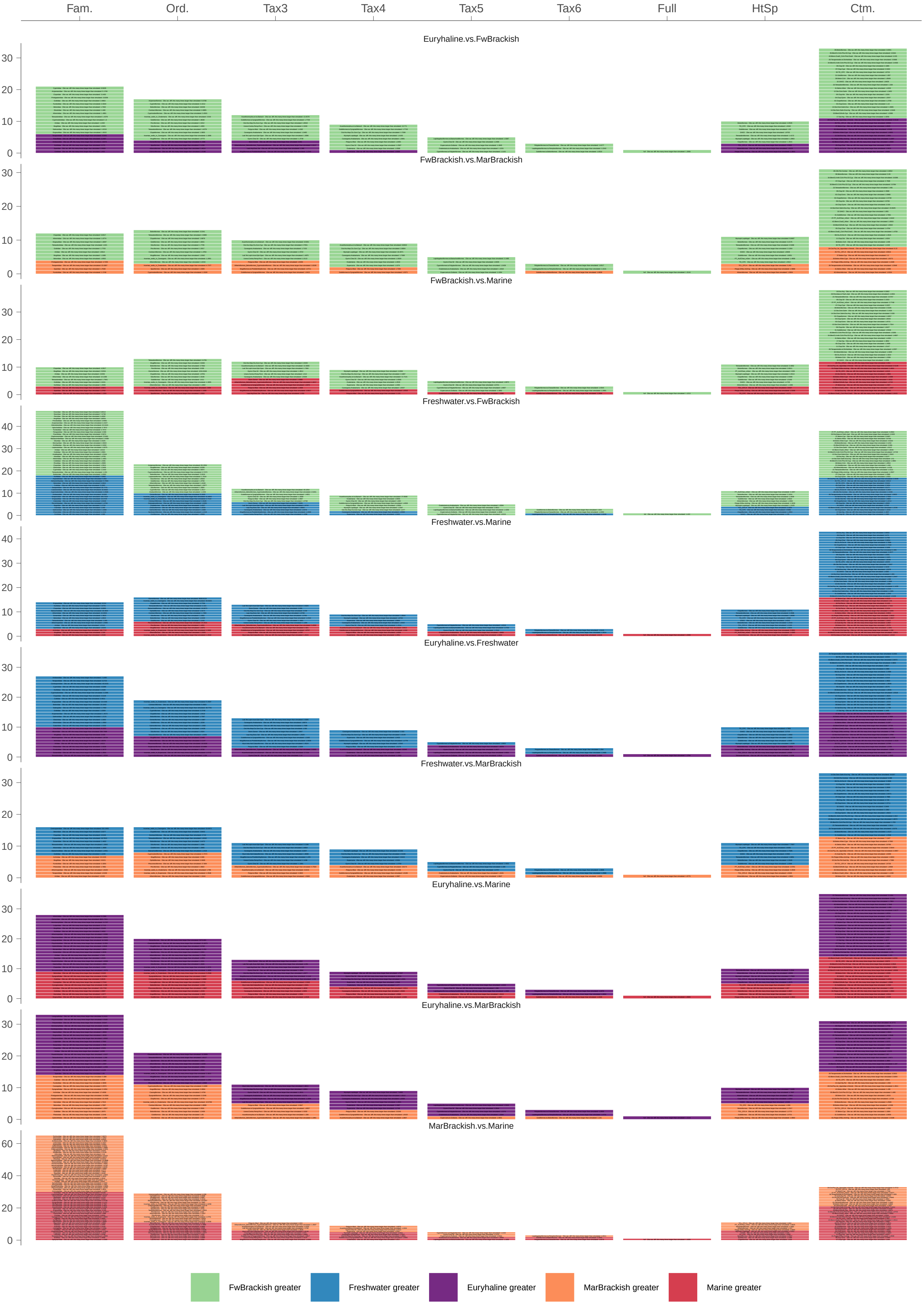

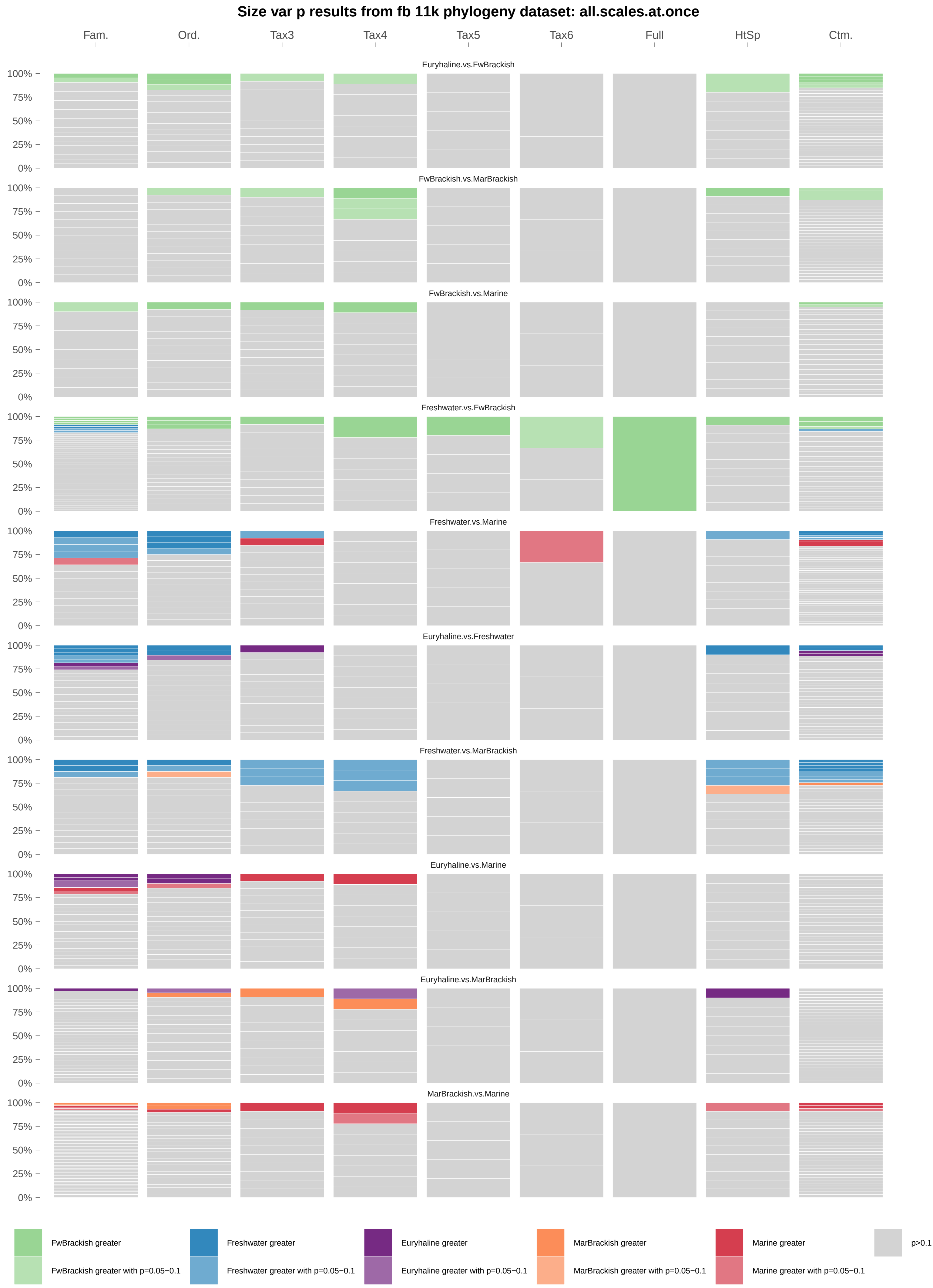

Size var p results from fb 11k phylogeny dataset: all.scales.at.once
Fam.
Ord.
Tax3
Tax4
Tax5
Tax6
Full
HtSp
Ctm.
Euryhaline.vs.FwBrackish
FwBrackish.vs.MarBrackish
FwBrackish.vs.Marine
Freshwater.vs.FwBrackish
Freshwater.vs.Marine
Euryhaline.vs.Freshwater
Freshwater.vs.MarBrackish
Euryhaline.vs.Marine
Euryhaline.vs.MarBrackish
MarBrackish.vs.Marine
100%
75%
50%
25%
0%
FwBrackish greater
FwBrackish greater with p=0.05−0.1
Freshwater greater
Freshwater greater with p=0.05−0.1
Euryhaline greater
Euryhaline greater with p=0.05−0.1
MarBrackish greater
MarBrackish greater with p=0.05−0.1
Marine greater
Marine greater with p=0.05−0.1
p>0.1

Size var p results from fb 11k phylogeny dataset with statistics: all.scales.at.once

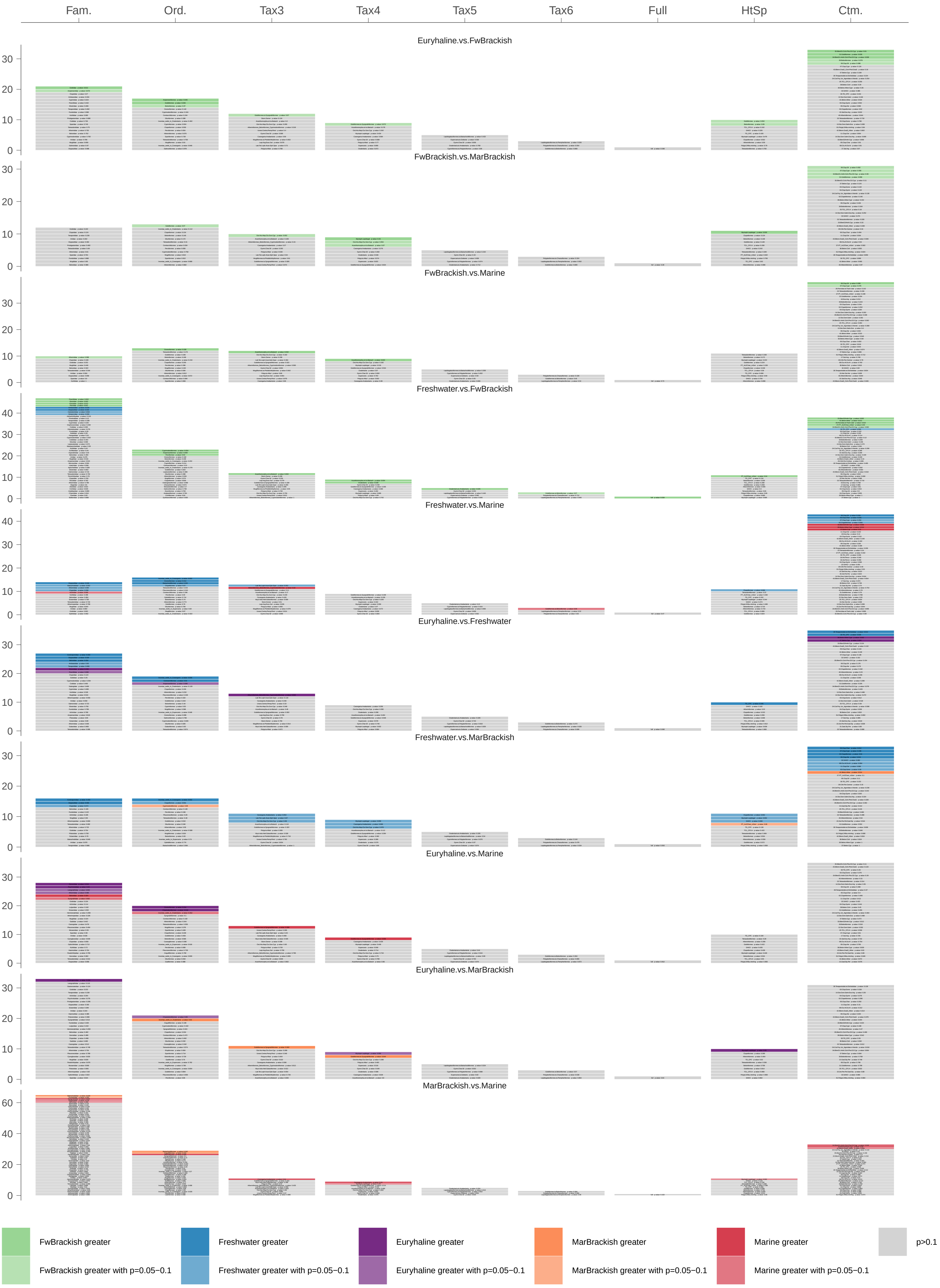

Supplement: Supplementary file 19 — Appendix 14 [file ELE-24-1569-s023.pdf]
