# Supplementary material for: Evidence for general size‐by‐habitat rules in actinopterygian fishes across nine scales of observation
Source: Ecol Lett. 2021 Jun 10;24(8):1569–81. doi: 10.1111/ele.13768 (PMC8362132; doi:10.1111/ele.13768)

Size var results from fb 31k phylogenies dataset: all.scales.at.once

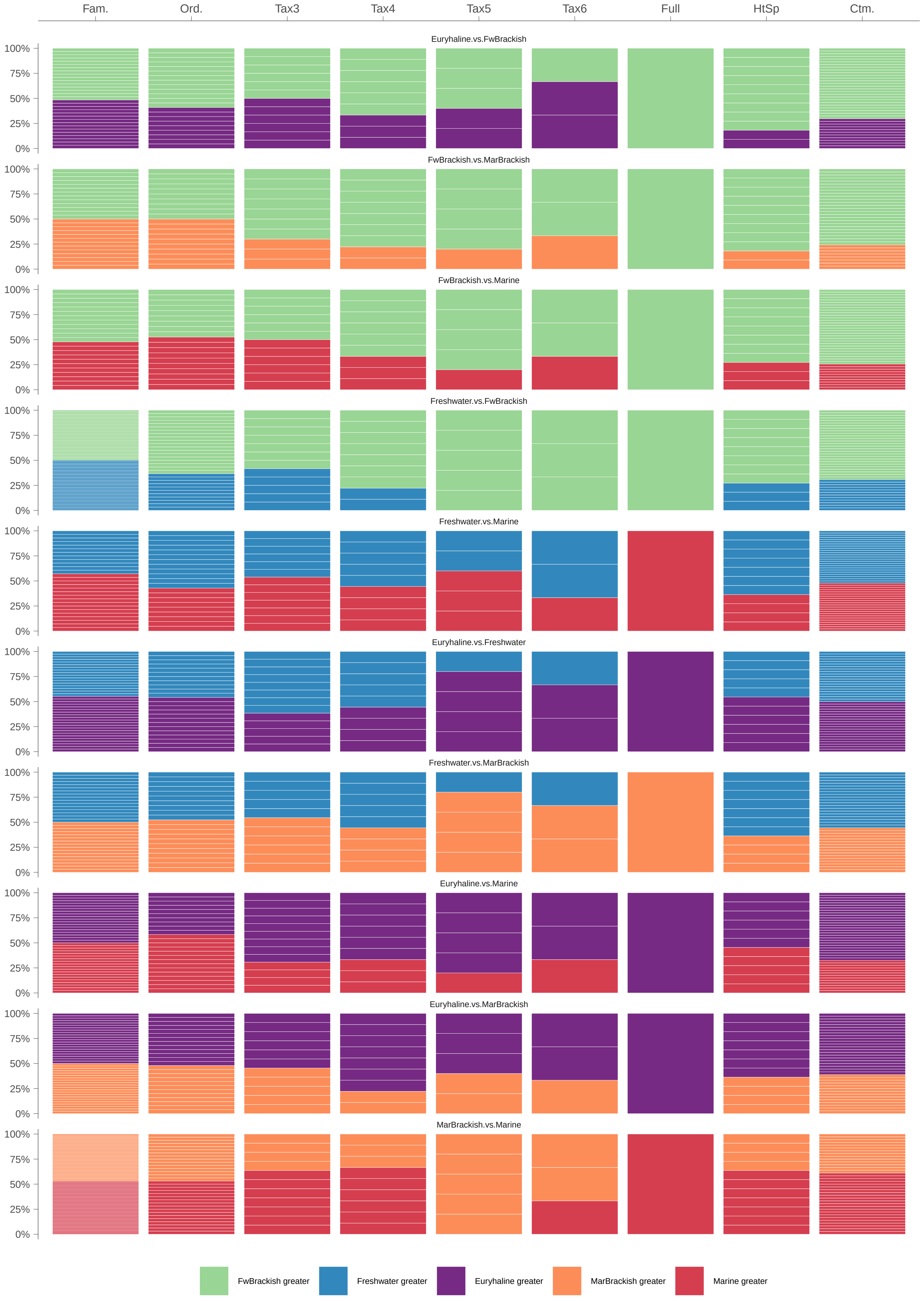

Size var results from fb 31k phylogenies dataset with statistics: all.scales.at.once

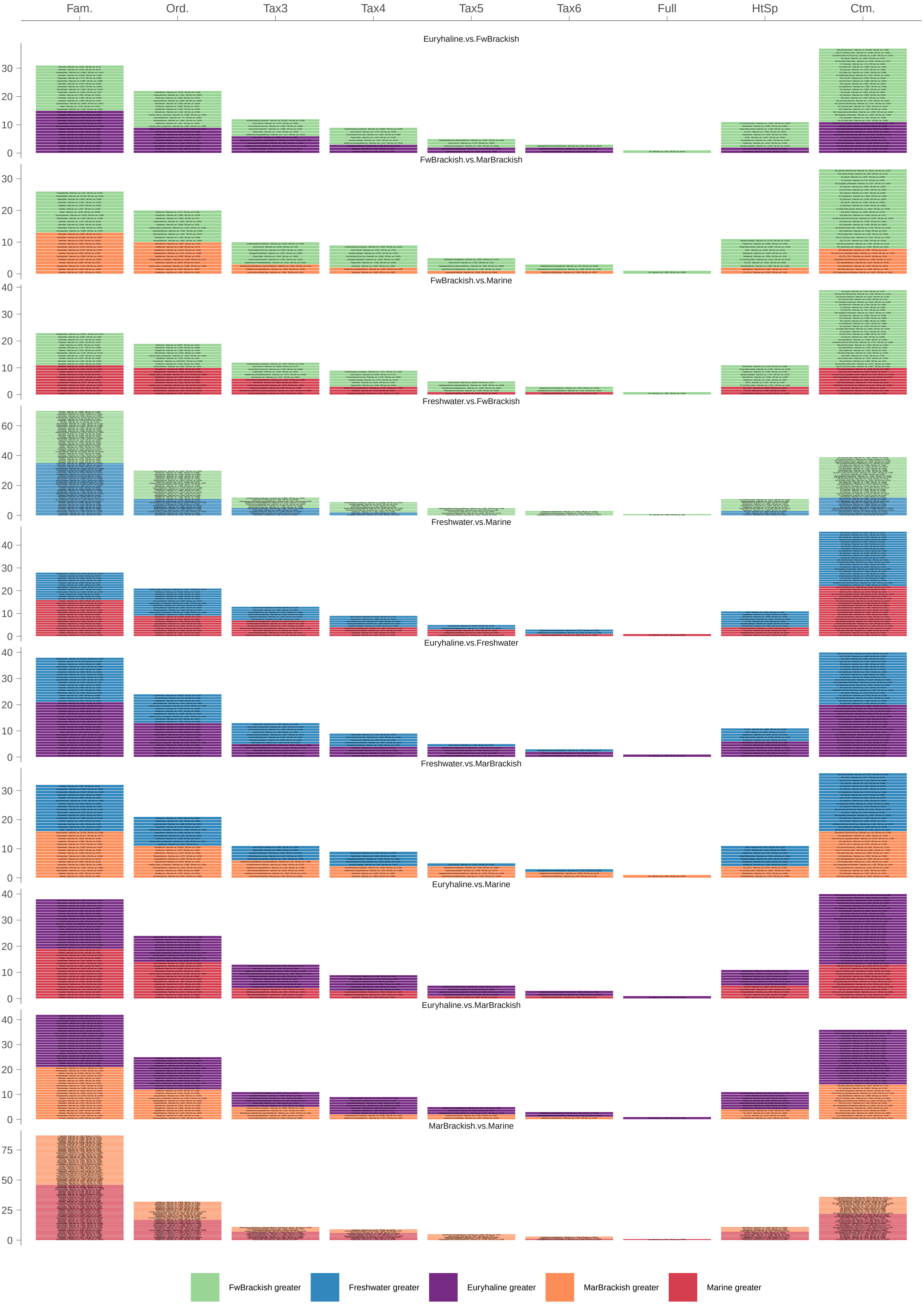

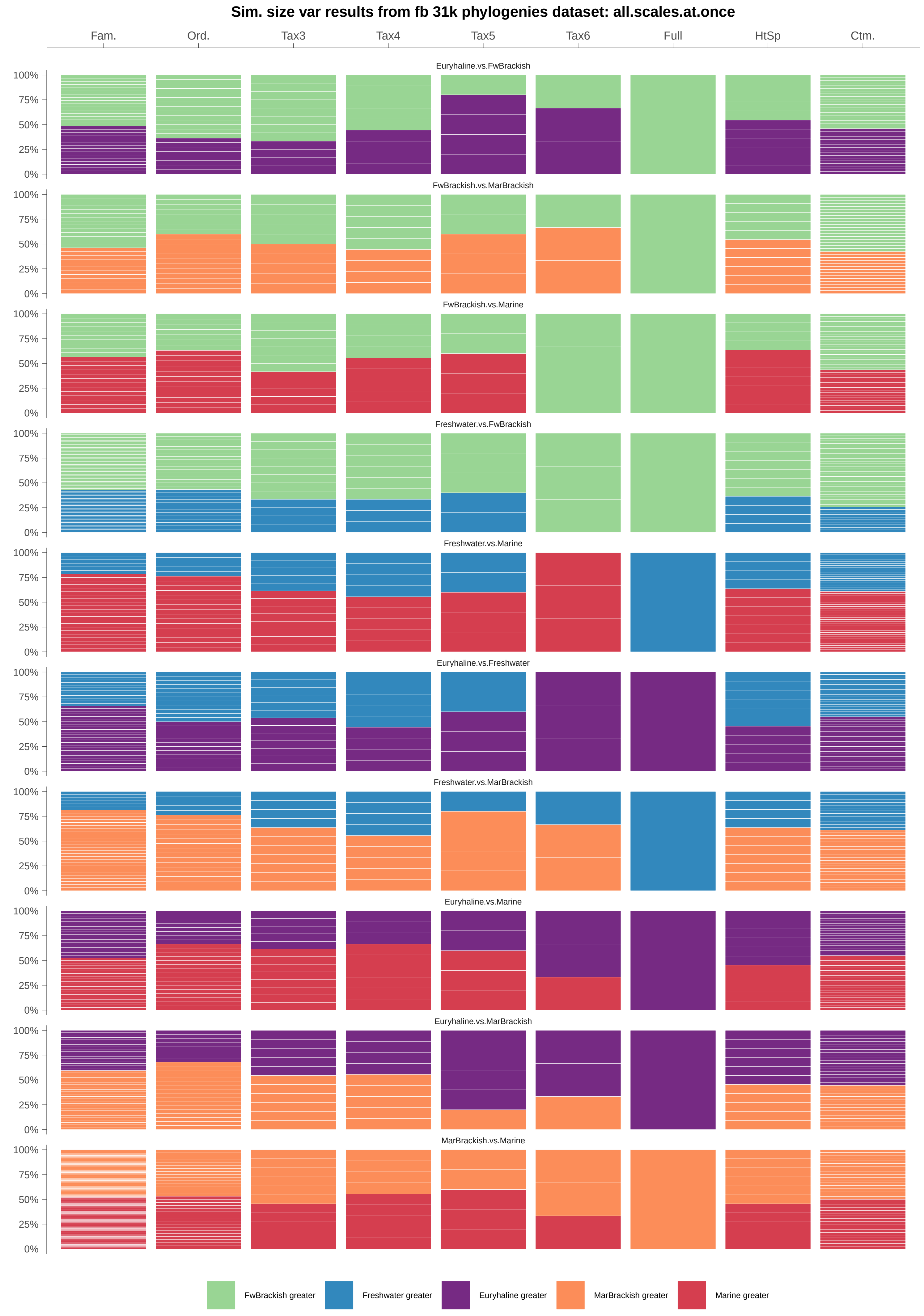
Sim. size var results from fb 31k phylogenies dataset: all.scales.at.once
Fam.
Ord.
Tax3
Tax4
Tax5
Tax6
Full
HtSp
Ctm.
Euryhaline.vs.FwBrackish
FwBrackish.vs.MarBrackish
FwBrackish.vs.Marine
Freshwater.vs.FwBrackish
Freshwater.vs.Marine
Euryhaline.vs.Freshwater
Freshwater.vs.MarBrackish
Euryhaline.vs.Marine
Euryhaline.vs.MarBrackish
MarBrackish.vs.Marine
100%
75%
50%
25%
0%
FwBrackish greater
Freshwater greater
Euryhaline greater
MarBrackish greater
Marine greater

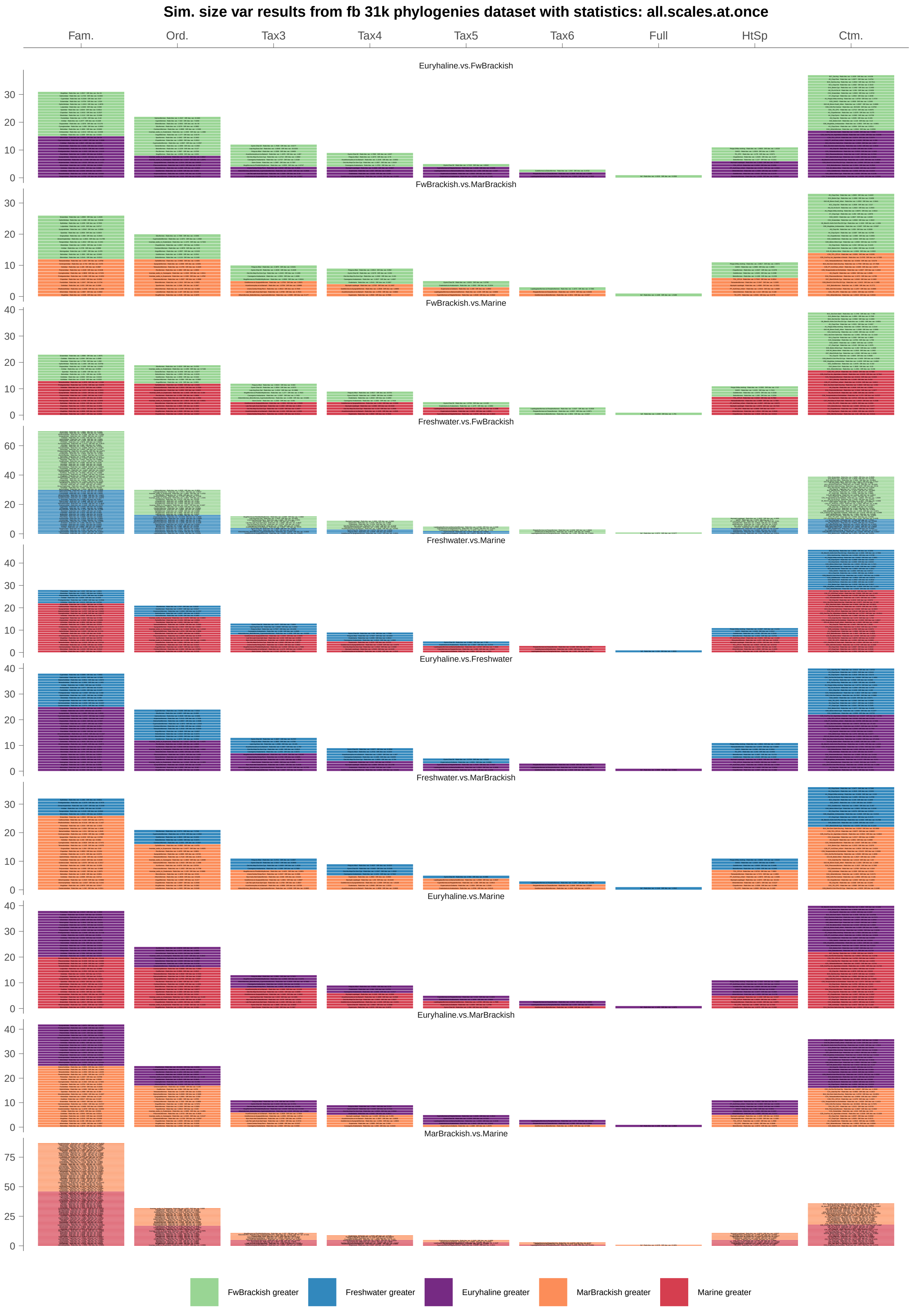
Sim. size var results from fb 31k phylogenies dataset with statistics: all.scales.at.once
Fam.
Ord.
Tax3
Tax4
Tax5
Tax6
Full
HtSp
Ctm.
Euryhaline.vs.FwBrackish
FwBrackish.vs.MarBrackish
FwBrackish.vs.Marine
Freshwater.vs.FwBrackish
Freshwater.vs.Marine
Euryhaline.vs.Freshwater
Freshwater.vs.MarBrackish
Euryhaline.vs.Marine
Euryhaline.vs.MarBrackish
MarBrackish.vs.Marine
FwBrackish greater
Freshwater greater
Euryhaline greater
MarBrackish greater
Marine greater

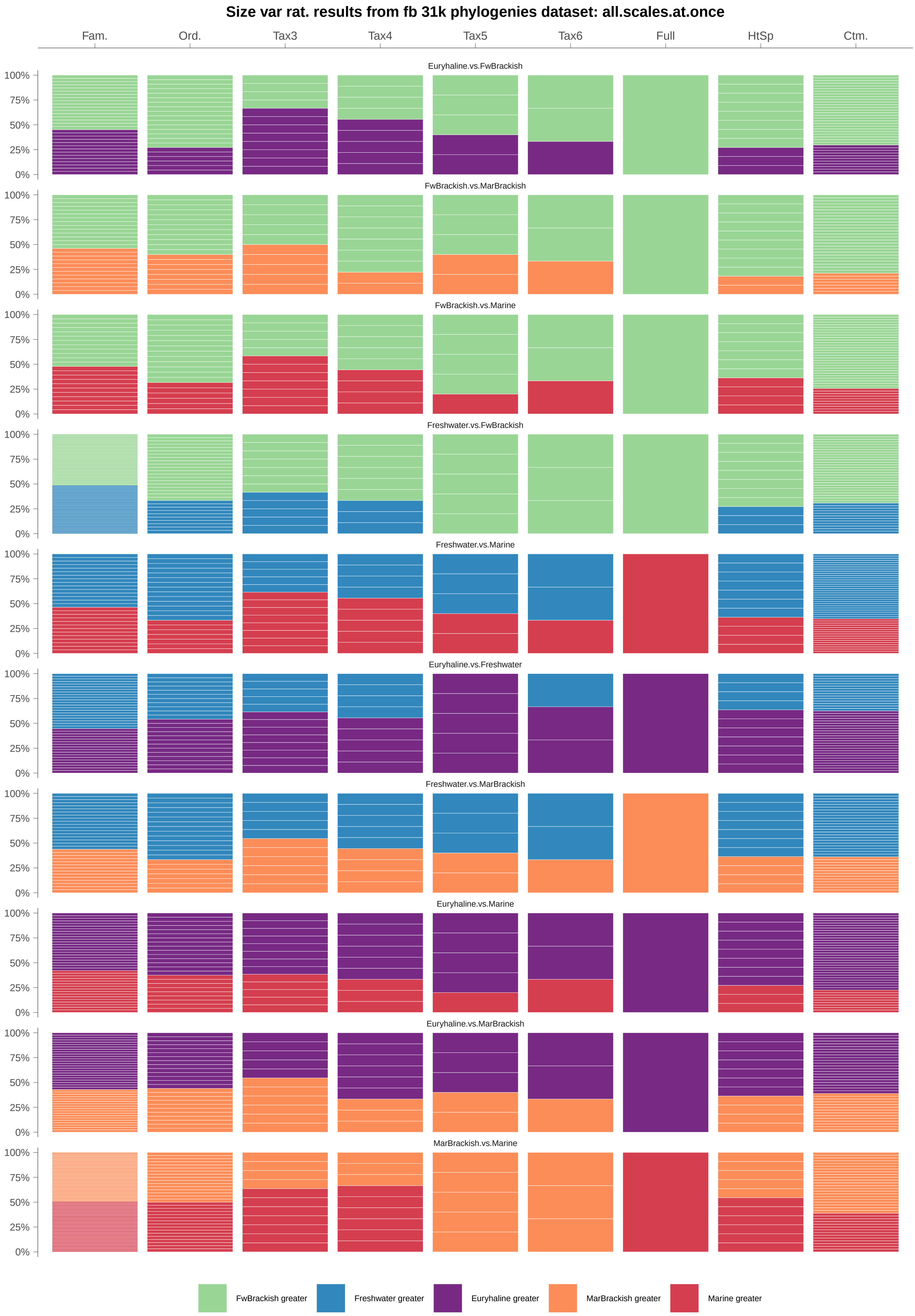

Size var rat. results from fb 31k phylogenies dataset: all.scales.at.once
Fam.
Ord.
Tax3
Tax4
Tax5
Tax6
Full
HtSp
Ctm.
Euryhaline.vs.FwBrackish
FwBrackish.vs.MarBrackish
FwBrackish.vs.Marine
Freshwater.vs.FwBrackish
Freshwater.vs.Marine
Euryhaline.vs.Freshwater
Freshwater.vs.MarBrackish
Euryhaline.vs.Marine
Euryhaline.vs.MarBrackish
MarBrackish.vs.Marine
100%
75%
50%
25%
0%
FwBrackish greater
Freshwater greater
Euryhaline greater
MarBrackish greater
Marine greater

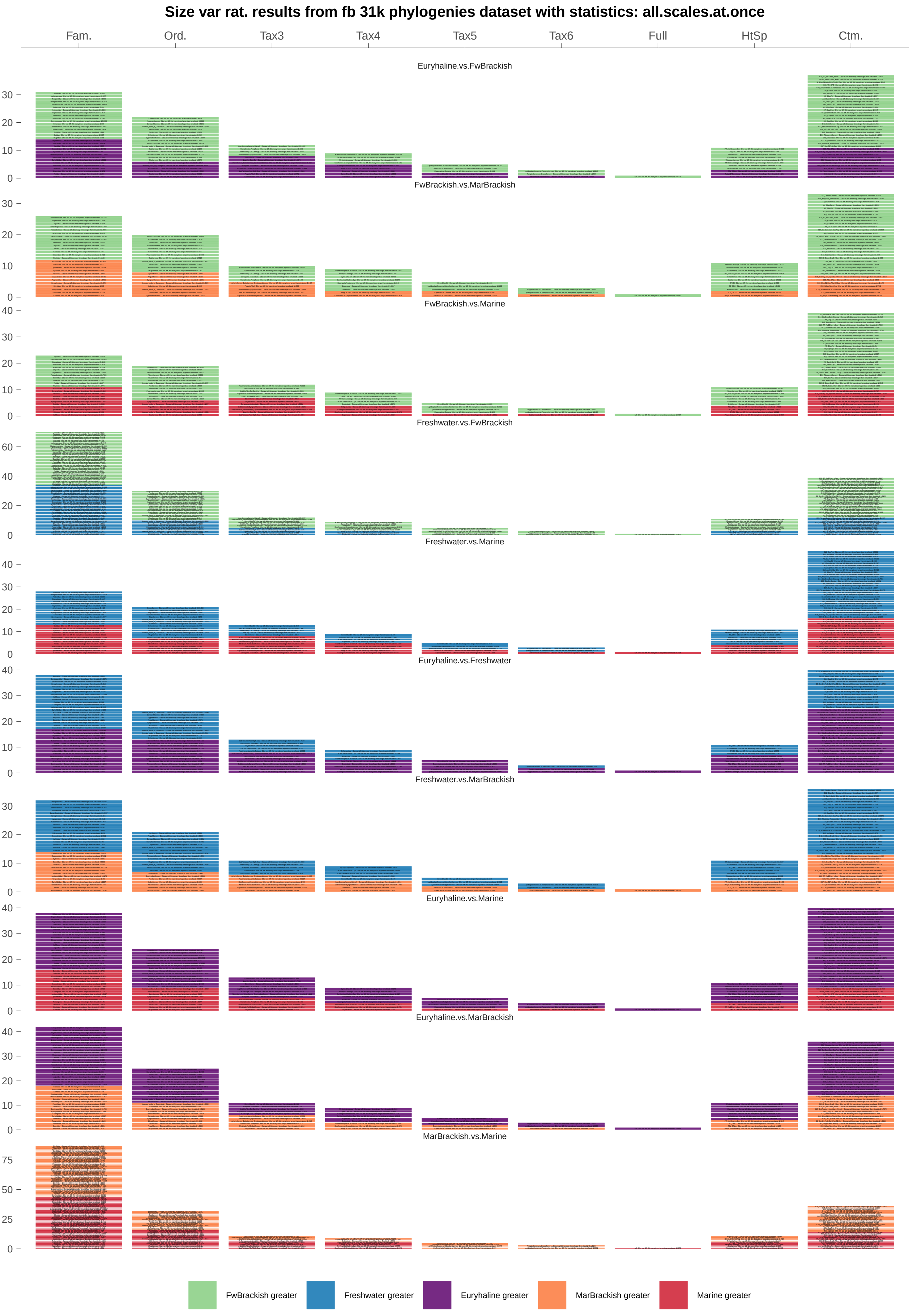
Size var rat. results from fb 31k phylogenies dataset with statistics: all.scales.at.once
Fam.
Ord.
Tax3
Tax4
Tax5
Tax6
Full
HtSp
Ctm.
Euryhaline.vs.FwBrackish
FwBrackish.vs.MarBrackish
FwBrackish.vs.Marine
Freshwater.vs.FwBrackish
Freshwater.vs.Marine
Euryhaline.vs.Freshwater
Freshwater.vs.MarBrackish
Euryhaline.vs.Marine
Euryhaline.vs.MarBrackish
MarBrackish.vs.Marine
FwBrackish greater
Freshwater greater
Euryhaline greater
MarBrackish greater
Marine greater

Size var p results from fb 31k phylogenies dataset: all.scales.at.once

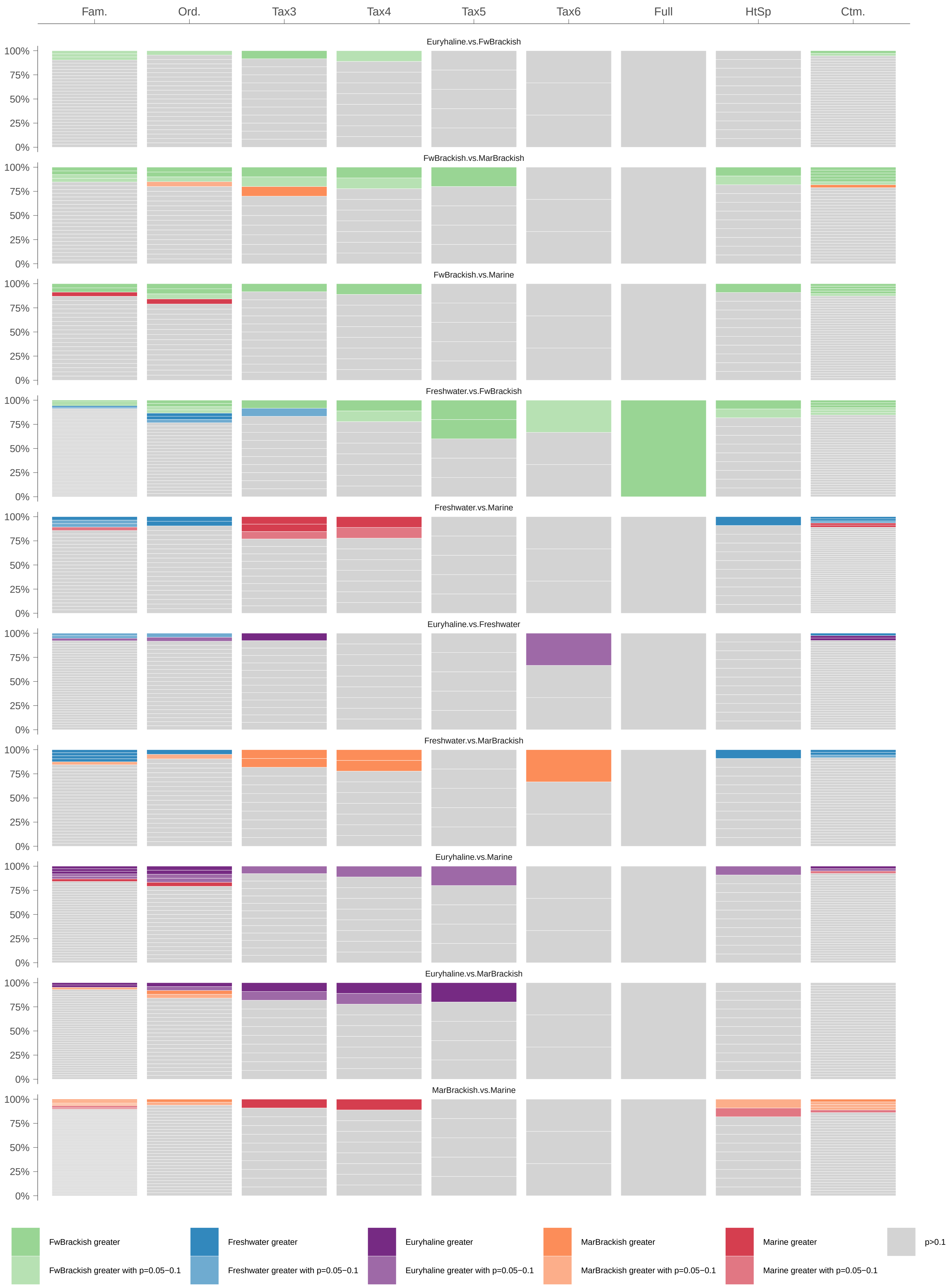

Size var p results from fb 31k phylogenies dataset with statistics: all.scales.at.once

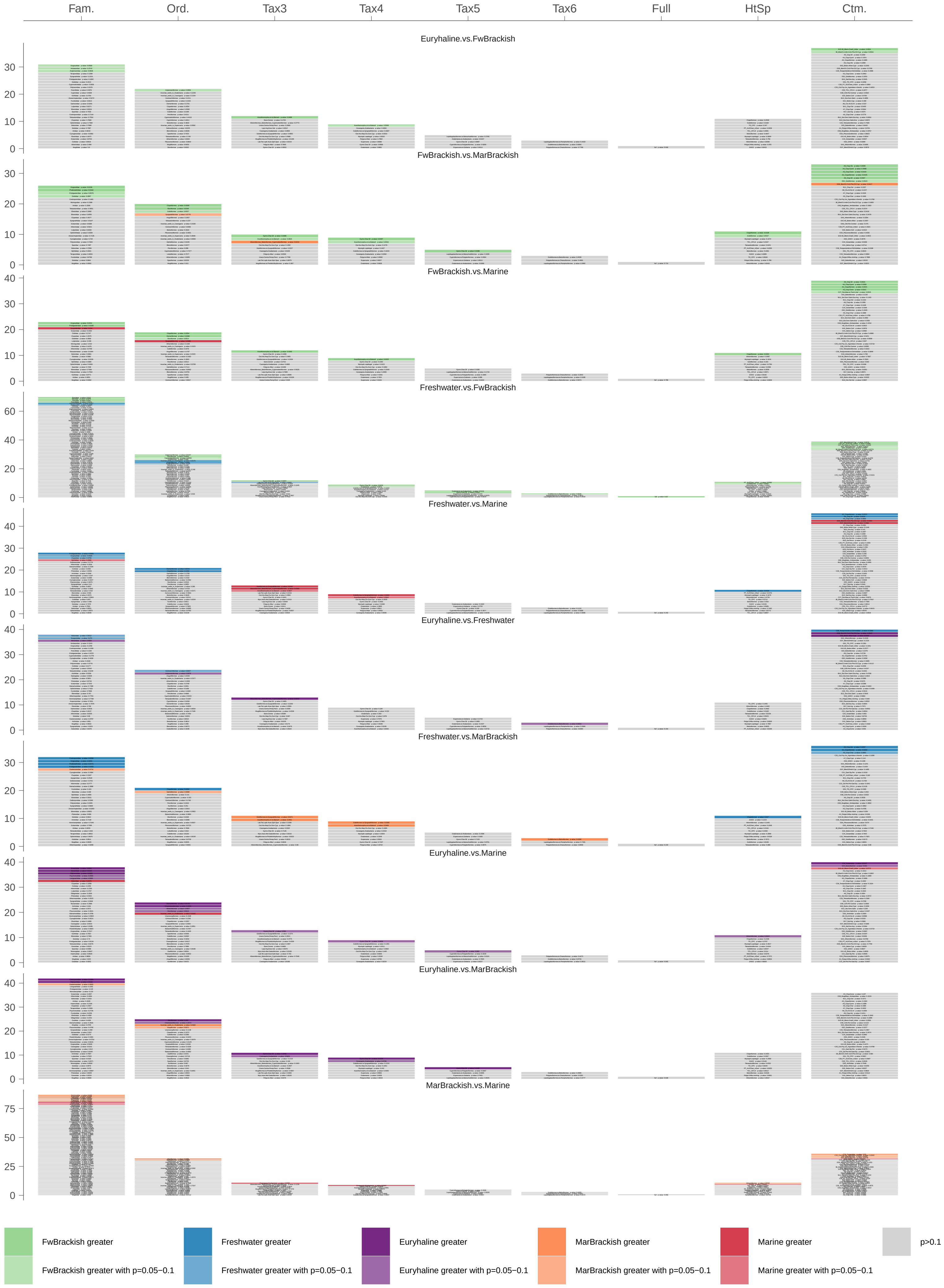

Supplement: Supplementary file 21 — Appendix 16 [file ELE-24-1569-s009.pdf]
