# Supplementary material for: Evidence for general size‐by‐habitat rules in actinopterygian fishes across nine scales of observation
Source: Ecol Lett. 2021 Jun 10;24(8):1569–81. doi: 10.1111/ele.13768 (PMC8362132; doi:10.1111/ele.13768)

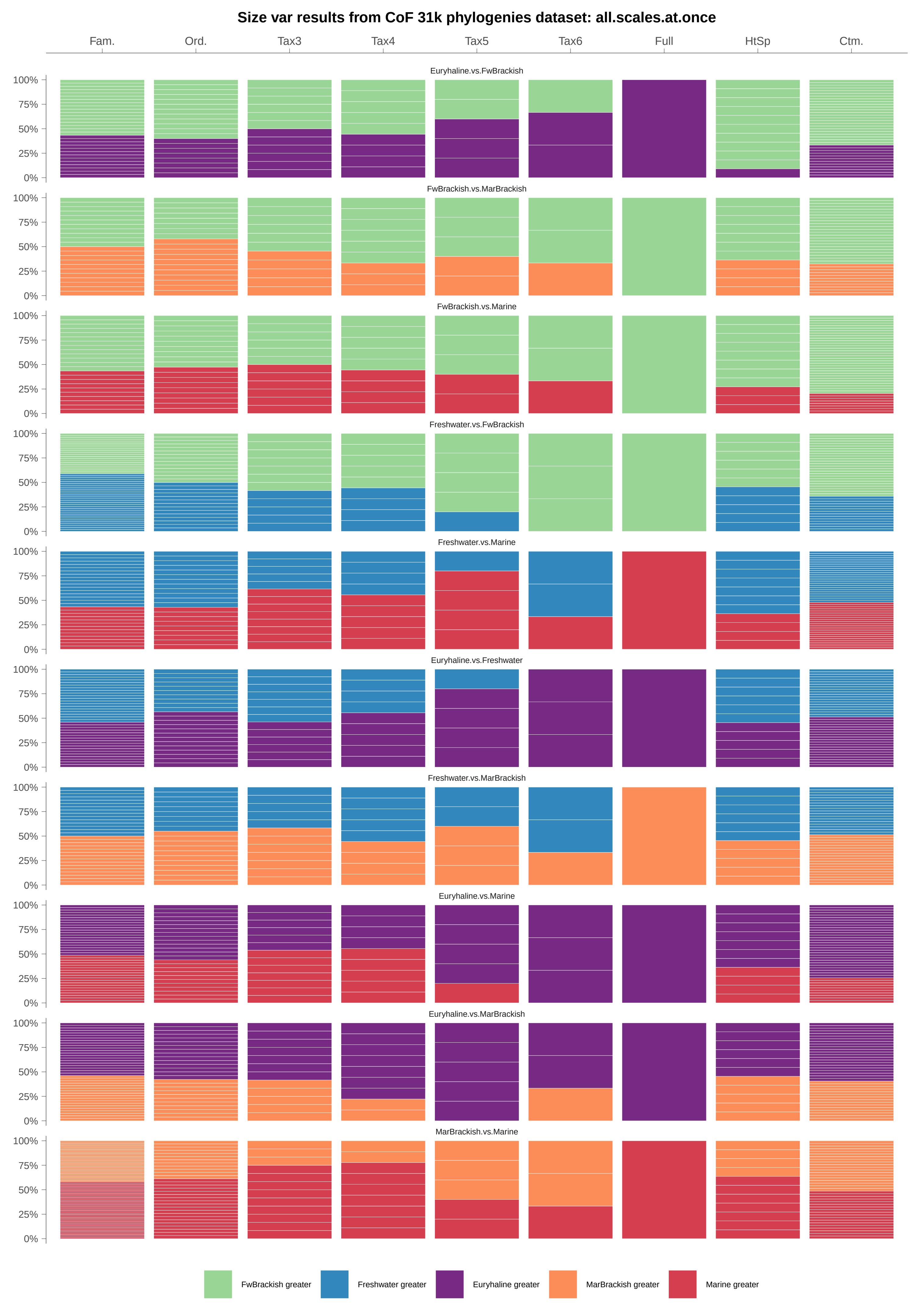
Size var results from CoF 31k phylogenies dataset: all.scales.at.once
Fam.
Ord.
Tax3
Tax4
Tax5
Tax6
Full
HtSp
Ctm.
Euryhaline.vs.FwBrackish
FwBrackish.vs.MarBrackish
FwBrackish.vs.Marine
Freshwater.vs.FwBrackish
Freshwater.vs.Marine
Euryhaline.vs.Freshwater
Freshwater.vs.MarBrackish
Euryhaline.vs.Marine
Euryhaline.vs.MarBrackish
MarBrackish.vs.Marine
100%
75%
50%
25%
0%
FwBrackish greater
Freshwater greater
Euryhaline greater
MarBrackish greater
Marine greater

Size var results from CoF 31k phylogenies dataset with statistics: all.scales.at.once

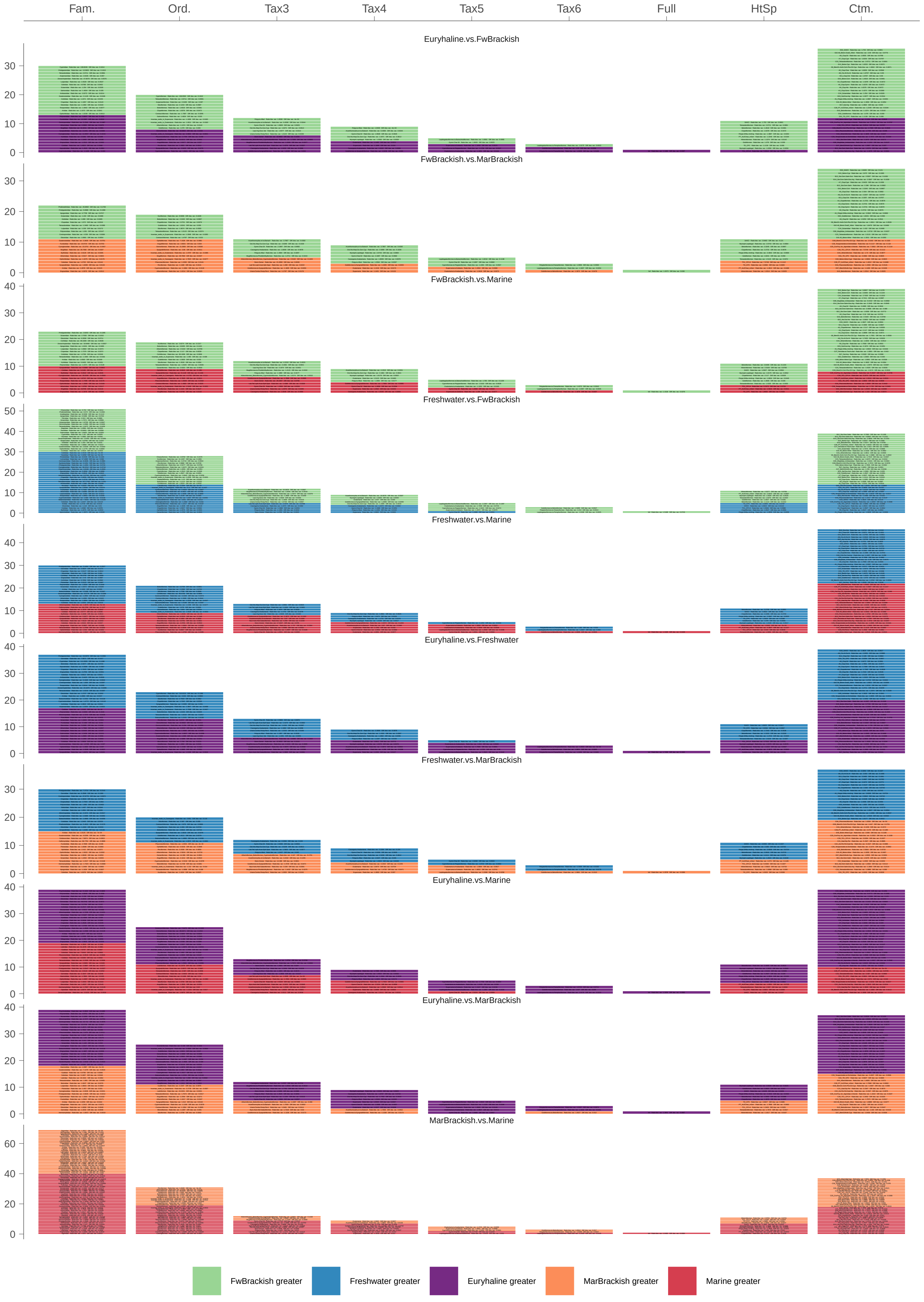

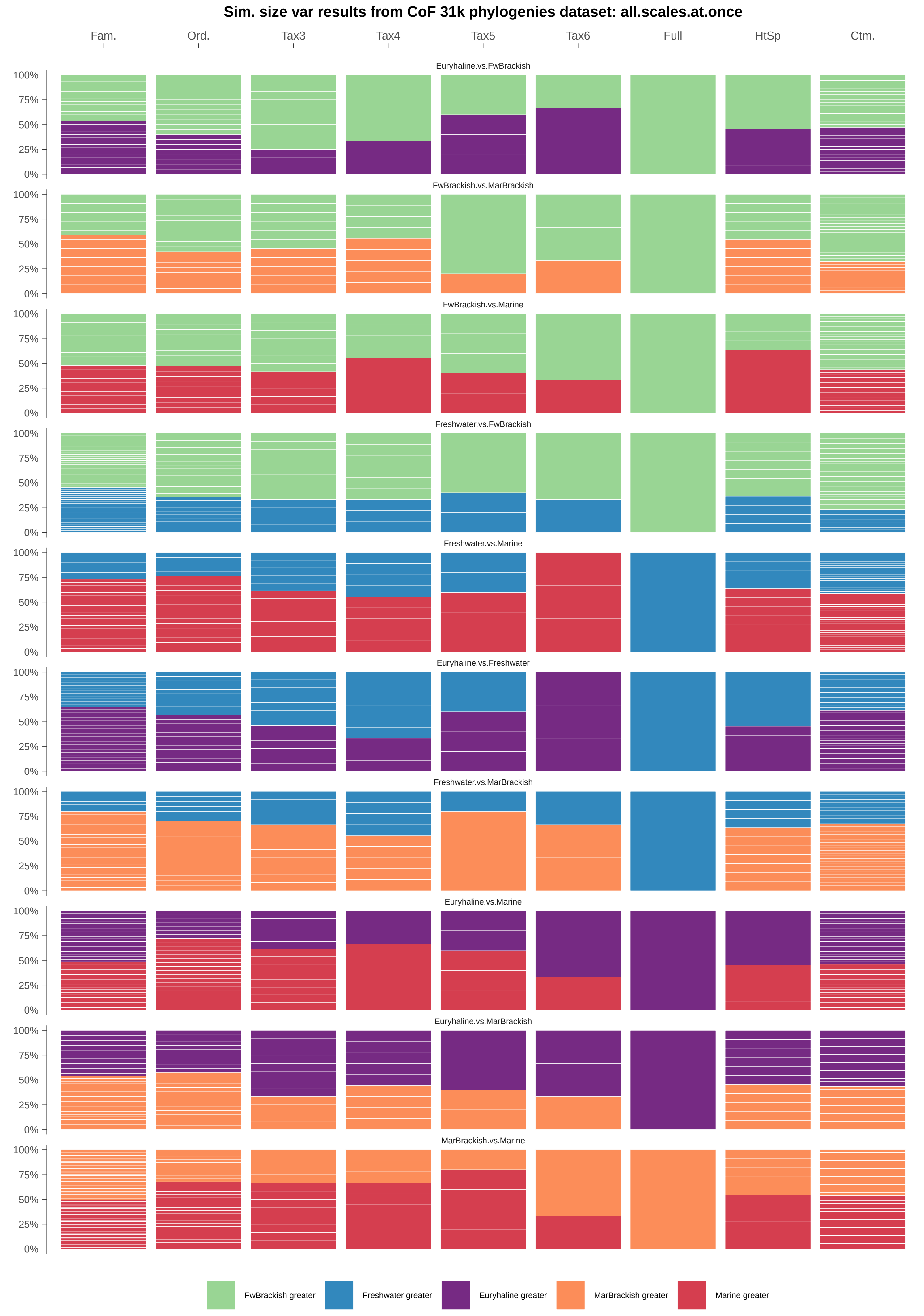

Sim. size var results from CoF 31k phylogenies dataset: all.scales.at.once
Fam.
Ord.
Tax3
Tax4
Tax5
Tax6
Full
HtSp
Ctm.
Euryhaline.vs.FwBrackish
FwBrackish.vs.MarBrackish
FwBrackish.vs.Marine
Freshwater.vs.FwBrackish
Freshwater.vs.Marine
Euryhaline.vs.Freshwater
Freshwater.vs.MarBrackish
Euryhaline.vs.Marine
Euryhaline.vs.MarBrackish
MarBrackish.vs.Marine
100%
75%
50%
25%
0%
FwBrackish greater
Freshwater greater
Euryhaline greater
MarBrackish greater
Marine greater

Sim. size var results from CoF 31k phylogenies dataset with statistics: all.scales.at.once

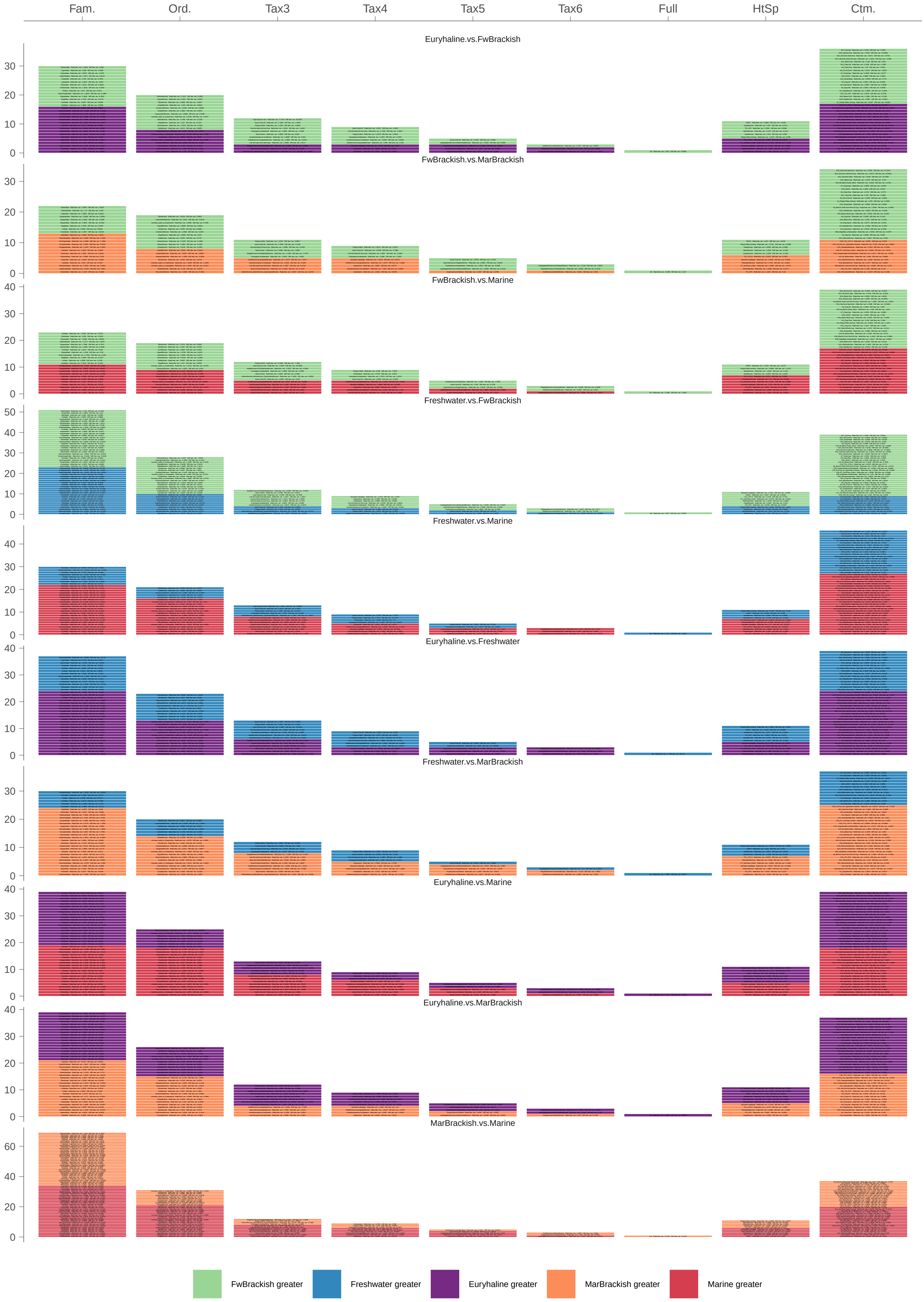

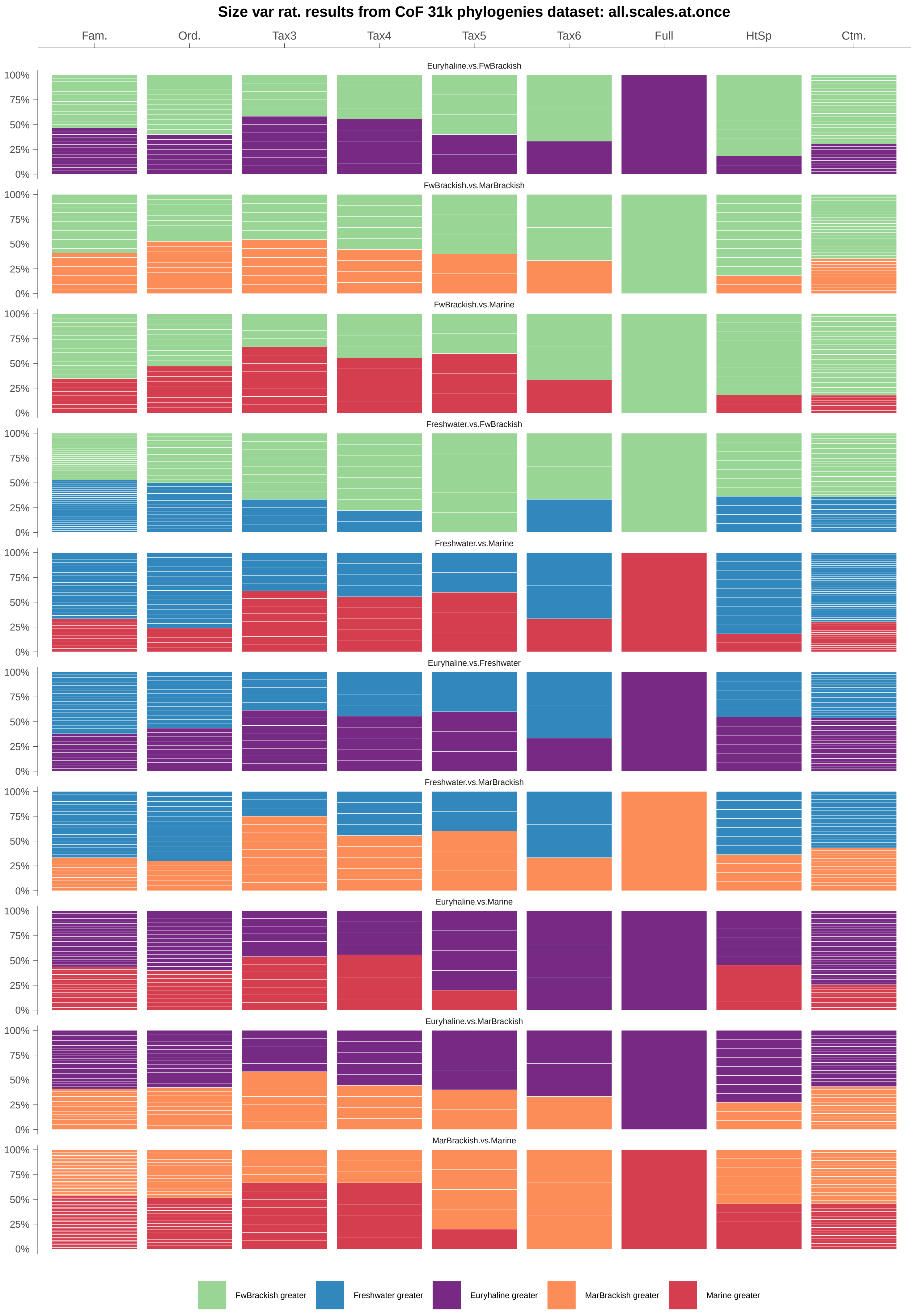
Size var rat. results from CoF 31k phylogenies dataset: all.scales.at.once
Fam.
Ord.
Tax3
Tax4
Tax5
Tax6
Full
HtSp
Ctm.
Euryhaline.vs.FwBrackish
FwBrackish.vs.MarBrackish
FwBrackish.vs.Marine
Freshwater.vs.FwBrackish
Freshwater.vs.Marine
Euryhaline.vs.Freshwater
Freshwater.vs.MarBrackish
Euryhaline.vs.Marine
Euryhaline.vs.MarBrackish
MarBrackish.vs.Marine
100%
75%
50%
25%
0%
FwBrackish greater
Freshwater greater
Euryhaline greater
MarBrackish greater
Marine greater

Size var rat. results from CoF 31k phylogenies dataset with statistics: all.scales.at.once

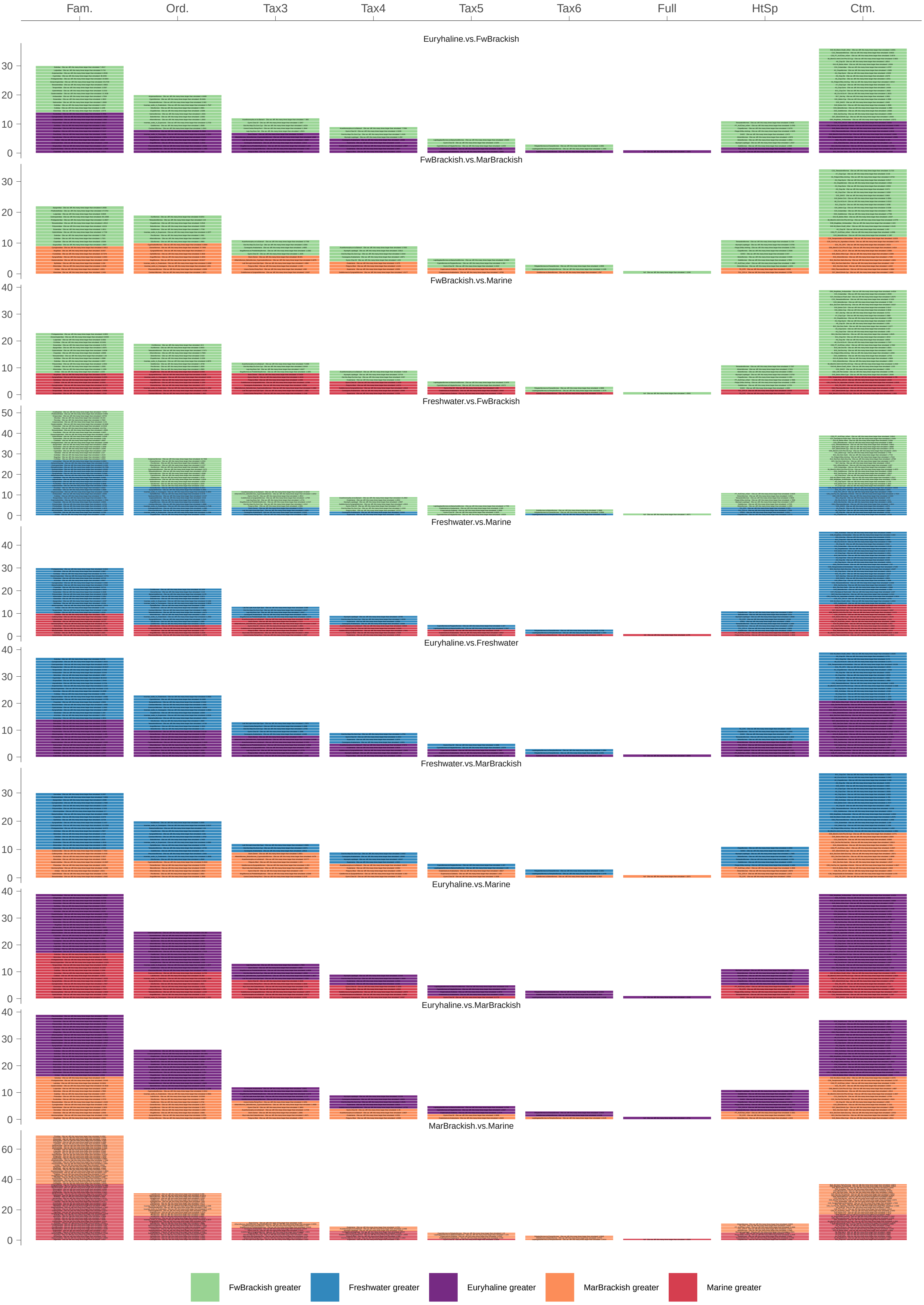

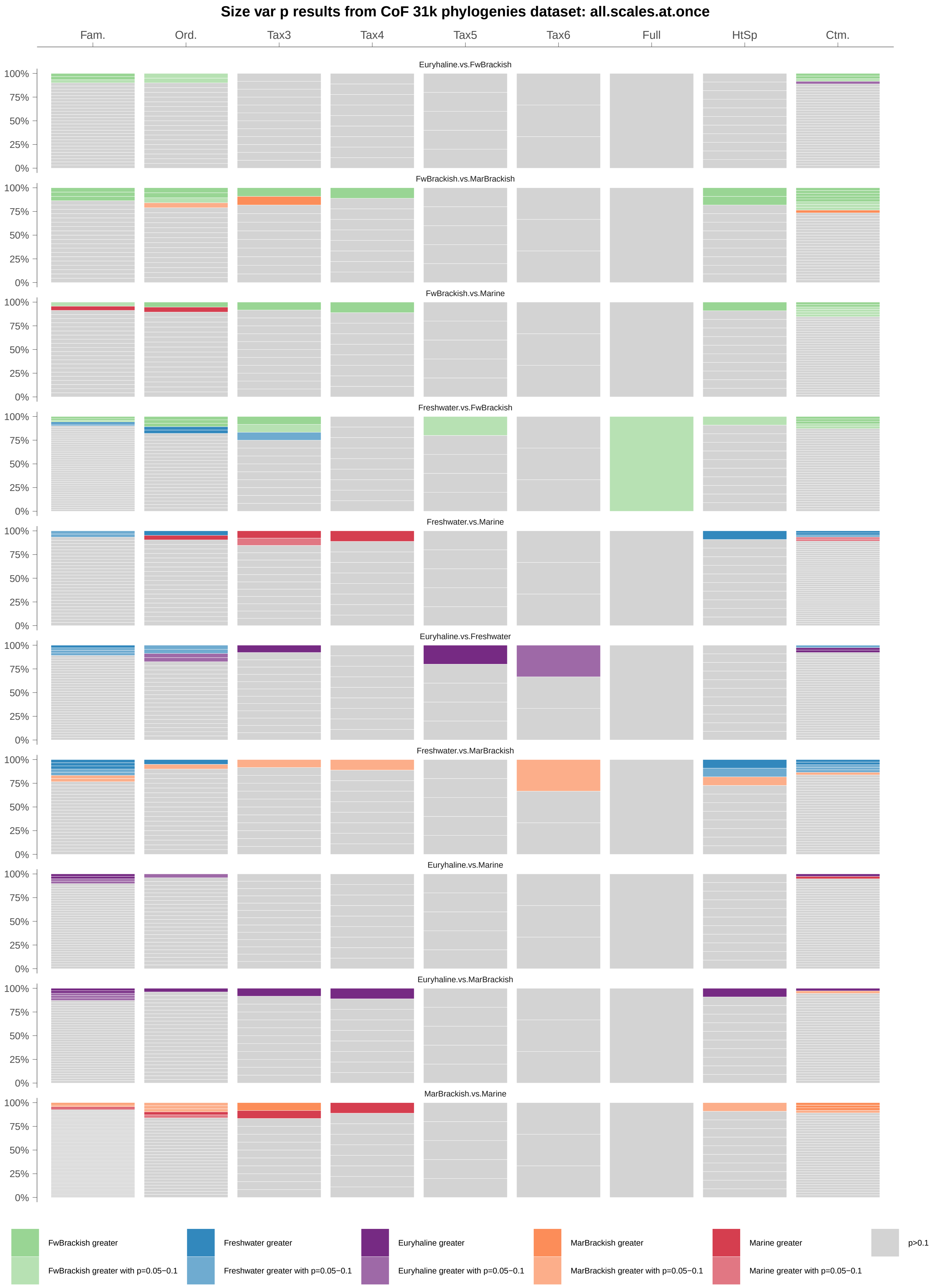
Size var p results from CoF 31k phylogenies dataset: all.scales.at.once
Fam.
Ord.
Tax3
Tax4
Tax5
Tax6
Full
HtSp
Ctm.
Euryhaline.vs.FwBrackish
FwBrackish.vs.MarBrackish
FwBrackish.vs.Marine
Freshwater.vs.FwBrackish
Freshwater.vs.Marine
Euryhaline.vs.Freshwater
Freshwater.vs.MarBrackish
Euryhaline.vs.Marine
Euryhaline.vs.MarBrackish
MarBrackish.vs.Marine
100%
75%
50%
25%
0%
FwBrackish greater
FwBrackish greater with p=0.05−0.1
Freshwater greater
Freshwater greater with p=0.05−0.1
Euryhaline greater
Euryhaline greater with p=0.05−0.1
MarBrackish greater
MarBrackish greater with p=0.05−0.1
Marine greater
Marine greater with p=0.05−0.1
p>0.1

Size var p results from CoF 31k phylogenies dataset with statistics: all.scales.at.once

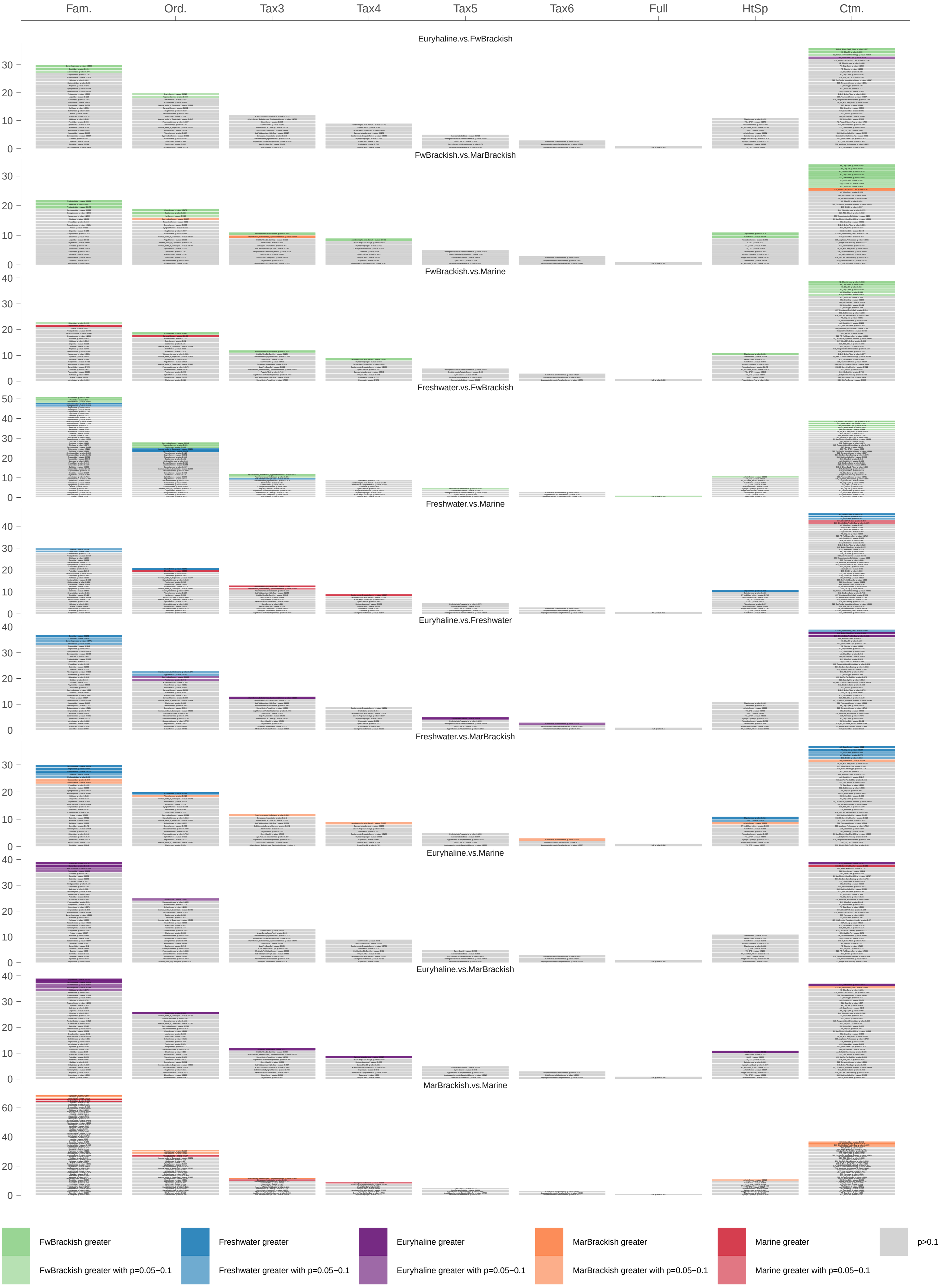

Supplement: Supplementary file 22 — Appendix 17 [file ELE-24-1569-s004.pdf]
