# Supplementary material for: Evidence for general size‐by‐habitat rules in actinopterygian fishes across nine scales of observation
Source: Ecol Lett. 2021 Jun 10;24(8):1569–81. doi: 10.1111/ele.13768 (PMC8362132; doi:10.1111/ele.13768)
Supplement: Supplementary file 23 [file ELE-24-1569-s014.pdf]

Appendix 1: The percentage of clades in which each pair of metrics, from the nine metrics compared between habitats, were aligned (e.g. the percentage of orders where euryhaline taxa possessed the smallest mean size and lower species richness, compared to freshwater relatives. An order where euryhaline taxa possessed the larger mean size and higher species richness also represents an alignment). I commonly refer to these as 'percentage alignments' of discrete outcomes. Clades whose metrics are aligned for a given habitat comparison fall within the white quadrants of Figure 4, while mismatched outcomes fall within grey quadrants.

Appendices 2 to 17 display all individual comparisons of size, trophic level, and size variance performed in the study. Grid cells in these plots contain statistical details, so please increase magnification on the pdfs to view these details.

These appendices provide full record of these results, so the reader can find an outcome for their clade of interest, with the data source, phylogeny, and analytical method they prefer.

Size results (largest possible dataset)
Comparisons of log10 body size using all taxa for which size data is available. Across the four datasets, the analyses represent a combined total of 5232 pairs of group + habitat comparisons (each of which were compared with five methods): The five methods are: 1. Observed log10 means; 2. Phylogenetic log10 means; 3. Wilcoxon test outcomes; 4. Simulation ANOVA test outcomes; 5. PGLS ANOVA test outcomes.

Appendix 2: All analyses pertaining to comparisons of taxon **size** between habitat-use types for the **FishBase 11k-tree dataset.** Plots indicate the percentage or absolute numbers of groups where the size of taxa within one habitat-use is greater than the other, obtained for every pairwise habitat-use comparison across multiple taxonomic scales. The five methods of size comparison (outlined in the Methods) are: i) Observed log10 means; ii) Phylogenetically corrected means, iii) Wilcoxon tests; iv) Simulation ANOVA; v) PGLS ANOVA. For p value outputs in methods iii–v), dark shades of each colour represent $p < 0.05$, lighter shades $p = 0.1–0.05$, and grey $p > 0.1$. For each approach, two figures are presented – the first emphasising the percentage of results that have fallen in a particular direction for each set of pairwise habitat comparisons within each taxonomic scale. The second figure is identical to the first with the exception that the y axis is free, so that absolute numbers of comparisons (not percentages) can be easily assessed. Statistical output for each comparison is printed within the relevant grid cell.

Appendix 3: All analyses pertaining to comparisons of taxon **size** between habitat-use types for the **Catalogue of Fishes 11k-tree dataset.** Plots indicate the percentage or absolute numbers of groups where the size of taxa within one habitat-use is greater than the other, obtained for every pairwise habitat-use comparison across multiple taxonomic scales. The five methods of size comparison (outlined in the Methods) are: i) Observed log10 means; ii) Phylogenetically corrected means, iii) Wilcoxon tests; iv) Simulation ANOVA; v) PGLS ANOVA. For p value outputs in methods iii–v), dark shades of each colour represent $p < 0.05$, lighter shades $p = 0.1 – 0.05$, and grey $p > 0.1$. For each approach, two figures are presented – the first emphasising the percentage of results that have fallen in a particular direction for each set of pairwise habitat comparisons within each taxonomic scale. The second figure is identical to the first with the exception that the y axis is free, so that absolute numbers of comparisons (not percentages) can be easily assessed. Statistical output for each comparison is printed within the relevant grid cell.

Appendix 4: All analyses pertaining to comparisons of taxon **size** between habitat-use types for the **FishBase 31k-tree dataset.** Plots indicate the percentage or absolute numbers of groups where the size of taxa within one habitat-use is greater than the other, obtained for every pairwise habitat-use comparison across multiple taxonomic scales. The five methods of size comparison (outlined in the Methods) are: i) Observed log10 means; ii) Phylogenetically corrected means, iii) Wilcoxon tests; iv) Simulation ANOVA; v) PGLS ANOVA. For p value outputs in methods iii–v), dark shades of each colour represent $p < 0.05$, lighter shades $p = 0.1–0.05$, and grey $p > 0.1$. For each approach, two

figures are presented – the first emphasising the percentage of results that have fallen in a particular direction for each set of pairwise habitat comparisons within each taxonomic scale. The second figure is identical to the first with the exception that the y axis is free, so that absolute numbers of comparisons (not percentages) can be easily assessed. Statistical output for each comparison is printed within the relevant grid cell.

Appendix 5: All analyses pertaining to comparisons of taxon **size** between habitat-use types for the **Catalogue of Fishes 31k-tree dataset.** Plots indicate the percentage or absolute numbers of groups where the size of taxa within one habitat-use is greater than the other, obtained for every pairwise habitat-use comparison across multiple taxonomic scales. The five methods of size comparison (outlined in the Methods) are: i) Observed means; ii) Phylogenetically corrected means, iii) Wilcoxon tests; iv) Simulation ANOVA; v) PGLS ANOVA. For p value outputs in methods iii-v), dark shades of each colour represent $p < 0.05$, lighter shades $p = 0.1–0.05$, and grey $p > 0.1$. For each approach, two figures are presented – the first emphasising the percentage of results that have fallen in a particular direction for each set of pairwise habitat comparisons within each taxonomic scale. The second figure is identical to the first with the exception that the y axis is free, so that absolute numbers of comparisons (not percentages) can be easily assessed. Statistical output for each comparison is printed within the relevant grid cell.

<u>Size results (reduced and retained size datasets, see Methods and SI text methods)</u>

Comparisons of log10 body size using all taxa in the reduced and retained size + trophic level datasets. Across the four datasets, the analyses represent a combined total of 3439 pairs of group + habitat comparisons (each of which were compared with five methods): The five methods are: 1. Observed log10 means; 2. Phylogenetic log10 means; 3. Wilcoxon test outcomes; 4. Simulation ANOVA test outcomes; 5. PGLS ANOVA test outcomes.

Appendix 6: All analyses pertaining to comparisons of taxon size (in the reduced and retained size datasets, see Methods) between habitat-use types for the **FishBase 11k-tree dataset. Trait "tSize" in plot titles therefore refers to the size dataset that was created in order to permit fair comparisons with trophic level, hence 't' in the trait name.** Plots indicate the percentage or absolute numbers of groups where the size of taxa within one habitat-use is greater than the other, obtained for every pairwise habitat-use comparison across multiple taxonomic scales. The five methods of size comparison (outlined in the Methods) are: i) Observed log10 means; ii) Phylogenetically corrected means, iii) Wilcoxon tests; iv) Simulation ANOVA; v) PGLS ANOVA. For p value outputs in methods iii–v), dark shades of each colour represent $p < 0.05$, lighter shades $p = 0.1–0.05$, and grey $p > 0.1$. For each approach, two figures are presented – the first emphasising the percentage of results that have fallen in a particular direction for each set of pairwise habitat comparisons within each taxonomic scale. The second figure is identical to the first with the exception that the y axis is free, so that absolute numbers of comparisons (not percentages) can be easily assessed. Statistical output for each comparison is printed within the relevant grid cell.

Appendix 7: All analyses pertaining to comparisons of taxon size (in the reduced and retained size datasets, see Methods) between habitat-use types for the **Catalogue of Fishes 11k-tree dataset. Trait "tSize" in plot titles therefore refers to the size dataset that was created in order to permit fair comparisons with trophic level, hence 't' in the trait name.** Plots indicate the percentage or absolute numbers of groups where the size of taxa within one habitat-use is greater than the other, obtained for every pairwise habitat-use comparison across multiple taxonomic scales. The five methods of size comparison (outlined in the Methods) are: i) Observed log10 means; ii) Phylogenetically corrected means, iii) Wilcoxon tests; iv) Simulation ANOVA; v) PGLS ANOVA. For p value outputs in methods iii–v), dark shades of each colour represent $p < 0.05$, lighter shades $p = 0.1 – 0.05$, and grey $p > 0.1$. For each approach, two figures are presented – the first emphasising the percentage of results that have fallen in a particular direction for each set of pairwise habitat comparisons within each taxonomic scale. The second figure is identical to the first with the exception that the y axis is free, so that absolute numbers of comparisons (not percentages) can be easily assessed. Statistical output for each comparison is printed within the relevant grid cell.

Appendix 8: All analyses pertaining to comparisons of taxon size (in the reduced and retained size datasets, see Methods) between habitat-use types for the **FishBase 31k-tree dataset. Trait "tSize" in plot titles therefore refers to the size dataset that was created in order to permit fair comparisons with trophic level, hence 't' in the trait name.** Plots indicate the percentage or absolute numbers of groups where the size of taxa within one habitat-use is greater than the other, obtained for every pairwise habitat-use comparison across multiple taxonomic scales. The five methods of size comparison (outlined in the Methods) are: i) Observed log10 means; ii) Phylogenetically corrected means, iii) Wilcoxon tests; iv) Simulation ANOVA; v) PGLS ANOVA. For p value outputs in methods iii–v), dark shades of each colour represent $p < 0.05$, lighter shades $p = 0.1–0.05$, and grey $p > 0.1$. For each approach, two figures are presented – the first emphasising the percentage of results that have fallen in a particular direction for each set of pairwise habitat comparisons within each taxonomic scale. The second figure is identical to the first with the exception that the y axis is free, so that absolute numbers of comparisons (not percentages) can be easily assessed. Statistical output for each comparison is printed within the relevant grid cell.

Appendix 9: All analyses pertaining to comparisons of taxon size (in the reduced and retained size datasets, see Methods) between habitat-use types for the **Catalogue of Fishes 31k-tree dataset. Trait "tSize" in plot titles therefore refers to the size dataset that was created in order to permit fair comparisons with trophic level, hence 't' in the trait name.** Plots indicate the percentage or absolute numbers of groups where the size of taxa within one habitat-use is greater than the other, obtained for every pairwise habitat-use comparison across multiple taxonomic scales. The five methods of size comparison (outlined in the Methods) are: i) Observed log10 means; ii) Phylogenetically corrected means, iii) Wilcoxon tests; iv) Simulation ANOVA; v) PGLS ANOVA. For p value outputs in methods iii-v), dark shades of each colour represent $p < 0.05$, lighter shades $p = 0.1–0.05$, and grey $p > 0.1$. For each approach, two figures are presented – the first emphasising the percentage of results that have fallen in a particular direction for each set of pairwise habitat comparisons within each taxonomic scale. The second figure is identical to the first with the exception that the y axis is free, so that absolute numbers of comparisons (not percentages) can be easily assessed. Statistical output for each comparison is printed within the relevant grid cell.

<u>Trophic level results (reduced and retained size datasets, see Methods and SI text methods)</u>

Comparisons of log10 trophic level using all taxa in the reduced and retained size + trophic level datasets. Across the four datasets, the analyses represent a combined total of 3439 pairs of group + habitat comparisons (each of which were compared with five methods): The five methods are: 1. Observed log10 means; 2. Phylogenetic log10 means; 3. Wilcoxon test outcomes; 4. Simulation ANOVA test outcomes; 5. PGLS ANOVA test outcomes.

Appendix 10: All analyses pertaining to comparisons of taxon **trophic level** (in the reduced and retained size datasets, see Methods) between habitat-use types for the **FishBase 11k-tree dataset.** Plots indicate the percentage or absolute numbers of groups where the trophic level of taxa within one habitat-use is greater than the other, obtained for every pairwise habitat-use comparison across multiple taxonomic scales. The five methods of trophic level comparison (outlined in the Methods) are: i) Observed log10 means; ii) Phylogenetically corrected means, iii) Wilcoxon tests; iv) Simulation ANOVA; v) PGLS ANOVA. For p value outputs in methods iii–v), dark shades of each colour represent $p < 0.05$, lighter shades $p = 0.1–0.05$, and grey $p > 0.1$. For each approach, two figures are presented – the first emphasising the percentage of results that have fallen in a particular direction for each set of pairwise habitat comparisons within each taxonomic scale. The second figure is identical to the first with the exception that the y axis is free, so that absolute numbers of comparisons (not percentages) can be easily assessed. Statistical output for each comparison is printed within the relevant grid cell.

Appendix 11: All analyses pertaining to comparisons of taxon **trophic level** (in the reduced and retained size datasets, see Methods) between habitat-use types for the **Catalogue of Fishes 11k-tree dataset.** Plots indicate the

percentage or absolute numbers of groups where the trophic level of taxa within one habitat-use is greater than the other, obtained for every pairwise habitat-use comparison across multiple taxonomic scales. The five methods of trophic level comparison (outlined in the Methods) are: i) Observed log10 means; ii) Phylogenetically corrected means, iii) Wilcoxon tests; iv) Simulation ANOVA; v) PGLS ANOVA. For p value outputs in methods iii–v), dark shades of each colour represent p < 0.05, lighter shades p = 0.1–0.05, and grey p > 0.1. For each approach, two figures are presented – the first emphasising the percentage of results that have fallen in a particular direction for each set of pairwise habitat comparisons within each taxonomic scale. The second figure is identical to the first with the exception that the y axis is free, so that absolute numbers of comparisons (not percentages) can be easily assessed. Statistical output for each comparison is printed within the relevant grid cell.

Appendix 12: All analyses pertaining to comparisons of taxon **trophic level** (in the reduced and retained size datasets, see Methods) between habitat-use types for the **FishBase 31k-tree dataset.** Plots indicate the percentage or absolute numbers of groups where the trophic level of taxa within one habitat-use is greater than the other, obtained for every pairwise habitat-use comparison across multiple taxonomic scales. The five methods of trophic level comparison (outlined in the Methods) are: i) Observed log10 means; ii) Phylogenetically corrected means, iii) Wilcoxon tests; iv) Simulation ANOVA; v) PGLS ANOVA. For p value outputs in methods iii–v), dark shades of each colour represent p < 0.05, lighter shades p = 0.1–0.05, and grey p > 0.1. For each approach, two figures are presented – the first emphasising the percentage of results that have fallen in a particular direction for each set of pairwise habitat comparisons within each taxonomic scale. The second figure is identical to the first with the exception that the y axis is free, so that absolute numbers of comparisons (not percentages) can be easily assessed. Statistical output for each comparison is printed within the relevant grid cell.

Appendix 13: All analyses pertaining to comparisons of taxon **trophic level** (in the reduced and retained size datasets, see Methods) between habitat-use types for the **Catalogue of Fishes 31k-tree dataset.** Plots indicate the percentage or absolute numbers of groups where the trophic level of taxa within one habitat-use is greater than the other, obtained for every pairwise habitat-use comparison across multiple taxonomic scales. The five methods of trophic level comparison (outlined in the Methods) are: i) Observed means; ii) Phylogenetically corrected means, iii) Wilcoxon tests; iv) Simulation ANOVA; v) PGLS ANOVA. For p value outputs in methods iii-v), dark shades of each colour represent p < 0.05, lighter shades p = 0.1–0.05, and grey p > 0.1. For each approach, two figures are presented – the first emphasising the percentage of results that have fallen in a particular direction for each set of pairwise habitat comparisons within each taxonomic scale. The second figure is identical to the first with the exception that the y axis is free, so that absolute numbers of comparisons (not percentages) can be easily assessed. Statistical output for each comparison is printed within the relevant grid cell.

<u>Size variance results (largest possible dataset)</u>

Comparisons of log10 body size variance using all taxa for which size data is available. Across the four datasets, the analyses represent a combined total of 5232 pairs of group + habitat comparisons (each of which were compared with four methods): The four methods are: 1. Observed log10 variance; 2. Expected log10 variance from simulations; 3. Observed variance vs. simulated variance; 4. P values derived from observed variance vs. simulated variance.

Appendix 14: All analyses pertaining to comparisons of **size variance** between habitat-use types for the **FishBase 11k-tree dataset.** Plots indicate the percentage or absolute numbers of groups where the size variance of taxa within one habitat-use is greater than the other, obtained for every pairwise habitat-use comparison across multiple taxonomic scales. The four methods of variance comparison (outlined in the Methods) are: i) Observed log10 size variance, calculated without a phylogeny; ii) Mean variance obtained from 1000 simulations; iii) Reveals which habitat contains greater variance by comparing the ratio of observed variances to the ratio of simulated variances (see Methods), which allows us to obtain; iv) the probability of an observed difference in variance relative to

simulations, where dark shades of each colour represent p < 0.05, lighter shades p = 0.1–0.05, and grey p > 0.1. For each approach, two figures are presented – the first emphasising the percentage of results that have fallen in a particular direction for each set of pairwise habitat comparisons within each taxonomic scale. The second figure is identical to the first with the exception that the y axis is free, so that absolute numbers of comparisons (not percentages) can be easily assessed. Statistical output for each comparison is printed within the relevant grid cell.

Appendix 15: All analyses pertaining to comparisons of **size variance** between habitat-use types for the **Catalogue of Fishes 11k-tree dataset.** Plots indicate the percentage or absolute numbers of groups where the size variance of taxa within one habitat-use is greater than the other, obtained for every pairwise habitat-use comparison across multiple taxonomic scales. The four methods of variance comparison (outlined in the Methods) are: i) Observed log10 size variance, calculated without a phylogeny; ii) Mean variance obtained from 1000 simulations; iii) Reveals which habitat contains greater variance by comparing the ratio of observed variances to the ratio of simulated variances (see Methods), which allows us to obtain; iv) the probability of an observed difference in variance relative to simulations, where dark shades of each colour represent p < 0.05, lighter shades p = 0.1–0.05, and grey p > 0.1. For each approach, two figures are presented – the first emphasising the percentage of results that have fallen in a particular direction for each set of pairwise habitat comparisons within each taxonomic scale. The second figure is identical to the first with the exception that the y axis is free, so that absolute numbers of comparisons (not percentages) can be easily assessed. Statistical output for each comparison is printed within the relevant grid cell.

Appendix 16: All analyses pertaining to comparisons of **size variance** between habitat-use types for the **FishBase 31k-tree dataset.** Plots indicate the percentage or absolute numbers of groups where the size variance of taxa within one habitat-use is greater than the other, obtained for every pairwise habitat-use comparison across multiple taxonomic scales. The four methods of variance comparison (outlined in the Methods) are: i) Observed log10 size variance, calculated without a phylogeny; ii) Mean variance obtained from 1000 simulations; iii) Reveals which habitat contains greater variance by comparing the ratio of observed variances to the ratio of simulated variances (see Methods), which allows us to obtain; iv) the probability of an observed difference in variance relative to simulations, where dark shades of each colour represent p < 0.05, lighter shades p = 0.1–0.05, and grey p > 0.1. For each approach, two figures are presented – the first emphasising the percentage of results that have fallen in a particular direction for each set of pairwise habitat comparisons within each taxonomic scale. The second figure is identical to the first with the exception that the y axis is free, so that absolute numbers of comparisons (not percentages) can be easily assessed. Statistical output for each comparison is printed within the relevant grid cell.

Appendix 17: All analyses pertaining to comparisons of **size variance** between habitat-use types for the **Catalogue of Fishes 31k-tree dataset.** Plots indicate the percentage or absolute numbers of groups where the size variance of taxa within one habitat-use is greater than the other, obtained for every pairwise habitat-use comparison across multiple taxonomic scales. The four methods of variance comparison (outlined in the Methods) are: i) Observed log10 size variance, calculated without a phylogeny; ii) Mean variance obtained from 1000 simulations; iii) Reveals which habitat contains greater variance by comparing the ratio of observed variances to the ratio of simulated variances (see Methods), which allows us to obtain; iv) the probability of an observed difference in variance relative to simulations, where dark shades of each colour represent p < 0.05, lighter shades p = 0.1–0.05, and grey p > 0.1. For each approach, two figures are presented – the first emphasising the percentage of results that have fallen in a particular direction for each set of pairwise habitat comparisons within each taxonomic scale. The second figure is identical to the first with the exception that the y axis is free, so that absolute numbers of comparisons (not percentages) can be easily assessed. Statistical output for each comparison is printed within the relevant grid cell.
